# Multi-Omic Data Integration Suggests Putative Microbial Drivers of Aetiopathogenesis in Mycosis Fungoides

**DOI:** 10.3390/cancers16233947

**Published:** 2024-11-25

**Authors:** Philipp Licht, Volker Mailänder

**Affiliations:** 1 Department of Dermatology, University Medical Centre Mainz, 55131 Mainz, Germany; plicht@uni-mainz.de; 2 Max Planck Institute for Polymer Research, 55128 Mainz, Germany

**Keywords:** cutaneous T cell lymphoma (CTCL), Mycosis fungoides (MF), microbiome, transcriptomics, multi-omics, data integration, bioinformatics, computational biology, disease signalling, NF-κB, IL-1B, *Staphylococcus aureus*, protein A, microbiome-driven pathogenesis

## Abstract

Background: Mycosis fungoides (MF) represents the most prevalent entity of cutaneous T cell lymphoma (CTCL). The MF aetiopathogenesis is incompletely understood, due to significant transcriptomic heterogeneity and conflicting views on whether oncologic transformation originates in early thymocytes or mature effector memory T cells. Recently, using clinical specimens, our group showed that the skin microbiome aggravates disease course, mainly driven by an outgrowing, pathogenic *S. aureus* strain carrying the virulence factor spa, which was shown by others to activate the T cell signalling pathway NF-κB. Methods: To explore the role of the skin microbiome in MF aetiopathogenesis, we here performed RNA sequencing, multi-omic data integration of the skin microbiome and skin transcriptome using Multi-Omic Factor Analysis (MOFA), virome profiling, and T cell receptor (TCR) sequencing in 10 MF patients from our previous study group. Results: We observed that inter-patient transcriptional heterogeneity may be largely attributed to differential activation of T cell signalling pathways. Notably, the MOFA model resolved the heterogenous activation pattern of T cell signalling after denoising the transcriptome from microbial influence. The MOFA model suggested that the outgrowing *S. aureus* strain evoked signalling by non-canonical NF-κB and IL-1B, which in turn may have fuelled the aggravated disease course. Further, the MOFA model indicated aberrant pathways of early thymopoiesis alongside enrichment of antiviral innate immunity. In line with this, viral prevalence, particularly of Epstein–Barr virus (EBV), trended higher in both lesional skin and the blood compared to nonlesional skin. Additionally, TCRs in both MF skin lesions and the blood were significantly more likely to recognize EBV peptides involved in latent infection. Conclusions: First, our findings suggest that *S. aureus* with its virulence factor spa fuels MF progression through non-canonical NF-κB and IL-1B signalling. Second, our data provide insights into the potential role of viruses in MF aetiology. Last, we propose a model of microbiome-driven MF aetiopathogenesis: Thymocytes undergo initial oncologic transformation, potentially caused by viruses. After maturation and skin infiltration, an outgrowing, pathogenic *S. aureus* strain evokes activation and maturation into effector memory T cells, resulting in aggressive disease. Further studies are warranted to verify and extend our data, which are based on computational analyses.

## Simple Summary

This research aims to better understand the causes of Mycosis fungoides (MF)—a disease where a type of immune cell, the T cell, malignantly transforms into cancer. It is not yet fully understood what triggers MF or how it progresses, partly because it varies so much between patients and because there is debate about whether the disease begins in immature T cells (thymocytes) or in more mature T cells (memory T cells). Recent findings suggest that bacteria living on the skin, particularly a harmful strain of Staphylococcus aureus, may aggravate MF by triggering specific pathways in T cells. To investigate this hypothesis, we explored the gene expression and microbial abundance of MF patients’ skin with advanced statistical methods. We found that varied microbial skin colonization between patients may explain why the skin gene expression is so different from patient to patient. We also observed additional evidence that *S. aureus* might indeed trigger pathways in mature T cells that fuel cancer progression. Further, our statistical model suggested that certain viruses, like Epstein–Barr virus, could play a role in starting the disease by disrupting thymocytes (immature T cells). Based on these results, we speculate that both perspectives on the origin of MF could be correct, immature (thymocytes) and mature T cells: Thymocytes undergo malignant transformation, possibly caused by viruses, and bacteria like *S. aureus* fuel the malignant T cells to become prominent cancer cells. Our research could help uncover the complex interplay between bacteria, viruses, and T cells in MF. The findings may pave the way for new treatments targeting the skin microbiome or T cell pathways, offering hope for better management of this challenging disease.

## 1. Introduction

Cutaneous T cell lymphoma (CTCL) is a heterogeneous group of non-Hodgkin T cell lymphomas with skin homing properties. The most common entity is Mycosis fungoides (MF), with an incidence rate of 4.1 cases per million in the USA [1,2]. MF patients present with several to many cutaneous lesions that are formed by the infiltration of neoplastic T cells and benign reactive lymphocytes. With disease progression, both neoplastic and benign infiltrates accumulate, resulting in inflammatory reddening of skin lesions [1,3,4]. Depending on the degree of lymphocyte infiltration and inflammation, lesions are classified into the stages patch, plaque, and tumour. As MF is a lymphoproliferative disorder which can involve extracutaneous compartments like the blood, a clinical staging system considers these events and classifies patients into stages IA–IVB [1,5]. In early stages (IA–IIA), MF is an indolent disease with a 5-year disease-specific survival of 89%, which, however, dramatically drops to ~20% in the most advanced stages [5]. Because the aetiology and pathogenesis of MF are incompletely understood, treatment options are limited, and a cure is almost not achievable [6,7].

MF is thought to arise from mature, skin-resident CD4+ T cells [8], which resemble the phenotype of tissue-resident effector memory T cells [9]. However, others suggest that the initial oncologic transformation takes place during early thymopoiesis, specifically during the double-negative (DN) stages DN-1 through DN-3. Those “premalignant clones” may then migrate to the skin, where they proliferate [10,11,12,13,14,15]. The causative agent responsible for the oncogenic transformation of T cells in MF remains uncertain [1]. Viruses are among the potential factors considered due to their involvement in various lymphoma types, including even two other entities of CTCL [16]. Viral involvement in MF is further supported by the elevated risk of MF patients to develop one or more virus-initiated lymphoma types, either simultaneously with MF or later in time [17,18,19,20,21,22,23]. However, the role of viruses in the aetiology of MF remains unclear, as findings from different studies have yielded conflicting results [24,25,26].

Likewise, the molecular drivers of MF pathogenesis remain incompletely understood [27,28]. In benign T cells, three signals orchestrate activity and proliferation: First, an initial T cell response is initiated by antigenic stimulation of the T cell receptor (TCR) together with CD3. Second, co-stimulation is required to augment TCR signalling, which is mediated by various molecules, including CD28 and the tumour necrosis factor receptor superfamily (TNFRSF). Downstream, both TCR signalling and co-stimulation converge on PI3K/AKT, NFAT, and NF-κB [29]. Third, sustained T cell activity is promoted by cytokines, which activate JAK-STAT. While CD8+ T cells require interleukin (IL) 12 and interferon (IFN) α/β to initiate signal three, CD4+ T cells require IL-1 [30,31]. Because these pathways are frequently dysregulated in T cell lymphomas, a “three-signal model” of T cell lymphoma pathogenesis has been proposed [32]. In MF, dysregulation of TCR, TNFRSF/NF-κB, and JAK-STAT pathways is recurrently observed, demonstrating the involvement of all three “T cell lymphoma promoting” signalling pathways [33,34,35,36,37,38]. However, the MF transcriptome exhibits substantial variability between patients and among lesions within the same patient [27,39], which impedes the identification of a shared pathogenic pathway to date.

It has been proposed that the skin microbiome contributes to or evokes transcriptional heterogeneity [40]. In agreement, we recently identified a subgroup of MF patients with a significantly aggravated disease course and outgrowth of a distinct, pathogenic *S. aureus* strain on plaque lesions. Conversely, another MF patient subgroup presented with a more balanced skin microbiome and a favourable prognosis. Reflecting the differing prevalences of *S. aureus* between the two subgroups, we referred to the subgroup with aggravated disease and *S. aureus* outgrowth as ΔSA-positive, while the other subgroup was termed ΔSA-neutral. Notably, the virulence factor staphylococcal protein A (spa) was highly abundant in the genome of *S. aureus* in the ΔSA-positive subgroup [41]. It has been demonstrated that spa activates the NF-κB pathway [42,43], which is involved in T cell co-stimulation [29], and is a component of the “three-signal model” of T cell lymphoma pathogenesis [32]. Moreover, some studies observed aberrant NF-κB activity in subsets of MF patients with aggressive disease [44,45,46]. We thus theorized that the skin microbiome shapes MF disease signalling, resulting in exacerbated malignancy.

To investigate our hypothesis, we here performed bulk RNA sequencing (RNAseq), multi-omic data integration of the microbiome and the transcriptome using Multi-Omic Factor Analysis (MOFA) (48), virome profiling, and T cell receptor sequencing (TCRseq). Our analyses yielded three main findings:

First, our data indicated that inter-patient transcriptional heterogeneity was largely driven by differential expression of pathways involved in T cell signalling. Strikingly, denoising the transcriptome from microbial influence using MOFA pronouncedly reduced the heterogeneous activation pattern of T cell signalling pathways. This advocated that the skin microbiome had a substantial impact on MF disease signalling.

Second, the MOFA model suggested that *S. aureus* with its virulence factor spa induced ectopic activity of both non-canonical NF-κB and IL-1B signalling. While non-canonical NF-κB signalling leads to survival and proliferation of naïve T cells and their differentiation into effector memory T cells (49), IL-1B facilitates sustained CD4+ T cell activation [30,31]. Given that CD4+ effector memory T cells are the malignant T cell subset in MF (*9*), spa-bearing *S. aureus* may induce or augment the phenotypic characteristics of MF, resulting in aggressive disease.

Third, the MOFA model implied augmented antiviral immune response along with enriched pathways involved in early thymopoiesis between DN1 and DN3. Viral prevalence, particularly of Epstein–Barr virus (EBV) and human papillomavirus 71 (HPV), trended higher in both lesional skin and the blood. In line with this, TCRs in both MF skin lesions and the blood were significantly more likely to recognize epitopes of EBV compared to TCRs in nonlesional skin. Notably, the most frequently recognized EBV-epitopes were derived from proteins orchestrating latent EBV infection. Collectively, our findings provide evidence to support potential viral involvement in the aetiology of MF, considering that (I) malignant MF T cells infiltrate the skin from the blood (50), (II) the initial oncologic transformation of malignant T cells in MF was mapped between DN1 and DN3 (10–15), and (III) latent EBV infection can induce non-Hodgkin lymphoma, including peripheral T cell lymphoma [47,48].

Taking findings from our preceding and this study together, we present a speculative model delineating microbiome-driven MF aetiopathogenesis: The initial oncologic transformation occurs during early thymopoiesis, possibly induced by viral infection. Following maturation and skin infiltration, outgrowth of a spa-bearing *S. aureus* strain exacerbates disease by activating both non-canonical NF-κB and IL-1B signalling, resulting in the differentiation of naïve T cells into effector memory T cells with sustained activity. Our study sheds light on the critical role of the microbiome in MF aetiopathogenesis.

## 2. Materials and Methods

### 2.1. Patients and Clinical Specimens

The patient group comprised a subset of patients included in our previous study [41] that were recruited from the Department of Dermatology, University Medical Centre Mainz, Germany. Clinical specimens included metagenomic samples from the skin, skin punch biopsies, and peripheral blood (see Table 1). Metagenomic skin samples were obtained using a swab–scrape–swab procedure described earlier [41]. In brief, a pre-moistened swab was brushed over the skin, then the same skin area was gently scraped with a scalpel, and then it was brushed again with the same swab. To address the interindividual profile of skin microbiomes [49], nonlesional skin from the contralateral site of the sampled lesion was used as a control. The skin biopsies were obtained with 4 mm punches from the same MF lesions sampled for the metagenome and were immediately snap frozen and stored at −80 °C until RNA extraction. Peripheral blood was drawn for the isolation of peripheral blood mononuclear cells (PBMCs) and subsequently enriched for the T cell fraction. For full details, please refer to [41].

### 2.2. RNA Sequencing and Bioinformatic Analysis of the Transcriptome

Total RNA was extracted from skin biopsies and T cell enriched PBMCs as described previously [41]. Total RNA was sent to Novogene Company Ltd. (Cambridge, UK) for library preparation and sequencing. Briefly, after a quality check with Caliper Life Sciences GX II (Hopkinton, MA, USA), 400 ng RNA was used as input for the NEBNext Ultra RNA Library Prep Kit (New England Biolabs, Ipswich, MA, USA). One sample (nonlesional skin of Pat1) was excluded due to low RNA quality. After the quality check, libraries were sequenced on a NovaSeq 6000 (Illumina, San Diego, CA, USA) at 150 bp paired-end to an average of 78.74 (range 63.82–94.20) million raw reads, with 11.81 (range 9.57–14.13) giga base pairs (Gbp) per sample.

For the transcriptome analysis, raw reads were quality-checked with Fastqc version 0.11.9 [50], and subsequently aligned against the human reference genome Ensembl built GRCh38.p13 using STAR 2.7.9a with the flag –quantMode GeneCounts [51]. The data were loaded into DESeq2 version 1.36.0 [52], filtered for genes with a minimum of ten cumulative counts over all samples, and normalized using the internal DESeq2 method. Subsequently, a differential expression analysis using the Wald test was carried out, adjusted for influences from individual patients which may have been introduced by the paired-sample design. To account for multiple testing, *p*-values were adjusted using Benjamini–Hochberg and genes with <0.05 adjusted *p*-value were considered significant. Volcano plots were created using the R package EnhancedVolcano [53]. For principal component analyses, the data were first transformed with the variance-stabilization method (vst) and then analysed using the plotPCA function within DESeq2. The PCA was visualized using ggplot2 [54].

Gene set enrichment analysis was performed using the R package pathfindeR [55]. Log2 fold-changes of significantly deregulated genes determined by DESeq2 were used as input. The Reactome database [56] was used to find enriched pathways. Because we were particularly interested in signalling pathways that might orchestrate MF pathogenesis, we screened the literature for pathways that were described as deregulated in T cell lymphomas or CTCL. The Reactome database was subsequently filtered to retain only pathways that contain at least one of the following terms (case-insensitive): cascade, signal, signalling, signaling, pathway, transition, cycle, regulation, activation, keratinization, cornified, antimicrobial, interferon, IFN, stimulation, stimuli, activate, receptor, TLR. Enriched pathways were clustered using the pathfindeR function cluster_enriched_terms. Plots were created using pathfindeR.

Upregulated pathways in the plaque stage that, according to the literature, may have been elicited by *S. aureus* were further investigated using PCA. Therefore, the influence from the paired-sample design was regressed out from the normalized and rlog-transformed count matrix with the removeBatchEffect function from the limma package [57]. For three differentially regulated pathways of interest in the plaque stage, up- and downregulated genes according to pathfindeR were determined. The corrected count matrix was filtered for the genes of interest and plaque stage samples and parsed into the prcomp R stats function to perform PCA. ggplot2 was used for visualization.

### 2.3. Multi-Omic Data Integration

We utilized the R implementation of MOFA (Multi-Omic Factor Analysis) version 1.6.0 [58] to integratively analyse the microbiome and transcriptome datasets. MOFA integrates multiple omics datasets by discovering latent factors that capture both shared and omic-specific patterns of variation. These latent factors help to identify key biological signals, denoise the data, and identify relationships between different omics layers.

The microbiome dataset was created in our preceding study where we characterized the MF skin microbiome using shotgun metagenomics and MetaPhlAn 3.0.2 [41,59,60]. To obtain absolute counts of microbial taxa (rather than the relative abundance, which is the default in MetaPhlAn and was used in our previous study), the metagenome was re-profiled including the flag -t rel_ab_w_read_stats using MetaPhlAn 3.0.2 with the intermediate bowtie2 mapping files as input. The resulting profiles were filtered to retain only taxa on the species level and normalized using the wrench method, which accounts for sparse metagenomic count data and is implemented in the R package wrench [61]. The normalized data were log2-transformed with a pseudocount of 1. To select highly variable species for the MOFA model, 50 species with the highest variance were filtered (with the R base function var) and used for all subsequent steps.

The transcriptome dataset was created in this study as described above. After mapping and normalization with STAR 2.7.9a and DESeq2 version 1.36.0 (described above), the data were transformed with the regularized logarithm using the rlog function implemented in DESeq2 [52]. As recommended by the MOFA authors, the large transcriptome dataset was restricted to not overrepresent the smaller microbiome dataset in the MOFA model. Therefore, the top 5000 highly variable genes were selected based on the median absolute deviation calculated with the R base function mad. The MOFA model was trained with a single group, the option scale_views set to TRUE, convergence_mode set to slow, and eight factors.

Gene set enrichment analysis was performed with the MOFA function run_enrichment, which is based on principal component gene set enrichment (PCGSE) [62] using only genes with positive weights as input. The Reactome database, filtered for pathways of interest (see the above section of transcriptome analysis), was used as a background. Pathway enrichment plots were generated with ggplot2.

For the heatmaps in Figure 2A,B, an identical set of genes were plotted using the R package pheatmap (refer to the caption of Figure 2 for more details on gene selection). The heatmap in Figure 2A shows the normalized gene counts, while the heatmap in Figure 2B shows the denoised version of the data, explained by the latent factors of the MOFA model. This means that the MOFA model reconstructs the input data (transcriptome and microbiome) based on the latent factors and shows patterns that are shared between the omics layers and that are omics-specific. The denoised heatmap in Figure 2B was generated with the MOFA function plot_data_heatmap with argument denoise set to TRUE. Internally, plot_data_heatmap uses pheatmap for heatmap plotting, with the MOFA trained data as input. The code used for Figure 2B (and other figures) can be found in the Data Availability Statement.

### 2.4. Virus Profiling

The virome present on superficial skin layers was profiled via re-analysis of the microbiome dataset generated in our previous investigation [41]. Briefly, human skin was brushed topically with swabs, and whole metagenomic sequencing was performed and subsequently profiled with MetaPhlAn 3.0.2. While viruses were excluded in our previous study, here we activated the flag for viral identification (--add_viruses). The resulting profiles were filtered to retain only viruses. 

The virome present in deeper skin layers was profiled using RNAseq reads generated from skin punch biopsies. In contrast to the swabbing procedure, punch biopsies obtain the entire depth of skin layers. Virome profiles from RNAseq reads were generated with the VIRTUS pipeline [63]. Briefly, non-human reads were filtered out through alignment to the human reference genome Ensembl built GRCh38.p13. Next, unmapped (i.e., non-human) reads were aligned against 762 viral genomes. Heatmaps visualizing the virome profile were generated with the R package pheatmap [64].

### 2.5. Assessment of TCR–Epitope Binding

To investigate whether T cells of MF patients recognize EBV-epitopes, the T cell receptor was sequenced as described previously [41]. Briefly, RNA isolated from skin punch biopsies and T cell enriched PBMCs of MF patients were subjected to library preparation spanning the variable part of the TCR using the NEBNext Immune Sequencing Kit, human (E6320, New England Biolabs, USA), and sequenced on a MiSeq (Illumina, USA) running 300 bp PE. The sequencing data were processed with the pRESTO toolkit [65] (https://usegalaxy.org/u/bradlanghorst/w/presto-nebnext-immune-seq-workflow-v320, accessed on 20 May 2019) and the R package immunearch [66]. EBV-epitopes were obtained from the Immune Epitope Database (IEDB) (https://www.iedb.org/, accessed on 2 April 2019) [67]. The Tool ERGO-II [68] was used to determine binding scores of pairs of TCRs and EBV-epitopes. The closer the binding score is to 1, the higher the probability that a TCR recognizes the epitope [68].

## 3. Results

### 3.1. Study Group and Clinical Specimens

The study group consisted of 10 MF patients (mean age at sampling: 64.7 years; range: 47 to 82 years) and was a subset of patients that were enrolled in our preceding investigation where we characterized the MF skin microbiome [41]. To ensure traceability, IDs of included patients in this study were adopted from our previous investigation [41]. In that study, we found that a subgroup of patients presented with an increased abundance of *S. aureus* on MF lesions compared to nonlesional skin of the same patient. We termed this subgroup ΔSA-positive as opposed to the ΔSA-neutral subgroup, where *S. aureus* abundance was consistent between MF lesions and nonlesional skin of the same patient. The ΔSA-positive subgroup had a strong dysbiosis and a significantly aggravated disease course compared to the ΔSA-neutral subgroup [41]. Of the 10 MF patients included in the present study, 3 were in the ΔSA-positive subgroup and 7 were in the ΔSA-neutral subgroup.

Clinical specimens included skin swabs and punch biopsies of lesional skin (5 patch stage and 5 plaque stage) and matched nonlesional skin of the same MF patients as an intra-patient control (10 nonlesional skin samples). For more details about the rationale of using internal controls, please refer to Licht, Dominelli et al. [41]. Additionally, blood was drawn in four patients for the isolation of peripheral blood mononuclear cells (PBMCs). All clinical specimens were taken at the same visit. Skin punch biopsies were used for RNAseq. Where sufficient material was available, skin punch biopsies and PBMCs were used for TCRseq. Shotgun metagenomics from skin swabs were initially carried out in our previous investigation [41] and re-analysed in the present study in a multi-omic data integration approach together with the skin transcriptome. Table 1 summarizes patient metadata, clinical specimens, and data modalities used in the present study.

### 3.2. Transcriptional Heterogeneity May Be Largely Driven by Differential Activation of Pathways of the “Three-Signal Model” of T Cell Lymphoma Pathogenesis

A number of studies investigated the CTCL transcriptome [27,39] and uncovered various mechanisms of disease progression [33,34,35,36,37,38]. However, the transcriptome was also found to exhibit significant heterogeneity among patients, complicating the identification of a common pathological mechanism [27,39,46,69,70,71,72,73].

**Figure 1:**
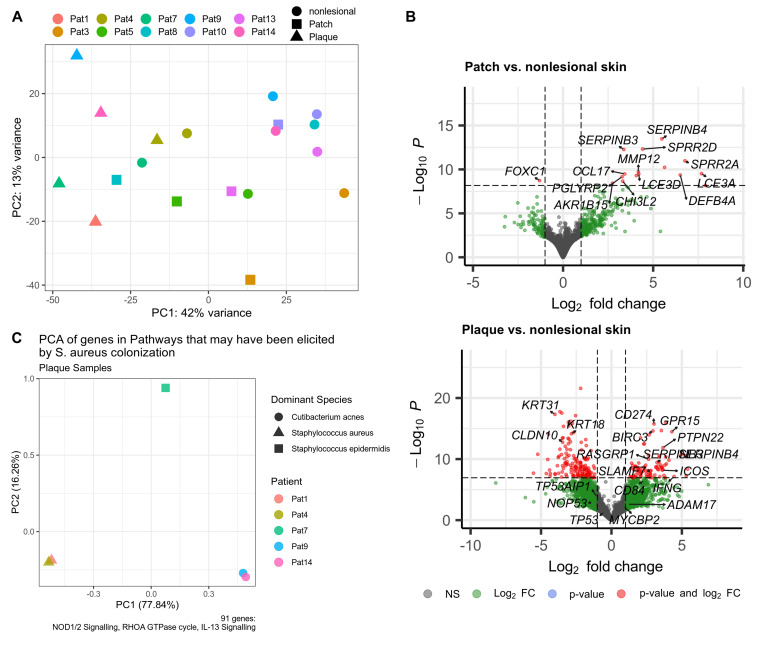
Analysis of the MF skin transcriptome. (**A**) Principal component analysis (PCA) showing inter-heterogeneity of plaque. (**B**) Volcano plots visualizing the distribution of gene fold-changes and statistical significance between patch or plaque and nonlesional skin. For the complete results table of DESeq2 differential gene expression analysis, refer to Supplementary Material S1. (**C**) PCA of genes belonging to upregulated pathways in the plaque stage that may have been elicited by *S. aureus* according to the literature [75,76,77,78,79]. PC = principal component, NS = not significant.

To explore the root of transcriptional heterogeneity, we employed differential gene expression analysis and gene set enrichment analysis (GSEA). Principal component analysis (PCA) of the MF skin transcriptome clearly separated nonlesional skin, patch, and plaque via principal component (PC) 1. However, lesional samples also spread along PC2, demonstrating strong inter-patient transcriptional heterogeneity (Figure 1A). Likewise, GSEA recovered several aberrant pathways known to promote MF exacerbation, such as chemokine signalling [74] (Supplementary Material S2), but also showed substantial activation differences between patients in both patch and plaque lesions (Supplementary Material S3). Notably, pathways of the “three-signal model” of T cell lymphoma pathogenesis exhibited different activation patterns between patch and plaque (Table 2): TCR signalling was enriched in both patch and plaque, whereas interleukin-13 (IL-13) signalling, CD28 co-stimulation, and co-stimulation through death receptors activating NF-κB [44,45] were enriched solely in the plaque stage.

**Figure 2:**
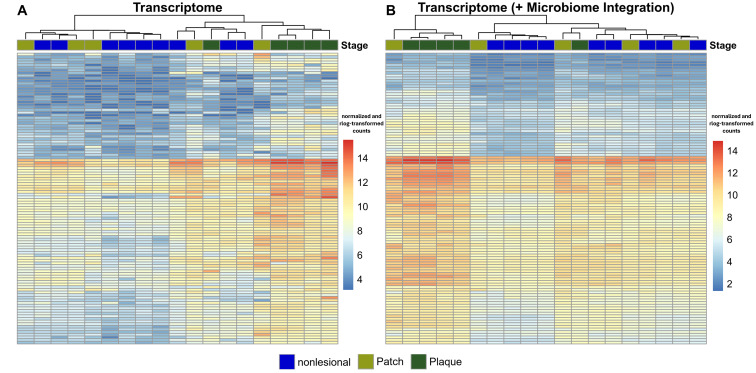
Clustered heatmap of genes involved in the “three-signal model” of T cell lymphoma pathogenesis. The clustered heatmap was created with genes involved in the “three-signal model” of T cell lymphoma pathogenesis that were differentially expressed in the patch and/or plaque stage and were included in the MOFA model (5000 highly variable genes; see Methods and Supplementary Material S6). Raw gene counts were normalized using the median of ratios and rlog-transformed with the DESeq2 package [52]. (**A**) Shown are normalized RNA expression levels before integration of the microbiome. Notably, although only genes of the “three-signal model” of T cell lymphoma pathogenesis were included, the clustering resembled the patterns of the PCA, which was based on the entire transcriptome. Despite clustering together, plaque samples exhibited a high degree of heterogeneity. (**B**) After data integration of the transcriptome with the microbiome, the heterogeneity of plaque samples was largely resolved, suggesting that the microbiome had a strong impact on MF disease signalling. Please refer to the Methods section for information on how the denoised heatmap was generated.

Next, we aimed to assess whether pathways of the “three-signal model” for T cell lymphoma pathogenesis [32] replicate transcriptional heterogeneity. To this end, we compiled a list of genes that are (I) members of the “three-signal model” for T cell lymphomas and (II) were differentially expressed in either or both patch and plaque stages. A clustered heatmap of these genes closely resembled the pattern of the PCA of the entire transcriptome (Figure 2A): Nonlesional skin and plaque samples formed separate clusters, while patch samples exhibited intermediate expression levels. Importantly, plaque samples displayed pronounced heterogeneity in RNA expression levels (Figure 2A). We thus reasoned that inter-patient transcriptional heterogeneity can, at least to some extent, be attributed to differential expression of pathways that orchestrate T cell lymphoma pathogenesis.

### 3.3. The MF Skin Transcriptome Shows Responses to Microbial Stimuli

Since differential microbial skin colonization may contribute to transcriptional heterogeneity [40], and we recently identified that the skin microbiome stratifies MF patients and determines the clinical course [41], our next objective was to identify microbial responses in the skin transcriptome.

As expected, we uncovered enriched pathways likely activated by the skin microbiome (Table 2 and Supplementary Material S2). Notably, Toll-Like Receptor 4 (TLR4) was upregulated in patch and showed attenuated activity in plaque. The pathway senses lipopolysaccharides, a cell wall constituent of Gram-negative bacteria, and activates inflammatory responses as well as pyroptosis, which is a form of programmed necrosis. Pyroptosis protects the host from microbial infection but, if overactivated, can also lead to pathological inflammation [80], a typical condition of progressive MF [4]. In accordance, we recently showed that the skin microbiome of patches is strongly dysbiotic, while dysbiosis on plaques was observed only on a subgroup of MF patients [41], providing a potential explanation for the differential TLR4 activation between patch and plaque.

Conversely, several microbe-associated pathways were uniquely upregulated in plaque lesions, potentially driven by the outgrowth of a distinct S. aureus strain harbouring virulence factors such as α-hemolysin and spa [41]. The innate immune sensor NOD2 (nucleotide-binding oligomerization domain 2) recognizes small peptides derived from the peptidoglycan cell wall component of Gram-positive bacteria like *S. aureus*. Importantly, NOD2 mediates protective responses specifically against *S. aureus* through its interaction with the virulence factor α-hemolysin [75,76]. Additionally, we observed enhanced activity of the RHOA GTPase cycle, a pathway that *S. aureus* can exploit via its virulence factor spa to facilitate epithelial invasion [77]. Further, we also noted enrichment of IL-13 signalling, consistent with reports that IL-13 expression is induced in healthy skin upon *S. aureus* exposure [78,79]. Interestingly, malignant T cells in the skin of MF patients express IL-13, whereas malignant T cells found in lymph nodes and blood do not [46]. To further investigate whether the upregulation of NOD1/2 signalling, the RHOA GTPase cycle, and IL-13 signalling might have been elicited by the microbiome, as suggested by the literature, we conducted PCA of genes regulating these pathways (Figure 1C). Plaque samples with *S. aureus* as the dominant species in the microbiome clearly separate from those with other dominant species.

Collectively, we noted several enriched pathways that may be attributed to microbial stimuli, consistent with the microbiome patterns identified on MF lesions in our preceding study [41]. Consequently, our findings indicated that distinct microbial colonization led to differential transcriptomic responses between patients.

### 3.4. Multi-Omic Data Integration of the Microbiome and the Transcriptome Resolves Transcriptional Heterogeneity

To investigate whether differential skin colonization elicited the heterogeneous expression of genes related to the “three-signal model” for T cell lymphoma pathogenesis, we performed data integration of the microbiome and the transcriptome using Multi-Omic Factor Analysis (MOFA) [58]. Briefly, MOFA can be seen as a multi-omic implementation of PCA and finds latent factors (comparable to principal components in PCA) that capture the main sources of variation across different omic data modalities (which are called views in the MOFA framework). Within the latent factors, weights are allocated to the features of the different views. Consequently, latent factors are characterized by feature weights, which indicate their importance or significance in the variation captured by the latent factor. Downstream, additional analyses such as gene set enrichment analysis (GSEA) can be conducted within each latent factor. The MOFA framework further enables the identification of each view’s contribution to the variation captured by a latent factor across the entire multi-omic dataset [58].

**Figure 3:**
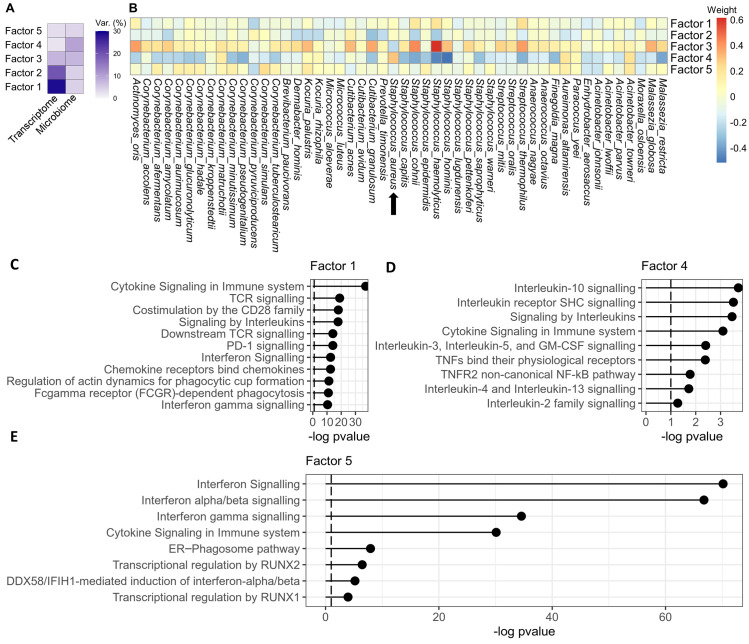
Overview of the latent factors of the MOFA model in the microbiome and transcriptome data modalities. (**A**) Overview of the latent factors capturing variances in the microbiome and transcriptome data modalities. Factor 4 captured the majority of variance in the microbiome, meaning that this factor captured important impacts of the microbiome on the transcriptome. (**B**) Overview of the feature weights of the microbiome per factor. (**C**–**E**) Gene set enrichment analysis. Factor 1 showed pathomechanisms of high and sustained T cell activity in MF (TCR signalling, CD28 co-stimulation, interleukin signals). Factor 4 showed pathways that were likely evoked by *S. aureus*. Factor 5 showed strong antivirus responses along with enrichment of RUNX1 and RUNX2 signalling, which have pivotal roles in thymopoiesis [81,82,83,84].

Regarding the multi-omic dataset in this study, the weights of the microbes (features of the microbiome) and the weights of the genes (features of the transcriptome) characterize the latent factors. The MOFA model showed a good fit to the multi-omic dataset, as the patterns of the latent factors were congruent to the results observed in both the independent transcriptome analysis (see above) and our previous study on the MF skin microbiome [41] (see Supplementary Material S4). Latent factors 1 to 5 captured shared sources of variation present in both data modalities, indicating a reciprocal influence between the transcriptome and microbiome (Figure 3A). By leveraging the latent factors, MOFA reconstructs the input data to separate shared and specific variations in each data modality, thereby denoising the data and revealing underlying biological signals [58]. Strikingly, the differential expression of genes involved in the “three-signal model” of T cell lymphoma pathogenesis was resolved after integration of the microbiome and transcriptome data modalities (called denoised in MOFA2 terms; Figure 2B). This indicated that the lesion-specific microbiome heavily influences MF disease signalling. Please refer to the Methods section for information how the denoised heatmap was generated.

### 3.5. Spa-Bearing S. aureus Likely Activates Non-Canonical NF-κB and IL-1B Signalling

We next assessed the impact of the microbiome on MF disease signalling in more detail. **Factor 4** accounted for a substantial proportion of the total variance in the microbiome (Figure 3A), and *S. aureus* was the only microbiome feature with a positive weight. Notably, microbes with anti-*S. aureus* properties, such as *S. hominis* and *S. epidermidis* [85,86,87], displayed markedly negative weights. Thus, the microbiome feature weights exhibited a pattern similar to plaque lesions of the ΔSA-positive subgroup. These patients presented with *S. aureus* outgrowth and an aggravated clinical course [41].

In the transcriptome, the following pathways controlling T cell activity were enriched: Interleukin signalling, particularly IL-1B, and non-canonical NF-κB signalling (Figure 3D, Supplementary Material S5). While non-canonical NF-κB serves as a co-stimulator and generates and maintains effector memory T cells [88,89], IL-1B signalling facilitates sustained activation of CD4+ T cells [32]. In agreement, the malignant T cell subset in MF is thought to be CD4+ effector memory T cells [9]. Regarding IL-1B signalling, Chng et al. [90] reported that human keratinocytes express IL-1B when challenged with *S. aureus*. This suggests that outgrowth of virulent *S. aureus* may stimulate the tumour microenvironment to activate malignant T cells via IL-1B in a paracrine fashion.

Regarding non-canonical NF-κB signalling, we intriguingly observed that the stimulating ligand, TNF-α [91], displayed an almost neutral weight in factor 4 (Supplementary Material S5), indicating an alternative stimulation route. Instead, we previously identified high abundance of the virulence factor staphylococcal protein A (spa) in the genome of the *S. aureus* strain colonizing ΔSA-positive patients. It was reported that spa can activate the NF-κB pathway [42,43], and some studies associated aggressive CTCL with upregulated NF-κB signalling [44,45,46]. Notably, several genes emphasized in these studies exhibited positive weights in factor 4 (e.g., *LTA*, *LTB*, *BIRC3*, *TNFSF13*, *TNFSF14*, *TNFRSF1B*, and *TNFRSF7*; Supplementary Material S5). Owing to the almost neutral weight of the NF-κB stimulating agent TNF-α and the presence of spa as an alternative stimulator [41], we theorized that outgrowing, spa-bearing *S. aureus* strains upregulated non-canonical NF-κB signalling in MF patients with aggressive disease.

Collectively, the MOFA model indicated that the skin microbiome significantly influences MF disease signalling and is a major source of transcriptional heterogeneity. Further, the MOFA model suggested that *S. aureus* fuelled malignant T cells to promote the aggravated disease course of ΔSA-positive patients via two signalling axes: While spa may activate non-canonical NF-κB signalling, resulting in survival, proliferation, and naïve T cell differentiation into mature, effector memory T cells, the neoplastic T cell subset in MF [9], paracrine IL-1B signalling might facilitate sustained activation.

### 3.6. Aberrant Signalling of Early Thymopoiesis Alongside Enriched Antiviral Immunity Suggests Viral Involvement in MF Aetiology

Additionally to factor 4, our attention was drawn to the results of **factor 5**. While the microbiome weights showed only minimal signals (Figure 3B), GSEA identified significant upregulation of host defence signals against viruses (Figure 3E), indicating that factor 5 represented a source of variation independent of bacteria. In particular, GSEA showed upregulation of interferon alpha and beta (IFN-α/β) signalling, which is the first line of innate immune defence upon viral infection. Downstream, IFN-α/β signalling stimulates genes that inhibit the replication machinery of viruses at various mechanistic levels [92]. Several of these interferon-stimulated genes exhibited high weights in factor 5, for example *MX1*, *IFIT1*, *IFIT3*, *OAS1*, *OAS2*, *IFI27*, and *OASL* (Supplementary Material S5). *IFIT3*, which displayed the highest weight in factor 5, was shown to specifically boost antiviral signalling by IFN-α/β [93]. Further, GSEA found an enrichment of the ER–phagosome pathway (Figure 3E), which can be hijacked by viruses for their own translation, replication, and particle budding in order to spread into other host cells [94]. Notably, the phagosome-pathway genes *TAP1* and *TAP2*, which displayed high weights in factor 5 (Supplementary Material S5), are highly expressed by Epstein–Barr virus (EBV)-infected lymphocytes [95], and interact with Epstein–Barr-nuclear antigen 1 (EBNA1) [96].

Remarkably, EBV infection can lead to *RUNX1* expression [97,98], and signalling by *RUNX1* and *RUNX2* was significantly enriched in factor 5 (Figure 3E). The RUNX family is a frequent target of retroviral insertion [99,100,101], resulting in the development of several T cell lymphoma entities [102,103]. *RUNX1* regulates the expansion of mature CD4+ T cells [82], which constitutes the neoplastic T cell subset in MF [9]. Interestingly, both *RUNX1* and *RUNX2* are important regulators of early thymopoiesis during the double-negative stages of T lymphocytes [81,82,83,84], a developmental step that has been mapped to the initial oncologic transformation of T cells in MF [10,11,12,13,14,15].

Since the transcriptome dataset included in the MOFA model was restricted to 5000 genes (see Methods section), we screened the entire transcriptomic dataset to investigate signals of aberrant thymopoiesis in more detail. It has been reported that ectopic expression of *RUNX2* strongly expands immature thymocytes during the double-negative stages, resulting in a preneoplastic state of thymocytes characterized by low proliferation rates [81]. However, concomitant overexpression of *MYC* rescues proliferation and facilitates differentiation. Additionally, *MYC* and *RUNX* collaboratively inhibit the tumour suppressor p53, resulting in decreased apoptosis of malignant T cells. Together, this ultimately leads to the accumulation of mature, neoplastic T cells [81,104]. In agreement with these reports, several genes were aberrantly expressed in the MF transcriptome (Supplementary Material S1): Besides *RUNX1* and *RUNX2*, we identified enriched *MYCBP2* (Figure 1B), which is a member of the c-myc family with the function to increase c-myc activity [105]. Further, although the pathway to regulate p53 activity was upregulated (Table 2), important components of the p53 machinery were downregulated (Figure 1B): These included the p53 gene *TP53* itself, the p53 stabilizing protein *NOP53* [106], and TP53AIP1, which is regulated by p53 and induces apoptosis [107]. A complete results table of DESeq2 differential gene expression analysis is available in Supplementary Material S1.

In summary, our data show dysregulation of pathways involved in early thymopoiesis, probably representing the initial oncologic transformation of T cells in MF. Moreover, factor 5 suggested a connection between viruses and MF aetiology given the concomitant upregulation of host responses to viruses and *RUNX1/2* signalling.

### 3.7. Increased Viral Prevalence and EBV-Epitope Recognition in MF Skin Lesions

In MF, malignant T cells circulate in the blood and infiltrate the upper dermis but have not been described to be present in the most superficial layers of the skin [108]. To determine whether viruses may be involved in in MF aetiology, we investigated viral prevalence in different layers of the skin and the blood of MF patients. To this end, we examined whole metagenomic sequencing (WMS) and RNAseq data. While the WMS data were generated from skin swabs and thus only contain material from superficial skin layers, the RNAseq data were generated from both the blood and skin punch biopsies representing the whole skin, including deeper skin layers like the epidermis and dermis.

**Figure 4:**
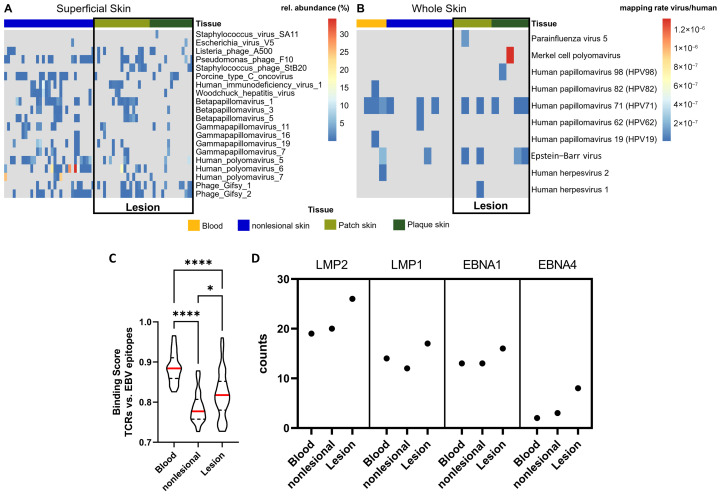
Viral association with MF. (**A**,**B**), Viral prevalence identified in WMS reads of superficial skin (**A**) and RNAseq reads of whole skin (**B**). Viral prevalence in MF lesions of whole skin trended higher compared to nonlesional skin, specifically EBV and HPV71 (**B**), whereas no difference was present on superficial skin (**A**). In (**A**), the top 20 viruses with the highest variation between MF lesions and nonlesional skin are shown. In (**B**), all viruses detected are shown. In (**C**,**D**), T cells in MF lesions and the blood specifically recognize EBV-epitopes. (**C**) The probability of TCRs in blood, nonlesional skin, and MF lesions to recognize EBV-epitopes was assessed using ERGO-II [68]. Binding scores (1 = perfect binding, 0 = no binding) were calculated for each TCR with each EBV-epitope. n = 175; displayed are violin plots with the median (red), 1st quartile, and 3rd quartile. Kruskal–Wallis test: * for *p* ≤ 0.05, **** for *p* ≤ 0.0001. (**D**) Displayed are the EBV-epitopes that were recognized most often by TCRs of MF patients.

In whole skin samples, total viral load trended higher in MF lesions compared to nonlesional skin (Figure 4B). In contrast, total viral load did not differ in superficial skin layers (Figure 4A). Among all viruses detected in MF lesions of whole skin samples, EBV and human papillomavirus 71 (HPV71) displayed the highest prevalence, albeit HPV71 was also frequently present in nonlesional skin. Remarkably, EBV and HPV71 were also present in the blood of some patients (Figure 4B). Both EBV and various human papillomaviruses are implicated in the development of cancer, including T cell lymphomas [109,110,111]. Since MF is characterized by the infiltration of circulating T cells from the blood into the skin [108], the concomitant presence of viruses in both compartments indicates a potential viral involvement in MF.

Next, we evaluated whether T cells of whole skin samples or T cells in the blood were directed against known EBV-epitopes obtained from the Immune Epitope Database (IEDB) [67]. We sequenced the TCRs of MF lesions, nonlesional skin, and blood and utilized ERGO-II [68] to calculate binding scores for the most abundant TCRs of a given sample with each of the EBV-epitopes. TCRs in MF lesions and the blood showed a significantly higher probability to recognize EBV-epitopes than T cells in nonlesional skin (Figure 4C), which agreed with the increased viral prevalence of MF lesions in whole skin samples. Interestingly, latent membrane protein (LMP) 1, LMP2, and Epstein–Barr-nuclear antigen (EBNA) 1 were the most frequently recognized EBV-epitopes by TCRs in the skin and blood (Figure 4D). An expression pattern of LMP1, LMP2, and EBNA1 represents a characteristic EBV gene profile of latency type I/II, commonly observed in EBV-induced T cell lymphomas [112,113]. In NK/T cell lymphoma, LMP1 upregulates *CD274* (PD-L1) via the NF-κB axis [114], which agreed with our results of enriched *CD274* and NF-κB signalling in the plaque stage (Table 2, Figure 1B).

Together, the trend of increased EBV and HPV71 prevalence in whole skin samples along with a significantly higher probability of EBV-epitope recognition by TCRs in MF lesions and the blood compared to nonlesional skin suggest viral involvement in MF aetiology.

## 4. Discussion

The clinical course of MF varies greatly, with some patients having only minor progress, whereas others suffer from fast progression and high disease burden [5,115]. Likewise, the transcriptome is tremendously heterogeneous, exhibiting patient and even lesion specificity [27,39,73]. Moreover, ambiguity exists about the initial oncologic transformation of malignant T cells, as studies from different groups mapped the event to either early thymocytes [10,11,12,13,14,15] or mature, effector memory T cells [9]. Consequently, a common pathogenic mechanism of all patients or patient subgroups is still in doubt, resulting in a non-optimal treatment regimen [27]. We recently demonstrated that the skin microbiome of MF patients is altered, and identified a subgroup of patients that was overgrown with a distinct, pathogenic *S. aureus* strain. This subgroup exhibited a significantly aggravated disease course, possibly owing to the virulence factor spa, which was present in the *S. aureus* genome [41]. In line with this, others showed that spa can activate the NF-κB axis [42], which is recurrently deregulated in MF patients with aggressive disease [34,44,45,46,116]. We thus theorized that (I) the lesion-specific microbiome determines transcriptomic response, thereby contributing to or causing heterogeneity [40], and that (II) spa-bearing *S. aureus* elicits NF-κB signalling to fuel MF. Therefore, we investigated how the skin microbiome affects the skin transcriptome in a subset of 10 MF patients that were enrolled in our preceding study [41]. Using RNAseq, multi-omic data integration, virome profiling, and TCRseq, we obtained novel insights into the role of the microbiome in both the aetiology and pathogenesis of MF.

First, we recovered substantial transcriptomic heterogeneity on both gene and pathway levels and observed that this heterogeneity may be largely driven by differential expression of T cell signalling pathways. Strikingly, our results suggested that the differential T cell signalling pattern may have been caused by the skin microbiome, since denoising the transcriptome with the microbiome using MOFA considerably reduced heterogeneity (Figure 2A,B).

Second, latent factor 4 implied that *S. aureus* elicited upregulation of both non-canonical NF-κB and IL-1B signalling, which could explain the aggravated disease course of MF patients overgrown with a spa-bearing *S. aureus* strain. Non-canonical NF-κB signalling is known to promote survival and proliferation of thymocytes and mature T cells, induce differentiation of naïve T cells into effector memory T cells, and support clonal expansion [88,89], which are all common characteristics of malignant T cells in MF [1,8,9,117]. NF-κB signalling is typically initiated by the interaction of TNFα with either TNFRSF1A (also known as TNFR1) or TNFRSF1B (also known as TNFR2) [88,89]. However, the enrichment of non-canonical NF-κB signalling in factor 4 appeared to be independent of TNFα, since it displayed a very low weight. Instead, we propose that the *S. aureus* virulence factor spa induced non-canonical NF-κB signalling since Gómez et al. showed that spa activates TNFRSF1A [42,43]. In general, TNFRSF1A induces the canonical form of NF-κB signalling leading to apoptosis, while TNFRSF1B induces non-canonical NF-κB signalling resulting in survival and proliferation. However, there is some level of crosstalk between the two forms of NF-κB pathways. In highly activated T cells, such as the malignant T cells in MF, activation of TNFRSF1A results in survival rather than apoptosis [88,89,118]. Further, the extracellular domains of TNFRSF1A and TNFRSF1B exhibit a high degree of structural similarity [119], possibly allowing spa to activate both receptors. Additionally, while TNFRSF1A is expressed nearly ubiquitously across various cell types throughout the body, TNFRSF1B expression is considerably more restricted, including thymocytes and T cells [89]. Thus, spa may activate either TNFRSF1A, TNFRSF1B, or both to initiate non-canonical NF-κB signalling to fuel MF progression.

In agreement with our findings, a clinical study reported that systemic inhibition of NF-κB in CTCL patients induced a skin response in 30.4% of patients, whereas the blood response was mixed [120,121,122] Further, Shin et al. identified ectopic NF-κB signalling in 30.6% of MF patients and that these patients had an aggravated disease course compared to patients without ectopic NF-κB signalling [44]. We previously identified that 31.3% of MF patients were overgrown with the virulent, spa-bearing *S. aureus* strain and had an aggravated disease course [41]. Considering the comparable prevalences of the specified subgroups and the presumed NF-κB-activating effect of spa, this may suggest a potential association between skin colonization by a spa-carrying *S. aureus* strain and exacerbation of the disease in the aforementioned studies. Additional research on the spa-NF-κB interaction including mechanistic assays with bigger patient groups is warranted.

Regarding our finding of upregulated IL-1B signalling in factor 4, it was shown that *S. aureus* induces secretion of IL-1B by eosinophils [123], which infiltrate MF lesions [124]. Furthermore, human keratinocytes, skin-derived dendritic cells, and lymphocytes produce several cytokines, including IL-1A, IL-1B, and IL-4, when challenged with *S. aureus* or spa [90,125,126]. This suggests a paracrine stimulation of malignant T cells by the tumour microenvironment via cytokine signalling in response to *S. aureus*.

Last, we discovered evidence indicating viral involvement in the aetiology of MF. Latent factor 5 captured a concomitant upregulation of innate antivirus defence mechanisms and aberrant *RUNX1/2* signalling. The latter is known to coordinate thymopoiesis during double-negative (DN) stages DN1 through DN3 [81,82,127], which have been mapped to the initial oncologic transformation in MF [10,11,12,13,14,15]. We further observed that viral load, particularly of HPV71 and EBV, trended higher in both deeper skin layers of MF lesions and the blood compared to nonlesional skin, whereas superficial skin layers of MF lesions showed no difference. HPV71 was shown to degrade p53 [128], which can result in neoplasia [129]. Nevertheless, while certain papillomaviruses are categorized as high-risk factors for the onset of solid cancers [130] and have also been loosely linked to an elevated risk of lymphomas [131], HPV71 is generally regarded as having low oncogenic potential [132]. In contrast, EBV is a well-known oncovirus [133,134,135]. Remarkably, we found that T cells residing in MF lesions or in the blood were significantly more affinitive to EBV-epitopes than T cells of nonlesional skin (Figure 4C). Moreover, the most frequently recognized EBV-epitopes were epitopes of LMP1, LMP2, and EBNA1, which are genes commonly expressed by EBV in latent infection [113]. Although EBV has a strong B cell tropism, leading to B cell Hodgkin’s lymphoma (HL) and non-Hodgkin’s lymphoma (nHL) [133,134,135], the virus also infects T cells, thereby causing some entities of T cell nHL [47,48]. Notably, CTCL patients have an increased risk of developing secondary HL and nHL [17,18,19,20]. It has been proposed that a single precursor malignant T cell can trigger HL, CTCL, and lymphomatoid papulosis, a benign T cell neoplasm, in the same patient [21,22]. Others reported the simultaneous presence of HL and CTCL within the same lymph node and theorized that the two malignancies arise from distinct B and T cells [23], indicating that a common trigger induced oncogenesis.

To the best of our knowledge, we were the first to investigate viral prevalence in different skin compartments and found that EBV and HPV71 trended higher solely in deeper skin layers of MF lesions, where malignant T cells in MF reside [108]. Collectively, our data indicate that viruses, probably EBV and/or HPV71, might play a role in MF aetiology. However, the sample size was rather small and not all lesional and blood samples were positive for EBV and/or HPV71. Moreover, despite a significantly higher EBV-epitope recognition of these tissues defined by computational estimation, mechanistic assays are warranted for a definitive conclusion. Thus, hypotheses on viral involvement in MF need to be considered speculative at this point. Additional investigations with more participants and longitudinal viral monitoring across tissues and different skin compartments are necessary to determine whether viruses are the aetiologic agent in MF.

An open question remains whether the age distribution of our patient group may have influenced the skin condition/clinical appearance, since 7 out of 10 patients were aged 60 or older (4 ΔSA-neutral, 3 ΔSA-neutral). In skin-disease-free subjects, several skin integrity parameters such as hydration, sebum production, or cytokine production decrease with age [136,137]. Studies in MF have indicated that skin integrity and moisture levels are lower in comparison to age-matched healthy subjects, which could lead to an increased susceptibility to bacterial infection [138,139,140]. This implies that older MF patients could experience a more severe skin barrier defect than younger MF patients and hence could experience infections more frequently. Notably, a cut-point of 60 years of age has been identified as a clinically prognostic outcome factor. Given that 70% of the patients in our study group were aged 60 and older and were evenly distributed between the two ΔSA groups, this indicates appropriate age matching within our study group. However, bigger patient groups with a more diverse population are warranted.

Further limitations of our study are the small patient group size, the mono-centric nature of our study, and the lack of mechanistic assays to confirm our findings, which are based on computational analyses from primary clinical specimens.

Additionally, the skewed distribution of patients between the two ΔSA groups could have introduced bias. To mitigate this concern, we specifically opted to utilize MOFA2, which is an unsupervised method agnostic of grouping variables. Analogous to PCA, MOFA2 generates clusters based on integrated multi-omic profiles [58]. Therefore, the input to MOFA2 entails the entire group rather than a comparison of two (potentially imbalanced) groups. Importantly, despite the smaller size of the ΔSA-positive group compared to the ΔSA-neutral group, the MOFA2 model identified substantial upregulation of both non-canonical NF-κB and IL-1B signalling, which was largely disregarded by conventional GSEA (Supplementary Material S2, plaque vs. nonlesional skin). Nonetheless, our findings are based on a small patient group derived from our preceding study [41]. To assess the robustness of the MOFA2 model, we performed a thorough evaluation of (a.) the stability of our model throughout iterations and (b.) the robustness of our model against technical noise. Our assessment suggests that MOFA robustly detects the same latent factors and patterns with and without noise. Please refer to Supplementary Material S7 for the full stability assessment. The current study represents an in-depth investigation of a rather limited patient group, and further research is required for validation and generalization.

## 5. Conclusions

In this study, we provided novel insights into the role of the microbiome in MF aetiopathogenesis. First, our results indicated that the skin microbiome largely contributes to transcriptional heterogeneity. Second, our data suggest that a spa-bearing *S. aureus* strain, which overgrows a subgroup of MF patients with aggravated disease course, might evoke non-canonical NF-κB and IL-1B signalling in the skin. Third, our data collectively suggested that viruses, particularly EBV and HPV71, may be the aetiologic agent in MF.

**Figure 5:**
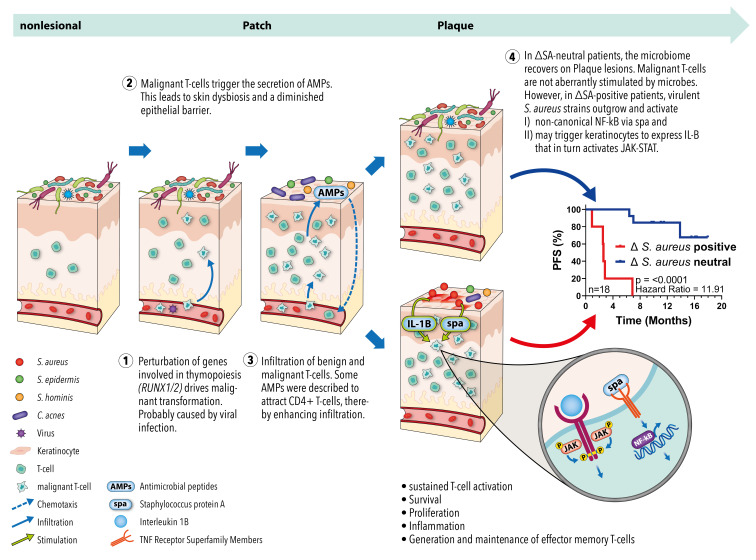
Proposed model of microbiome-driven MF aetiopathogenesis. T cell precursors undergo initial oncologic transformation due to aberrant expression of RUNX1 and RUNX2, probably caused by viruses like EBV and/or HPV71. After maturation and skin infiltration, the malignant T cells trigger the microenvironment to secrete AMPs. AMPs kill most of the physiological skin microbiota, resulting in dysbiosis and a diminished epithelial barrier. In addition, AMPs may recruit benign and malignant CD4+ T cells, establishing a loop of sustained dysbiosis and T cell infiltration. Since AMP levels remain constant over the disease course, some microbes eventually acquire resistance and recolonize the lesions. In the ΔSA-neutral subgroup, microbes with anti-S. aureus activity accumulate, resulting in a balanced microbiome that does not fuel the disease. However, in the ΔSA-positive subgroup, virulent S. aureus strains bearing the virulence factor spa overgrow and activate non-canonical NF-κB as well as IL-1B signalling. The two pathways cause inflammation, sustained activity, survival, and proliferation of T cells, as well as the generation and maintenance of effector memory T cells. These characteristics are hallmarks of malignant T cells in MF and may explain the significantly aggravated clinical course of the ΔSA-positive subgroup.

Together with the results from our preceding study, these findings led us to propose a model of microbiome-driven MF aetiopathogenesis (Figure 5): The initial oncologic transformation could emerge during early thymopoiesis triggered by aberrant *RUNX* expression, potentially caused by viral infection such as with EBV and/or HPV71. Malignant T cells infiltrate the skin, leading the microenvironment to release AMPs. Eventually, this process eliminates microbes, resulting in skin dysbiosis and a diminished epithelial barrier. Additionally, certain AMPs attract CD4+ T cells, which could include malignant T cells, thereby augmenting the infiltration of (malignant) T cells into MF lesions. Over time, some microbes acquire resistance to AMPs and recolonize the lesions. In the ΔSA-neutral subgroup, microbes with anti-*S. aureus* properties accumulate, resulting in a balanced microbiome and favourable clinical course. However, in the ΔSA-positive subgroup, a virulent *S. aureus* strain carrying the virulence factor spa outgrows and activates non-canonical NF-κB and IL-1B signalling, resulting in the generation of mature effector memory T cells and poor outcome. Hence, both perspectives on the cells of origin in MF may apply: early T cell progenitors [10,11,12,13,14] and mature, effector memory T cells [8,9].

Additional research is warranted to validate and generalize our findings, which are based on a rather small patient group. Furthermore, research allowing mechanistic insights is needed to understand how the microbiome affects malignant T cells in MF.

## Figures and Tables

**Table 1:** Study group, clinical specimens, and patient metadata. This study group is a subset of the group enrolled in Licht et al. [41], in which we characterized the skin microbiome of MF patients. We demonstrated that *S. aureus* abundance is increased on MF lesions compared to nonlesional skin in a subgroup of patients (ΔSA-positive), while S. aureus abundance does not change in the other subgroup (ΔSA-neutral). ΔSA-positive patients exhibit a poor clinical course compared to ΔSA-neutral patients. Patient IDs in this study match the given patient IDs in Licht et al. [41]. NA = not available; f = female; m = male.

Patient ID	Sex	Age (Years)	Clinical Stage	ΔSA- Subgroup	Stage of Lesion (Body Site)	RNAseq: Tissues Included	TCRseq: Tissues Included
Pat1	f	81	IA	positive	plaque (hip)	lesion, blood	lesion, blood
Pat3	f	47	IB	neutral	patch (thigh)	lesion, nonlesional	NA
Pat4	m	70	IIB	positive	plaque (upper back)	lesion, nonlesional, blood	lesion, nonlesional, blood
Pat5	f	73	IB	neutral	patch (thigh)	lesion, nonlesional	lesion, nonlesional
Pat7	m	54	IB	neutral	plaque (flank)	lesion, nonlesional, blood	lesion, nonlesional
Pat8	m	82	IIB	positive	patch (upper back)	lesion, nonlesional, blood	lesion, blood
Pat9	m	63	IB	neutral	plaque (forearm)	lesion, nonlesional	lesion
Pat10	m	49	IB	neutral	patch (gluteus)	lesion, nonlesional	lesion
Pat13	m	66	IB	neutral	patch (lower leg, back)	lesion, nonlesional	NA
Pat14	m	62	IB	neutral	plaque (lower leg, front)	lesion, nonlesional	lesion, nonlesional, blood

**Table 2:** Gene set enrichment analysis. Displayed are selected pathways of interest. Please refer to Supplementary Material S2 for the full GSEA list. log2fc = log2 fold-change, NA = not available, padj = *p*-value adjusted to multiple comparisons using Bonferroni.

Pathway	Patch log2fc	padj	Plaque log2fc	padj
Keratinization	6.35	3.6 × 10^−5^	1.18	7.1 × 10^−8^
Antimicrobial peptides	8.69	1.4 × 10^−5^	NA	NA
TCR signalling	4.74	1.3 × 10^−7^	1.63	1.4 × 10^−14^
CD28 co-stimulation	NA	NA	1.74	1.1 × 10^−5^
Non-canonical NF-kB pathway	NA	NA	1.34	4.3 × 10^−11^
Interleukin-13 signalling	NA	NA	1.41	6.2 × 10^−4^
IFN-γ signalling	8.32	6.7 × 10^−5^	1.83	5.8 × 10^−3^
Death receptor signalling	NA	NA	1.85	6.4 × 10^−21^
Toll-Like Receptor 4 (TLR4) Cascade	2.55	1.5 × 10^−2^	1.31	6.4 × 10^−19^
NOD1/2 signalling pathway	NA	NA	1.84	4.3 × 10^−17^
RHOA GTPase cycle	NA	NA	1.62	4 × 10^−29^
Regulation of TP53 activity	NA	NA	1.28	1.8 × 10^−10^

## Data Availability

The code used to generate the plots in Figure 1, Figure 2, Figure 3 and Figure 4A,B, Supplementary Figure S1, and Supplementary Material S5 was deposited on GitHub (https://github.com/phlicht/Multi-Omic_Data_Integration_in_MF). All datasets used in this study (i.e., RNAseq, WGS, and TCRseq) were deposited on the Gene Expression Omnibus (GEO) under the SuperSeries GSE221150 (https://www.ncbi.nlm.nih.gov/geo/query/acc.cgi?acc=GSE221150): RNA sequencing data and associated analysis files can be accessed under GSE221148 (https://www.ncbi.nlm.nih.gov/geo/query/acc.cgi?acc=GSE221148). This includes viral profiles from both whole skin biopsies and the blood. Microbiome sequencing data and associated analysis files can be accessed under GSE221149 (https://www.ncbi.nlm.nih.gov/geo/query/acc.cgi?acc=GSE221149). This includes re-analysed microbiome profiles with absolute counts and viral profiles from superficial skin layers. TCR Sequencing data and associated analysis files can be accessed under GSE218874 (https://www.ncbi.nlm.nih.gov/geo/query/acc.cgi?acc=GSE218874).

## Supplementary Materials

The following supporting information can be downloaded at https://www.mdpi.com/article/10.3390/cancers16233947/s1: Supplementary Material S1: Differentially expressed genes in patch and plaque vs. nonlesional skin; Supplementary Material S2: Gene set enrichment analysis of patch and plaque vs. nonlesional skin; Supplementary Material S3: Heterogeneous activation pattern of pathways between nonlesional skin and patch or plaque, respectively; Supplementary Material S4: Characterization of the fitted MOFA2 model; Supplementary Figure S1: Total variance explained in the transcriptome and the microbiome data modalities by the MOFA model. Supplementary Material S5: Gene set enrichment analysis of each MOFA2 factor; Supplementary Material S6: Transcriptome input for the MOFA2 model. Supplementary Material S7: Stability Assessment of the MOFA model.
