# Supplementary material for: Multi-Omic Data Integration Suggests Putative Microbial Drivers of Aetiopathogenesis in Mycosis Fungoides
Source: Cancers (Basel). 2024 Nov 25;16(23):3947. doi: 10.3390/cancers16233947 (PMC11640326; doi:10.3390/cancers16233947)
Supplement: Supplementary file 1 [file cancers-16-03947-s001.zip › Suppl_Material7.pdf]

Stability Assessment of the MOFA model

Contents

Rationale 2

Summary 2

load libraries 3

1. Stability of the MOFA model over multiple iterations 3

1.1 Comparison of the latent factors between models . . . 5

Interpretation . . . 7

1.2 Comparison of the evidence lower bound (ELBO) . . . 7

2. Stability of the MOFA model against technical noise 7

2.1 Data Distribution . . . 8

2.2 Function to generate negative binomial noise . . . 10

2.3. Estimate mean and dispersion for both the transcriptome and microbiome . . . 10

2.3.1 Transcriptome . . . 10

2.3.2 Microbiome . . . 11

2.4 Representation of Datasets with added Noise in Lower Space (Dimensionality Reduction) . . . 12

2.4.1 Transcriptome . . . 12

2.4.2 Microbiome . . . 15

2.5 Interpretation of Dimensionality Reduction Plots . . . 19

2.6 Run the MOFA model with the perturbed data . . . 19

2.7 Analysis of MOFA models with perturbed data . . . 22

2.7.1 Comparison of explained variance . . . 22

Cumulative explained variance per **view** . . . 22

Interpretation . . . 26

Explained variance per **factor and view** . . . 26

Interpretation . . . 29

2.7.2 Comparison of latent Factors . . . 29

Comparison of latent Factors aggregated across samples to a single value per latent factor . . . 29

Interpretation . . . 31

Latent Factors per sample across noise levels 31
Interpretation 33
Interpretation 37
2.7.3 Comparison of ELBO values 37
Interpretation 38

References **38**

Rationale

The rationale of this document is to investigate the stability of the MOFA model fit to the two datasets (called views in the MOFA framework) Microbiome and Transcriptome of the paper *"Multi-Omic Data Integration Suggests Putative MicrobialDrivers of Aetiopathogenesis in Mycosis fungoides"*. Two kind of assessments will be shown:

1. MOFA fits the input data iteratively until it converges to a point without further improvement as measured by the evidence lower bound (ELBO). Since this process is locally and not globally (Argelaguet et al. (2018)), we will run the original MOFA model of this manuscript iteratively and proof that similar latent factors are found in each iteration.
2. Assessment of the MOFA model against technical noise.

Summary

Because of the length of this document, we opted to include the summary section here.

We performed a thorough evaluation of the robustness of the MOFA model shown in the manuscript regarding stability over multiple iterations as well as artificially introduced technical noise. Several matrices were compared.

To investigate whether the manuscript's model converged on a global scale, we performed 10 iterations and assessed:

- Latent factors of each model iteration, aggregated to a single value across samples
- The Evidence Lower Bound (ELBO) of each model iteration

Both the correlation of each model iteration's latent factors and the ELBO plot show the exact same results for all model iterations. This proofs that the model converged on a global scale, suggesting a good fit to the data.

To investigate the manuscript's model robustness to artifical technical noise, we introduced noise with 15 different magnitudes to the input data and performed 10 iterations on each noise level. We assessed:

- Dimensionality Reduction (PCA for transcriptome and PCoA for microbiome) of…
  - the full datasets
  - only for the features that were selected for the original manuscript's MOFA model (i.e., 5000 genes, and 50 microbes)
- Explained variance

  - aggregated as **cumulative** explained variance **per view**
  - broken down into explained variance per **view *and* factor**

- Latent factors
  - aggregated to a single value per latent factor for each model evaluated as pearson correlation and unsupervised clustering
  - sample-wise latent factors evaluated with unsupervised clustering
  - sample-wise latent factors, aggregated to median values (the median latent factor value per sample calculated from the 10 iterations per noise level) evaluated with unsupervised clustering

- The evidence lower bound (ELBO)

The dimensionality reduction scatter plots (PCA and PCoA) show that the features selected for the MOFA model are stable against technical noise, albeit the microbiome is less robust. We attribute this to the fact that it is more complex to simulate meaningful noise for the way smaller and sparse/zero-inflated microbiome dataset compared to the transcriptome dataset. The analysis of explained variance, latent factors, and the ELBO collectively suggest that the MOFA model is very robust to technical noise at noise levels `c(0.01, 0.1)`. Increasing noise levels still provide overall stable results, but also show increasing skewness and decreasing explained variance. However, evaluation of the latent factors show that factors 1 and 2 are highly robust over all noise levels, and that factor 4 (the factor most investigated in our manuscript) is robust in lower noise levels `c(0.01, 0.1, 0.5, 1, 1.33, 1.66, 2)` and partly also in higher noise levels. As factor 4 captured the majority of variation in the microbiome, and generating meaningful noise for the microbiome is highly complex, we gauge this outcome as evidence for model robustness.

Collectively, the stability assessment of this document strongly indicates a good fit of the MOFA model to our data, the transcriptome and the microbiome, despite the relatively small sample size. This result is in line with the recommendations of the authors of MOFA2, who propose a minimal sample size of 15.

load libraries

```
library(tidyverse)
library(MOFA2)
library(ggpubr)
library(DESeq2)
library(pheatmap)
library(ggsignif)
```

1. Stability of the MOFA model over multiple iterations

MOFA fits the input data iteratively until it converges to a point without further improvement as measured by the evidence lower bound (ELBO). Since this process is locally and not globally (Argelaguet et al. (2018)), the manuscript's MOFA model may not be stably converged. To investigate, we will run the manuscript's MOFA model iteratively (10 times) and inspect key metrics.

```
# Set options for running MOFA
data_options <- list(
  scale_views = TRUE,
  scale_groups = FALSE,
  center_groups = TRUE,
  use_float32 = FALSE,
```

```r
  views = c("Transcriptome", "Microbiome"),
  groups = "group1"
)

model_options <- list(
  likelihoods = c(Transcriptome = "gaussian", Microbiome = "gaussian"),
  num_factors = 8,
  spikeslab_factors = FALSE,
  spikeslab_weights = TRUE,
  ard_factors = FALSE,
  ard_weights = TRUE
)

training_options <- list(
  maxiter = 2000,
  convergence_mode = "slow",
  drop_factor_threshold = -1,
  verbose = FALSE,
  startELBO = 1,
  freqELBO = 5,
  stochastic = FALSE,
  gpu_mode = FALSE,
  seed = 42,
  outfile = NULL,
  weight_views = FALSE,
  save_interrupted = FALSE
)

# Define number of iterations
n_iter <- 10
# Set list for saving
MOFA_val_out <- list()

for (i in 1:n_iter) {
  cat("Running Iteration: ", i, "of 10\n")
  y <- paste0("Iteration_", i)

  # Set MOFA object
  MOFA_validation <- create_mofa(list(
    # The DESeq2 normalized, rlog transformed transcriptome counts.
    # 5000 highly variable genes selected by stats::mad().
    Transcriptome = Transcriptome_rlog_5000mad,
    # The wrench normalized, log2 transformed microbiome counts.
    # 50 highly variable microbes selected by stats::var().
    Microbiome = Microbiome_wrenchNormed_log2_50var
  ))

  # Prepare MOFA object
  MOFA_validation <- prepare_mofa(
    object = MOFA_validation,
    data_options = data_options,
    model_options = model_options,
    training_options = training_options
```

```
  )

  # Run MOFA
  MOFA_validation <- run_mofa(MOFA_validation)

  # Save in list
  MOFA_val_out[[y]] <- MOFA_validation
}
```

1.1 Comparison of the latent factors between models

We now inspect the latent factor values of the validation models and the mansucript's original MOFA model. Therefore, latent factor value per sample is aggregated into a single value per latent factor of each model and then correlated using pearson correlation. The pearson coefficent is then plotted in a heatmap with unsupervised clustering. If the models generate similar latent factors, the heatmap should look like a block diagonal matrix, suggesting that all factors are robustly detected in all model instances.

```
models <- append(MOFA_val_out, c(original_model = MOFAobject))

LFs <- lapply(seq_along(models), function(i) {
  do.call(rbind, get_factors(models[[i]]))
})
if (is.null(Reduce(intersect, lapply(LFs, rownames))))
  stop("No common samples in all models for comparison")
samples_names <- Reduce(intersect, lapply(LFs, rownames))
LFs <- lapply(LFs, function(z) {
  z[samples_names, , drop = FALSE]
})
for (i in seq_along(LFs)) colnames(LFs[[i]]) <- paste(names(models)[i],
                                                      colnames(LFs[[i]]), sep = "_")
corLFs <- cor(Reduce(cbind, LFs), use = "complete.obs")
corLFs[is.na(corLFs)] <- 0
corLFs <- abs(corLFs)

annot_row <- corLFs %>%
  as.data.frame() %>%
  rownames_to_column("rownames") %>%
  dplyr::select(rownames) %>%
  mutate(
    Factor = str_extract(rownames, "(?<=_)[^_]+$"),
    Model = case_when(
      str_detect(rownames, "original_model") ~ "original_model",
      TRUE ~ "Validation"
    )
  ) %>%
  column_to_rownames("rownames")
annot_col <- corLFs %>%
  t() %>%
  as.data.frame() %>%
  rownames_to_column("rownames") %>%
  dplyr::select(rownames) %>%
  mutate(
```

```r
    Factor = str_extract(rownames, "(?<=_)[^_]+$"),
    Model = case_when(
      str_detect(rownames, "original_model") ~ "original_model",
      TRUE ~ "Validation"
    )
  ) %>%
  column_to_rownames("rownames")

ann_colors <- list(
  Factor = RColorBrewer::brewer.pal(8, "Set1"),
  Model = c("white", "green")
)
names(ann_colors$Factor) <- annot_col$Factor %>% unique
names(ann_colors$Model) <- annot_col$Model %>% unique

compare_factors(
  models,
  show_rownames = FALSE,
  show_colnames = FALSE,
  annotation_row = annot_row,
  annotation_col = annot_col,
  annotation_colors = ann_colors
)
```

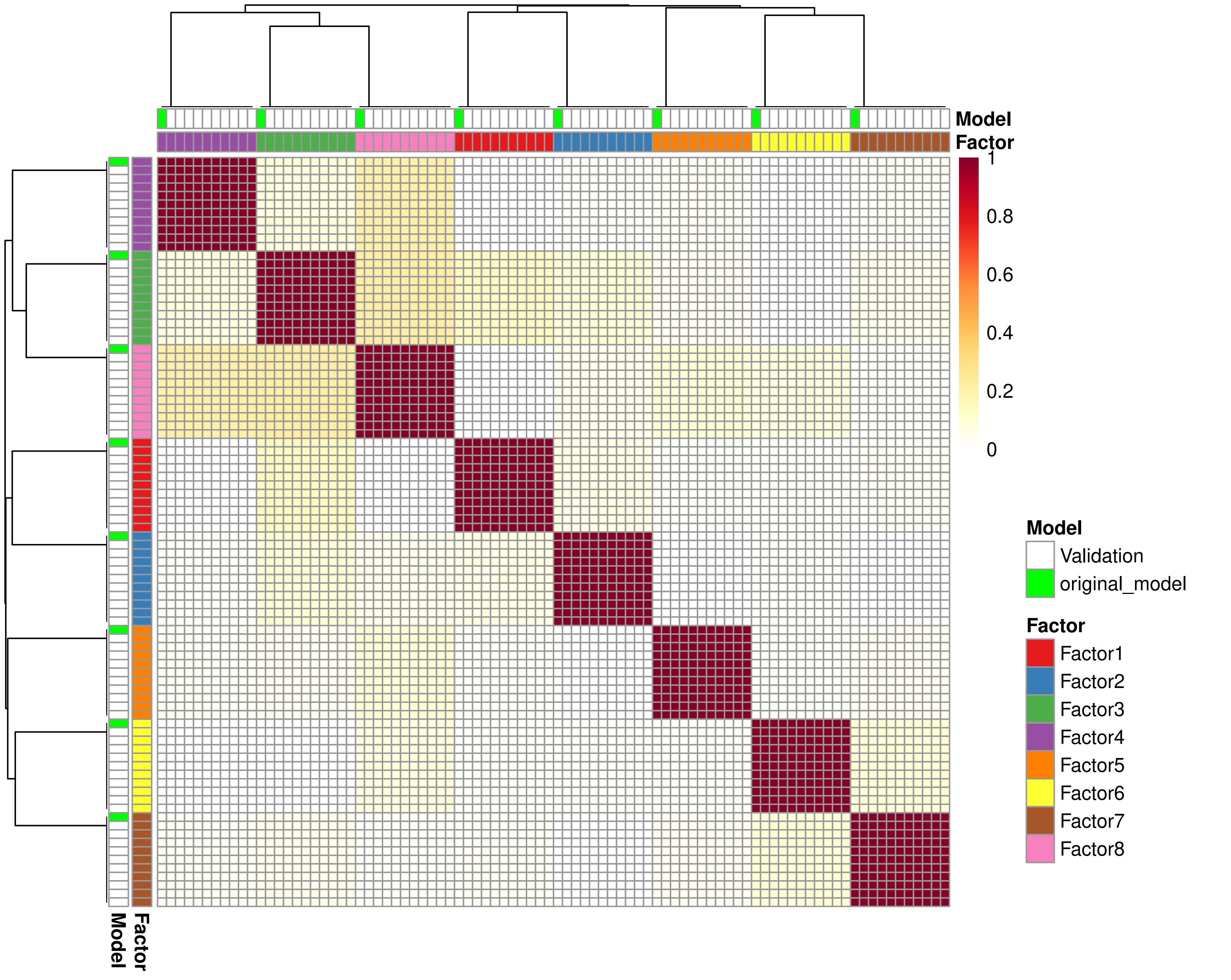

Model
Factor
1
0.8
0.6
0.4
0.2
0
Model
Validation
original_model
Factor
Factor1
Factor2
Factor3
Factor4
Factor5
Factor6
Factor7
Factor8
Model
Factor

Interpretation

We see that the MOFA model converges to exact same latent factor values in each iteration, indicating a robust fit of the model.

1.2 Comparison of the evidence lower bound (ELBO)

```
compare_elbo(models) +
  ggtitle("Absolute ELBO value for each model. The higher the value, the better the fit.",
          subtitle = "All models have exact same ELBO value, showing robust fit of the MOFA model to the
  theme(axis.text.x = element_text(angle = 90, hjust = 1, vjust = 0.2))
```

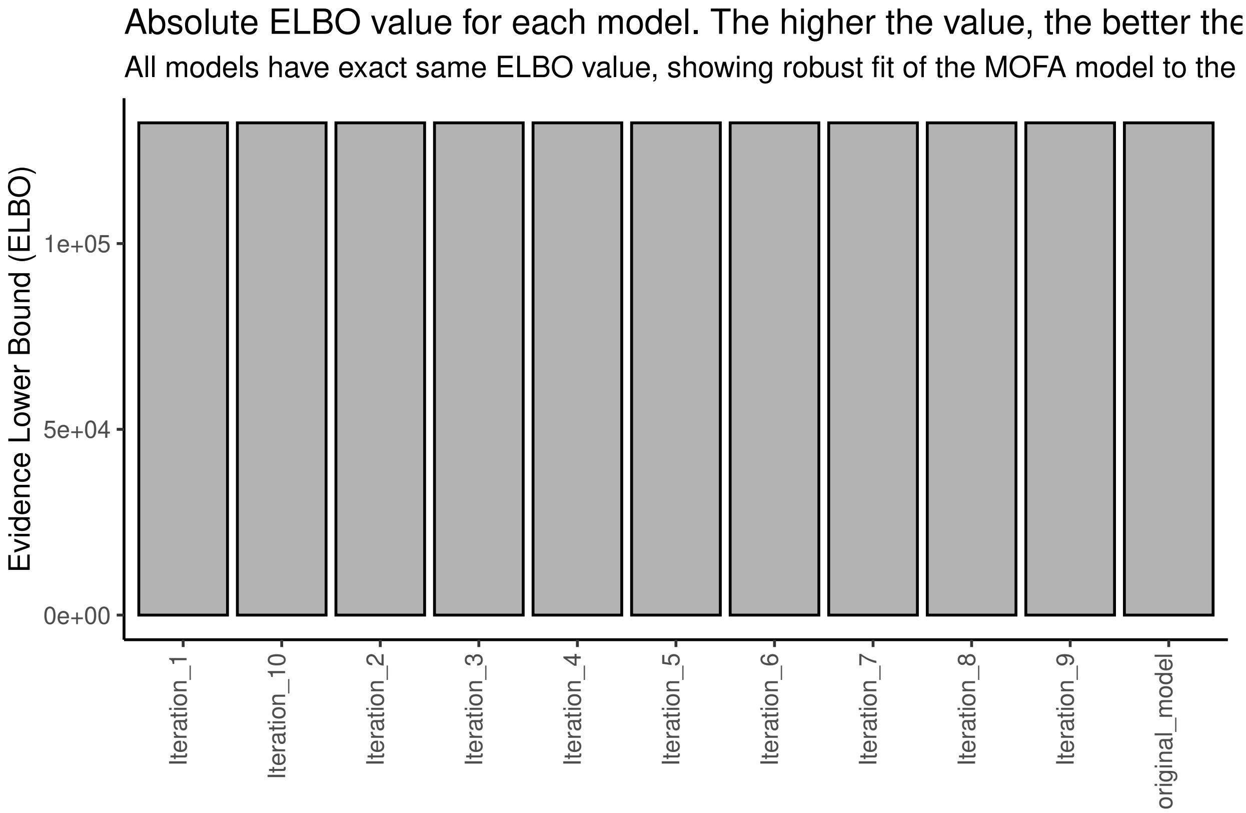

Interpretation All models have exact same ELBO value, showing robust fit of the MOFA model to the data.

2. Stability of the MOFA model against technical noise

RNA sequencing and Whole Metagenome sequencing come with a certain degree of uncertainty due to technical variation during sampling, RNA/DNA-extraction, library preperation, and sequencing. This is particularly true for the skin microbiome: First, sampling captures high and varying percentages of human DNA, leading to varying sequencing depths (Tett et al. (2017), Licht et al. (2024)). Second, contamination with microbiota present in the laboratory or on the researchers skin is not easy to prevent (Kong et al.

(2017)). Third, the skin microbiome is highly interindividual (Oh et al. (2014)), leading to a noisy and sparse (zero-inflated) microbome abundance matrix (Mallick et al. (2021)).

To assess the stability of the MOFA model against noise, we estimated the technical noise in the two views of our MOFA model, transcriptome and microbiome respectively, and added different levels of the respective noise to the raw count matrices of the transcriptome and microbiome.

NOTE: We want to stress that generation of technical noise is complicated. Our data is composed of nonlesional skin and lesional skin, with the latter being composed of patch and plaque stage. Further, some patients were overgrown by *S. aureus*. Thus, our data inherits compositional structures that may be detected as technical noise, but may be rather correct measurements.

2.1 Data Distribution

To simulate technical noise with the same distribution as the raw input data, we first inspect the distribution of the raw input data.

```
# Transcriptome
dist_transcriptome <- Transcriptome_raw %>%
    as.data.frame() %>%
    tidyr::pivot_longer(cols = dplyr::all_of(colnames(Transcriptome_raw))) %>%
    ggpubr::gghistogram(
        "value",
        y = "..density..",
        bins = 300,
        fill = "lightblue",
        xlim = c(0, 1e+05),
        title = "Transcriptome: Distribution of raw counts"
    )

mean_var_transcriptome <- tibble(
  x = matrixStats::rowVars(as.matrix(Transcriptome_raw)),
  y = rowMeans(as.matrix(Transcriptome_raw))
) %>%
  ggplot(aes(
    x = x,
    y = y
  )) +
  geom_point() +
  xlab("Variance") +
  ylab("Mean") +
  theme_minimal() +
  ggtitle("Transcriptome: Mean-Variance Plot")

# Microbome
dist_microbiome <- Microbiome_raw %>%
  as.data.frame() %>%
  tidyr::pivot_longer(cols = dplyr::all_of(colnames(Microbiome_raw))) %>%
  ggpubr::gghistogram(
    "value",
    y = "..density..",
    bins = 30000,
    fill = "lightblue",
    xlim = c(0, 500),
```

```
    title = "Microbiome: Distribution of raw counts"
  )

mean_var_microbiome <- tibble(
  x = matrixStats::rowVars(as.matrix(Microbiome_raw)),
  y = rowMeans(as.matrix(Microbiome_raw))
) %>%
  ggplot(aes(
    x = x,
    y = y
  )) +
  geom_point() +
  xlab("Variance") +
  ylab("Mean") +
  theme_minimal() +
  ggtitle("Microbiome: Mean-Variance Plot")

ggarrange(dist_transcriptome, dist_microbiome, mean_var_transcriptome, mean_var_microbiome)
```

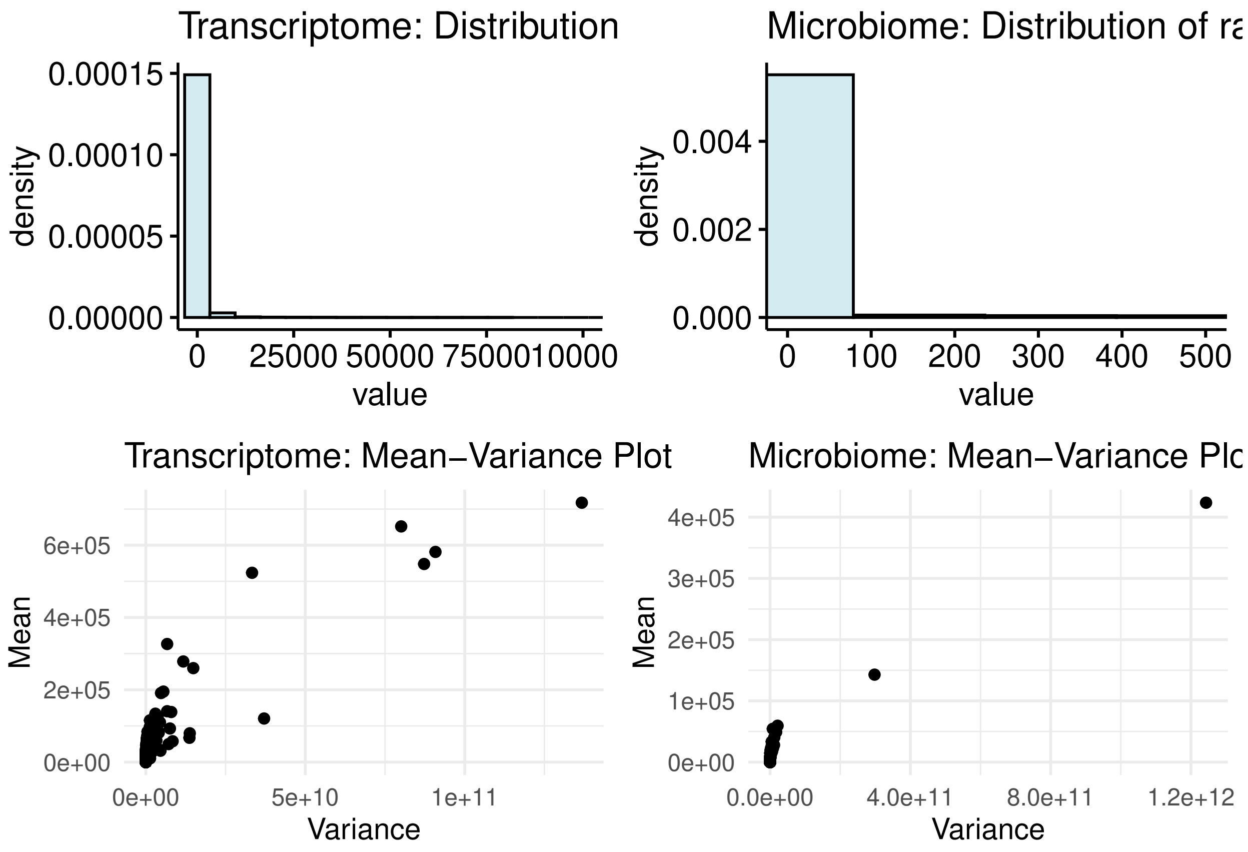

Both the transcriptome and the microbiome follow a negative binomial distribution as shown by the histograms and the mean-variance plots, where the variance is greater than the mean.

2.2 Function to generate negative binomial noise

We now define a function to randomly generate count data that follows a negative binomial distribution. We will feed the function with values from our datasets transcriptome and microbiome (i.e., mean and dispersion). We also add a "leverage" to adjust the noise to different levels. Reationale for a "leverage" of noise is that we cannot estimate how much noise is "too much" so that the MOFA model will be unable to find meaningful signals. We will calculate the MOFA model for the following dispersion/noise levels: `c(0.01, 0.1, 0.5, 1, 1.33, 1.66, 2, 3, 4, 5, 6, 7, 8, 9, 10)`.

```
generate_nb_noise <- function(mean, dispersion, disp_leverage = 1, n_samples) {
  dispersion <- dispersion * disp_leverage # leverage the dispersion/noise to different levels
  size <- 1 / dispersion  # Convert dispersion to size parameter of stats::rnbinom
  mu <- mean
  noise <- rnbinom(n_samples, size = size, mu = mu)
  return(noise)
}
```

2.3. Estimate mean and dispersion for both the transcriptome and microbiome

We separately estimate for both the transcriptome and the microbiome. We do so by using the lowest expressed/abundant features in each dataset after an initial filtering step that removes removes features that are almost not present throughout the dataset (this is a conventional step in the field).

2.3.1 Transcriptome

For the transcriptome we employ DESeq2 to generate normalized gene counts and estimate dispersion per gene. Expression and dispersion for the 500 lowest expressed genes (out of ~20,000 genes) are saved as proxy to generate noise of the transcriptome dataset.

```
# Make DESeq2 object
check <- all(rownames(metadata) == colnames(Transcriptome_raw))

if (check) {
  dds <- DESeq2::DESeqDataSetFromMatrix(
    countData = Transcriptome_raw,
    colData = metadata,
    design = ~ Patient+Stage
  )

  # Drop genes which have almost no expression across the dataset.
  smallestGroupSize <- 5
  keep <- rowSums(DESeq2::counts(dds) >= 10) >= smallestGroupSize
  dds <- dds[keep,]

  dds <- DESeq2::estimateSizeFactors(dds)
  dds <- DESeq2::estimateDispersions(dds)
}

# Calculate dispersion and mean expression estimate for the negative binomial distribution
# from the 500 lowest expressed genes as they likely reflect the technical noise in our dataset.

## Extract 500 lowest expressed genes
```

```
lowest_expressed_genes_mean <-DESeq2::counts(dds, normalized = TRUE) %>%
  rowMeans() %>%
  sort() %>%
  .[1:500]

## Let's check if the selected genes are present in the MOFA model as this may introduce bias
check <- all(lowest_expressed_genes_mean %in% rownames(MOFAobject@data$Transcriptome$group1))
if (!check) {
  cat("None of the selected 500 genes to calculate estimates for negative binomial noise \n are present
}

## Get the dispersion of the 500 lowest expressed genes
Transcriptome_dispersion <- DESeq2::dispersions(dds)
names(Transcriptome_dispersion) <-  DESeq2::counts(dds, normalized = TRUE) %>% rownames()
lowest_expressed_genes_disp <- Transcriptome_dispersion[names(lowest_expressed_genes_mean)]
```

2.3.2 Microbiome

For the microbiome we use the R package wrench to generate normalized microbe abundance counts (Kumar et al. (2018)). Dispersion is estimated by dividing each microbe's variance by it's mean. Abundance and dispersion for the 50 lowest abundant microbes (out of ~20,000 microbes) are saved as proxy to generate noise of the microbiome dataset.

```
### Filtered out microbes with very low abundance across the dataset
min_abundance <- 800 # The minimum abundance for each feature in counts
min_prevalence <- 0.03 # The minimum percent of samples for which a feature is detected at minimum abun
n_samples <- ncol(microbiome_raw)

microbiome_raw <- microbiome_raw[
  rowSums(microbiome_raw >  min_abundance) > n_samples * min_prevalence, drop = FALSE,
]

# Use Wrench to estimate the normalization factors
norm_factors <- Wrench::wrench(as.matrix(microbiome_raw), as.factor(metadata$stage)) %>%
  .[["nf"]]
microbiome_norm <- sweep(microbiome_raw, 2, norm_factors, FUN = '/')

# Calculate dispersion and mean abundance estimate for the negative binomial distribution
# from the 50 lowest abundant microbes as they likely reflect the technical noise in our dataset.

## Extract 50 lowest abundant microbes
lowest_abundant_microbes_mean_wrench <- microbiome_norm %>%
  rowMeans() %>%
  sort() %>%
  .[1:50]

# Let's check if the selected genes are present in the MOFA model as this may introduce bias
check <- all(
  names(lowest_abundant_microbes_mean_wrench) %in% rownames(MOFAobject@data$Microbiome$group1)
)
if (!check) {
  cat("None of the selected 50 microbes to calculate estimates for negative binomial noise are present
}
```

```r
# Get the dispersion of the 50 lowest abundant microbes
lowest_abundant_microbes_var_wrench <- matrixStats::rowVars(as.matrix(microbiome_norm))
names(lowest_abundant_microbes_var_wrench) <- rownames(microbiome_norm)
lowest_abundant_microbes_var_wrench <- lowest_abundant_microbes_var_wrench[
  names(lowest_abundant_microbes_mean_wrench)
]

# Compute dispersion as variance-to-mean ratio
lowest_abundant_microbes_disp_wrench <- lowest_abundant_microbes_var_wrench /
                                        lowest_abundant_microbes_mean_wrench
```

2.4 Representation of Datasets with added Noise in Lower Space (Dimensionality Reduction)

We will now check how the transcriptome and the microbiome data distributes in a lower dimensional space after adding different levels of noise and compare that to the original data without noise. We will do this for the entire datasets, as well as only for the features selected for the MOFA model in the manuscript.

2.4.1 Transcriptome

For the transcriptome we rlog transform the dataset and visualize using Principal Component Analysis (PCA) colored to stage.

```r
# Let's check how the PCA looks like with different levels of noise introduced
## First generate the PCA without noise
pca_no_noise <- list()
dds <- DESeq2::DESeqDataSetFromMatrix(
  countData = Transcriptome_raw,
  colData = metadata,
  design = ~ Patient+Stage
)

dds <- DESeq2::DESeq(dds)

## rlog transform
rld <- rlog(dds)

## generate plots
pca_no_noise[["full_dataset"]] <- plotPCA(rld, intgroup = "Stage") +
  theme_bw() +
  ggtitle("Full Data set w/o noise") +
  guides(color = guide_legend(title = "Stage"))

pca_no_noise[["5000_genes_in_MOFA"]] <- plotPCA(
  rld[rownames(MOFAobject@data$Transcriptome$group1), ],
  intgroup = "Stage"
) +
  theme_bw() +
  ggtitle("5000 genes in MOFA w/o noise") +
  guides(color = guide_legend(title = "Stage"))
```

```
# PCA for different dispersion levels
dispersion_levels <- c(0.01, 0.1, 0.5, 1, 1.33, 1.66, 2, 3, 4, 5, 6, 7, 8, 9, 10)
pca_noise <- list()
for (level in dispersion_levels) {
  cat("Testing dispersion level of:", level, "\n")

  # Add random noise to the raw count data
  dds_noise <- Transcriptome_NoSkin2_raw_counts
  for (i in 1:nrow(dds_noise)) {
    random_nb_noise <- generate_nb_noise(
      mean =  mean(lowest_expressed_genes_mean),
      dispersion = mean(lowest_expressed_genes_disp),
      disp_leverage = level,
      n_samples = ncol(dds_noise)
    )
    dds_noise[i, ] <- dds_noise[i, ] + random_nb_noise
  }

  # Create DESeq2 Object of raw counts with added random noise
  dds_noise <- DESeq2::DESeqDataSetFromMatrix(
    countData = dds_noise,
    colData = metadata,
    design = ~ Patient+Stage
  )
  dds_noise <- DESeq2::estimateSizeFactors(dds_noise)
  dds_noise <- DESeq2::estimateDispersions(dds_noise)

  # rlog transform data
  rld_noise <- rlog(dds_noise)

  # Generate plots
  x <- paste0("dispersion_lvl_", level)
  pca_noise[[x]][["full_dataset"]] <- plotPCA(rld_noise, intgroup = "Stage") +
    theme_bw() +
    ggtitle(paste0("Full Dataset with noise level: ", level)) +
    guides(color = guide_legend(title = "Stage"))

  pca_noise[[x]][["genes_in_MOFA"]] <- plotPCA(
    rld_noise[rownames(MOFAobject@data$Transcriptome$group1), ],
    intgroup = "Stage"
  ) +
    theme_bw() +
    ggtitle(paste0("5000 genes in MOFA with noise level: ", level)) +
    guides(color = guide_legend(title = "Stage"))
}

# Plot the results
ggarrange(
  pca_no_noise$full_dataset,
  pca_no_noise[["5000_genes_in_MOFA"]],
  pca_noise$dispersion_lvl_0.01$full_dataset,
  pca_noise$dispersion_lvl_0.01$genes_in_MOFA,
  pca_noise$dispersion_lvl_0.1$full_dataset,
```

```r
  pca_noise$dispersion_lvl_0.1$genes_in_MOFA,
  pca_noise$dispersion_lvl_0.5$full_dataset,
  pca_noise$dispersion_lvl_0.5$genes_in_MOFA,
  pca_noise$dispersion_lvl_1$full_dataset,
  pca_noise$dispersion_lvl_1$genes_in_MOFA,
  pca_noise$dispersion_lvl_1.33$full_dataset,
  pca_noise$dispersion_lvl_1.33$genes_in_MOFA,
  pca_noise$dispersion_lvl_1.66$full_dataset,
  pca_noise$dispersion_lvl_1.66$genes_in_MOFA,
  pca_noise$dispersion_lvl_2$full_dataset,
  pca_noise$dispersion_lvl_2$genes_in_MOFA,
  pca_noise$dispersion_lvl_3$full_dataset,
  pca_noise$dispersion_lvl_3$genes_in_MOFA,
  pca_noise$dispersion_lvl_4$full_dataset,
  pca_noise$dispersion_lvl_4$genes_in_MOFA,
  pca_noise$dispersion_lvl_5$full_dataset,
  pca_noise$dispersion_lvl_5$genes_in_MOFA,
  pca_noise$dispersion_lvl_6$full_dataset,
  pca_noise$dispersion_lvl_6$genes_in_MOFA,
  pca_noise$dispersion_lvl_7$full_dataset,
  pca_noise$dispersion_lvl_7$genes_in_MOFA,
  pca_noise$dispersion_lvl_8$full_dataset,
  pca_noise$dispersion_lvl_8$genes_in_MOFA,
  pca_noise$dispersion_lvl_9$full_dataset,
  pca_noise$dispersion_lvl_9$genes_in_MOFA,
  pca_noise$dispersion_lvl_10$full_dataset,
  pca_noise$dispersion_lvl_10$genes_in_MOFA,
  common.legend = TRUE,
  ncol = 2,
  nrow = 16
)
```

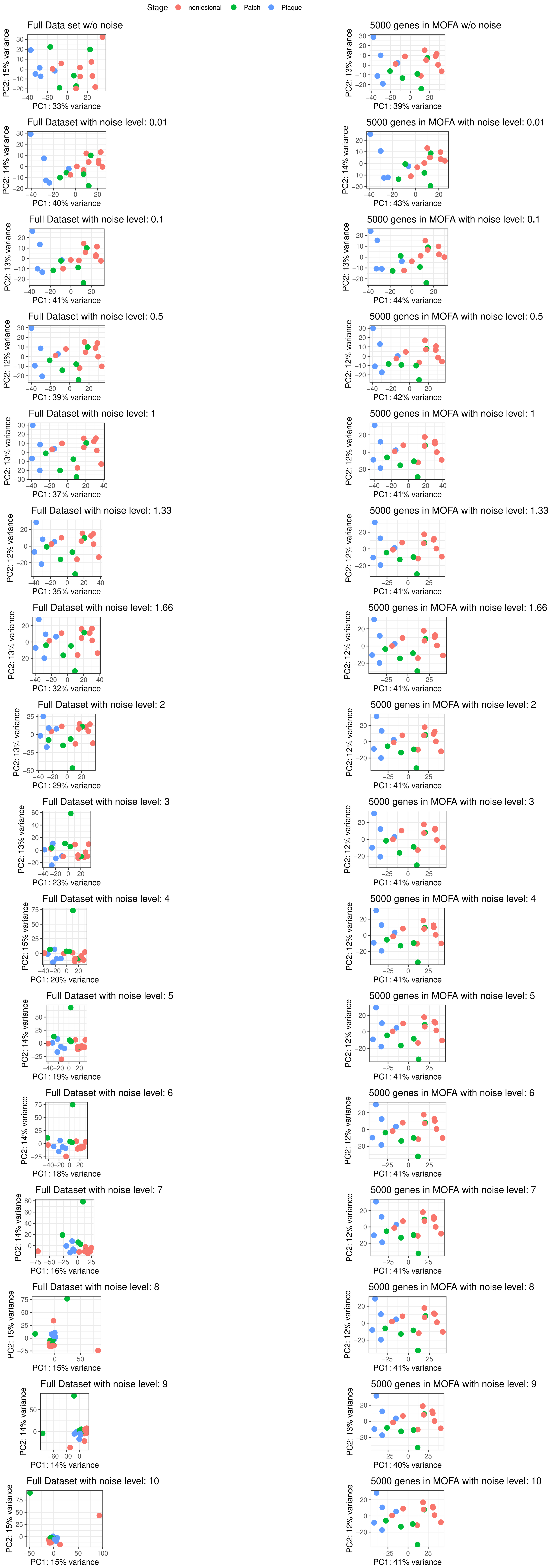

Stage nonlesional Patch Plaque
Full Data set w/o noise
PC1: 33% variance
PC2: 15% variance
5000 genes in MOFA w/o noise
PC1: 39% variance
PC2: 13% variance
Full Dataset with noise level: 0.01
PC1: 40% variance
PC2: 14% variance
5000 genes in MOFA with noise level: 0.01
PC1: 43% variance
PC2: 14% variance
Full Dataset with noise level: 0.1
PC1: 41% variance
PC2: 13% variance
5000 genes in MOFA with noise level: 0.1
PC1: 44% variance
PC2: 13% variance
Full Dataset with noise level: 0.5
PC1: 39% variance
PC2: 12% variance
5000 genes in MOFA with noise level: 0.5
PC1: 42% variance
PC2: 12% variance
Full Dataset with noise level: 1
PC1: 37% variance
PC2: 13% variance
5000 genes in MOFA with noise level: 1
PC1: 41% variance
PC2: 12% variance
Full Dataset with noise level: 1.33
PC1: 35% variance
PC2: 12% variance
5000 genes in MOFA with noise level: 1.33
PC1: 41% variance
PC2: 12% variance
Full Dataset with noise level: 1.66
PC1: 32% variance
PC2: 13% variance
5000 genes in MOFA with noise level: 1.66
PC1: 41% variance
PC2: 12% variance
Full Dataset with noise level: 2
PC1: 29% variance
PC2: 13% variance
5000 genes in MOFA with noise level: 2
PC1: 41% variance
PC2: 12% variance
Full Dataset with noise level: 3
PC1: 23% variance
PC2: 13% variance
5000 genes in MOFA with noise level: 3
PC1: 41% variance
PC2: 12% variance
Full Dataset with noise level: 4
PC1: 20% variance
PC2: 15% variance
5000 genes in MOFA with noise level: 4
PC1: 41% variance
PC2: 12% variance
Full Dataset with noise level: 5
PC1: 19% variance
PC2: 14% variance
5000 genes in MOFA with noise level: 5
PC1: 41% variance
PC2: 12% variance
Full Dataset with noise level: 6
PC1: 18% variance
PC2: 14% variance
5000 genes in MOFA with noise level: 6
PC1: 41% variance
PC2: 12% variance
Full Dataset with noise level: 7
PC1: 16% variance
PC2: 14% variance
5000 genes in MOFA with noise level: 7
PC1: 41% variance
PC2: 12% variance
Full Dataset with noise level: 8
PC1: 15% variance
PC2: 15% variance
5000 genes in MOFA with noise level: 8
PC1: 41% variance
PC2: 12% variance
Full Dataset with noise level: 9
PC1: 14% variance
PC2: 14% variance
5000 genes in MOFA with noise level: 9
PC1: 40% variance
PC2: 13% variance
Full Dataset with noise level: 10
PC1: 15% variance
PC2: 15% variance
5000 genes in MOFA with noise level: 10
PC1: 41% variance
PC2: 12% variance

2.4.2 Microbiome

For the microbiome we log2 transform the dataset and visualize with Principal Coordinate Analysis (PCoA) used on a bray-curtis distance matrix colored to stage.

Let's first define a function to easily perform PCoA:

```
# Define Function to generate PCoA based on bray-curtis distance matrix
do_pcoa <- function(mat, distance_metric = "bray", ...) {
  distance_calc <- vegan::vegdist(mat, method = distance_metric)
  pcoa <- cmdscale(distance_calc, eig = TRUE, add = TRUE, ...)
  perc_explained <- (100 * pcoa$eig / sum(pcoa$eig)) %>%
    round(digits = 2) %>%
    format(nsmall = 2, trim = TRUE)
  colnames(pcoa$points) <- c("pcoa1", "pcoa2")

  return(list(
    PCOA = pcoa,
    explained_variance = perc_explained
  ))
}
```

Now, let's use `do_pcoa()` to generate PCoA scatter plots for the MOFA models with and without noise:

```
# First generate the PCoA without noise
## log2 transform with pseudocount 1
microbiome_norm_log2 <- log2(microbiome_norm + 1)

pcoa_no_noise_microbiome <- list()
# Full Dataset
pcoa <- do_pcoa(t(microbiome_norm_log2), distance_metric = "bray")

pcoa_no_noise_microbiome[["full_dataset"]] <- pcoa$PCOA$points %>%
  as_tibble(rownames = "sample") %>%
  inner_join(metadata %>% mutate(sample = str_remove_all(sample, "^[^_]*_"))) %>%
  ggplot(aes(x = pcoa1, y = pcoa2, color = Stage)) +
  geom_point(size = 3) +
  labs(
    x = glue::glue("PCo1 ({pcoa$explained_variance[1]}%)"),
    y = glue::glue("PCo2 ({pcoa$explained_variance[2]}%)")
  ) +
  theme_bw() +
  ggtitle("Full Data set w/o noise")

# Only with Features included in MOFA model
pcoa <- do_pcoa(
  t(microbiome_norm_log2[rownames(MOFAobject@data$Microbiome$group1), ]),
  distance_metric = "bray"
)

pcoa_no_noise_microbiome[["50_microbes_in_MOFA"]] <- pcoa$PCOA$points %>%
  as_tibble(rownames = "sample") %>%
  inner_join(metadata %>% mutate(sample = str_remove_all(sample, "^[^_]*_"))) %>%
  ggplot(aes(x = pcoa1, y = pcoa2, color = Stage)) +
```

```r
  geom_point(size = 3) +
  labs(
    x = glue::glue("PCo1 ({pcoa$explained_variance[1]}%)"),
    y = glue::glue("PCo2 ({pcoa$explained_variance[2]}%)")
  ) +
  theme_bw() +
  ggtitle("50 microbes in MOFA w/o noise")

# PCOA for different dispersion levels
dispersion_levels <- c(0.01, 0.1, 0.5, 1, 1.33, 1.66, 2, 3, 4, 5, 6, 7, 8, 9, 10)
pcao_noise <- list()
for (level in dispersion_levels) {
  cat("Testing dispersion level of:", level, "\n")

  # Add random noise to the raw count data
  microbiome_noise <- microbiome_raw
  for (i in 1:nrow(microbiome_noise)) {
    random_nb_noise <- generate_nb_noise(
      mean =  mean(lowest_abundant_microbes_mean_wrench),
      dispersion = mean(lowest_abundant_microbes_disp_wrench),
      disp_leverage = level,
      n_samples = ncol(microbiome_noise)
    )
    microbiome_noise[i, ] <- microbiome_noise[i, ] + random_nb_noise
  }

  # Use Wrench to estimate the normalization factors
  norm_factors <- Wrench::wrench(as.matrix(microbiome_noise), as.factor(metadata$stage)) %>%
    .[["nf"]]
  microbiome_noise <- sweep(microbiome_noise, 2, norm_factors, FUN = '/')


  # log2 transform with pseudocount 1
  microbiome_noise <- log2(microbiome_noise + 1)

  # Generate plots
  x <- paste0("dispersion_lvl_", level)

  ## Full Dataset
  pcoa <- do_pcoa(t(microbiome_noise), distance_metric = "bray")
  pcao_noise[[x]][["full_dataset"]] <- pcoa$PCOA$points %>%
    as_tibble(rownames = "sample") %>%
    inner_join(metadata %>% mutate(sample = str_remove_all(sample, "^[^_]*_"))) %>%
    ggplot(aes(x = pcoa1, y = pcoa2, color = Stage)) +
    geom_point(size = 3) +
    labs(
      x = glue::glue("PCo1 ({pcoa$explained_variance[1]}%)"),
      y = glue::glue("PCo2 ({pcoa$explained_variance[2]}%)")
    ) +
    theme_bw() +
    ggtitle(paste0("Full Dataset with noise level: ", level))

  ## Only with the Microbes used in MOFA model
```

```r
  pcoa <- do_pcoa(
    t(microbiome_noise[rownames(MOFAobject@data$Microbiome$group1), ]),
    distance_metric = "bray"
  )

  pcao_noise[[x]][["microbes_in_MOFA"]] <- pcoa$PCOA$points %>%
    as_tibble(rownames = "sample") %>%
    inner_join(metadata %>% mutate(sample = str_remove_all(sample, "^[^_]*_"))) %>%
    ggplot(aes(x = pcoa1, y = pcoa2, color = Stage)) +
    geom_point(size = 3) +
    labs(
      x = glue::glue("PCo1 ({pcoa$explained_variance[1]}%)"),
      y = glue::glue("PCo2 ({pcoa$explained_variance[2]}%)")
    ) +
    theme_bw() +
    ggtitle(paste0("50 microbes in MOFA with noise level: ", level))
}

# Plot the results
ggarrange(
  pcoa_no_noise_microbiome$full_dataset,
  pcoa_no_noise_microbiome[["50_microbes_in_MOFA"]],
  pcao_noise$dispersion_lvl_0.01$full_dataset,
  pcao_noise$dispersion_lvl_0.01$microbes_in_MOFA,
  pcao_noise$dispersion_lvl_0.1$full_dataset,
  pcao_noise$dispersion_lvl_0.1$microbes_in_MOFA,
  pcao_noise$dispersion_lvl_0.5$full_dataset,
  pcao_noise$dispersion_lvl_0.5$microbes_in_MOFA,
  pcao_noise$dispersion_lvl_1$full_dataset,
  pcao_noise$dispersion_lvl_1$microbes_in_MOFA,
  pcao_noise$dispersion_lvl_1.33$full_dataset,
  pcao_noise$dispersion_lvl_1.33$microbes_in_MOFA,
  pcao_noise$dispersion_lvl_1.66$full_dataset,
  pcao_noise$dispersion_lvl_1.66$microbes_in_MOFA,
  pcao_noise$dispersion_lvl_2$full_dataset,
  pcao_noise$dispersion_lvl_2$microbes_in_MOFA,
  pcao_noise$dispersion_lvl_3$full_dataset,
  pcao_noise$dispersion_lvl_3$microbes_in_MOFA,
  pcao_noise$dispersion_lvl_4$full_dataset,
  pcao_noise$dispersion_lvl_4$microbes_in_MOFA,
  pcao_noise$dispersion_lvl_5$full_dataset,
  pcao_noise$dispersion_lvl_5$microbes_in_MOFA,
  pcao_noise$dispersion_lvl_6$full_dataset,
  pcao_noise$dispersion_lvl_6$microbes_in_MOFA,
  pcao_noise$dispersion_lvl_7$full_dataset,
  pcao_noise$dispersion_lvl_7$microbes_in_MOFA,
  pcao_noise$dispersion_lvl_8$full_dataset,
  pcao_noise$dispersion_lvl_8$microbes_in_MOFA,
  pcao_noise$dispersion_lvl_9$full_dataset,
  pcao_noise$dispersion_lvl_9$microbes_in_MOFA,
  pcao_noise$dispersion_lvl_10$full_dataset,
  pcao_noise$dispersion_lvl_10$microbes_in_MOFA,
  common.legend = TRUE,
```

```
  ncol = 2,
  nrow = 16
)
```

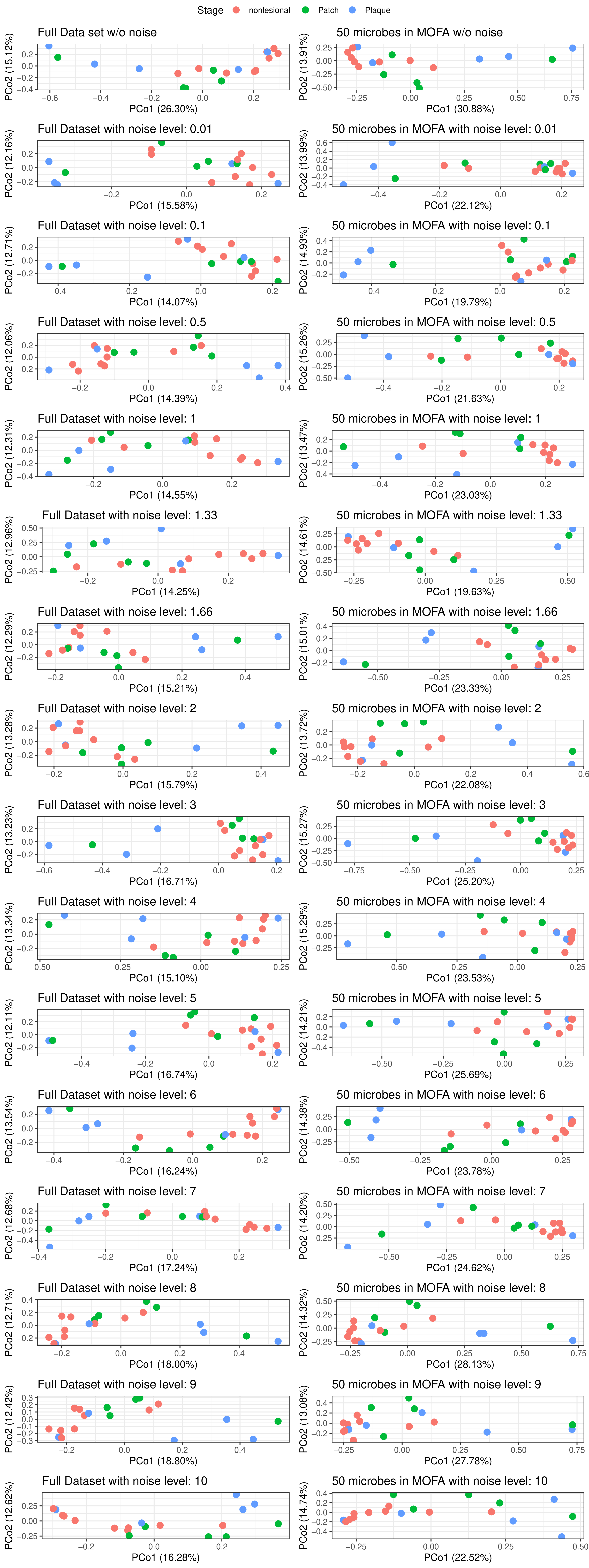

Stage
nonlesional
Patch
Plaque
Full Data set w/o noise
PCo1 (26.30%)
PCo2 (15.12%)
50 microbes in MOFA w/o noise
PCo1 (30.88%)
PCo2 (13.91%)
Full Dataset with noise level: 0.01
PCo1 (15.58%)
PCo2 (12.16%)
50 microbes in MOFA with noise level: 0.01
PCo1 (22.12%)
PCo2 (13.99%)
Full Dataset with noise level: 0.1
PCo1 (14.07%)
PCo2 (12.71%)
50 microbes in MOFA with noise level: 0.1
PCo1 (19.79%)
PCo2 (14.93%)
Full Dataset with noise level: 0.5
PCo1 (14.39%)
PCo2 (12.06%)
50 microbes in MOFA with noise level: 0.5
PCo1 (21.63%)
PCo2 (15.26%)
Full Dataset with noise level: 1
PCo1 (14.55%)
PCo2 (12.31%)
50 microbes in MOFA with noise level: 1
PCo1 (23.03%)
PCo2 (13.47%)
Full Dataset with noise level: 1.33
PCo1 (14.25%)
PCo2 (12.96%)
50 microbes in MOFA with noise level: 1.33
PCo1 (19.63%)
PCo2 (14.61%)
Full Dataset with noise level: 1.66
PCo1 (15.21%)
PCo2 (12.29%)
50 microbes in MOFA with noise level: 1.66
PCo1 (23.33%)
PCo2 (15.01%)
Full Dataset with noise level: 2
PCo1 (15.79%)
PCo2 (13.28%)
50 microbes in MOFA with noise level: 2
PCo1 (22.08%)
PCo2 (13.72%)
Full Dataset with noise level: 3
PCo1 (16.71%)
PCo2 (13.23%)
50 microbes in MOFA with noise level: 3
PCo1 (25.20%)
PCo2 (15.27%)
Full Dataset with noise level: 4
PCo1 (15.10%)
PCo2 (13.34%)
50 microbes in MOFA with noise level: 4
PCo1 (23.53%)
PCo2 (15.29%)
Full Dataset with noise level: 5
PCo1 (16.74%)
PCo2 (12.11%)
50 microbes in MOFA with noise level: 5
PCo1 (25.69%)
PCo2 (14.21%)
Full Dataset with noise level: 6
PCo1 (16.24%)
PCo2 (13.54%)
50 microbes in MOFA with noise level: 6
PCo1 (23.78%)
PCo2 (14.38%)
Full Dataset with noise level: 7
PCo1 (17.24%)
PCo2 (12.68%)
50 microbes in MOFA with noise level: 7
PCo1 (24.62%)
PCo2 (14.20%)
Full Dataset with noise level: 8
PCo1 (18.00%)
PCo2 (12.71%)
50 microbes in MOFA with noise level: 8
PCo1 (28.13%)
PCo2 (14.32%)
Full Dataset with noise level: 9
PCo1 (18.80%)
PCo2 (12.42%)
50 microbes in MOFA with noise level: 9
PCo1 (27.78%)
PCo2 (13.08%)
Full Dataset with noise level: 10
PCo1 (16.28%)
PCo2 (12.62%)
50 microbes in MOFA with noise level: 10
PCo1 (22.52%)
PCo2 (14.74%)

2.5 Interpretation of Dimensionality Reduction Plots

The transcriptome begins to distort beginning from noise level 2 in the full dataset. Focused only on the genes included in the manuscript's MOFA model, the PCA is very stable across all noise levels with similar degree of explained variance on principal components one and two. The microbiome full dataset begins to distort starting from noise level 1. The PCA of the 50 microbes included in the manuscript's MOFA model is less stable across noise levels than the transcriptome equivalent. Yet, the structure of the initial data without noise is more or less preseverd throughout all noise levels. The proportion of explained variance on principal coordinate 1 drops from ~31% to ~23%.

This suggests that the selected features of both views for the manuscript's MOFA model represent the inherent structure of the Mycosis fungoides patient population are robust against technical noise.

2.6 Run the MOFA model with the perturbed data

The following code runs MOFA with the same options like the manuscript's MOFA model for the 15 different noise levels: `c(0.01, 0.1, 0.5, 1, 1.33, 1.66, 2, 3, 4, 5, 6, 7, 8, 9, 10)`. Each noise level is repeated for 10 iterations to assess whether MOFA robustly generates the same results in every noise level.

```
# Set options for running MOFA
data_options <- list(
  scale_views = TRUE,
  scale_groups = FALSE,
  center_groups = TRUE,
  use_float32 = FALSE,
  views = c("Transcriptome", "Microbiome"),
  groups = "group1"
)

model_options <- list(
  likelihoods = c(Transcriptome = "gaussian", Microbiome = "gaussian"),
  num_factors = 8,
  spikeslab_factors = FALSE,
  spikeslab_weights = TRUE,
  ard_factors = FALSE,
  ard_weights = TRUE
)

training_options <- list(
  maxiter = 2000,
  convergence_mode = "fast",
  drop_factor_threshold = -1,
  verbose = FALSE,
  startELBO = 1,
  freqELBO = 5,
  stochastic = FALSE,
  gpu_mode = FALSE,
  seed = 42,
  outfile = NULL,
  weight_views = FALSE,
  save_interrupted = FALSE
)

# Parameters for stability testing
```

```r
n_iter <- 10 # Iterations per dispersion level
dispersion_levels <- c(0.01, 0.1, 0.5, 1, 1.33, 1.66, 2, 3, 4, 5, 6, 7, 8, 9, 10)

# Set list for saving
MOFA_noise_out <- list()

for (level in dispersion_levels) {
  cat("Testing dispersion level of:", level, "\n")
  x <- paste0("dispersion_lvl_", level)

  for (i in 1:n_iter) {
    cat("Running Iteration: ", i, "of 10\n")
    y <- paste0("Iteration:", i)

    ####################################
    ## Perturb raw transcriptome data ##
    ####################################

    # Add random noise to the raw count data
    dds_noise <- Transcriptome_raw
    for (i in 1:nrow(dds_noise)) {
      random_nb_noise <- generate_nb_noise(
        mean =  mean(lowest_expressed_genes_mean),
        dispersion = mean(lowest_expressed_genes_disp),
        disp_leverage = level,
        n_samples = ncol(dds_noise)
      )
      dds_noise[i, ] <- dds_noise[i, ] + random_nb_noise
    }

    # Create DESeq2 Object of raw counts with added random noise
    dds_noise <- DESeq2::DESeqDataSetFromMatrix(
      countData = dds_noise,
      colData = metadata,
      design = ~ Patient+Stage
    )
    dds_noise <- DESeq2::estimateSizeFactors(dds_noise)
    dds_noise <- DESeq2::estimateDispersions(dds_noise)

    # rlog transform data
    rld_noise <- rlog(dds_noise)

    # Extract rlog transformed counts of the genes used in the original MOFA model
    transcriptome_noise <- rld_noise %>%
      assay %>%
      .[rownames(MOFAobject@data$Transcriptome$group1), ]


    #################################
    ## Perturb raw microbiome data ##
    #################################

    # Add random noise to the raw count data
```

```r
  microbiome_noise <- microbiome_raw
  for (i in 1:nrow(microbiome_noise)) {
    random_nb_noise <- generate_nb_noise(
      mean =  mean(lowest_abundant_microbes_mean_wrench),
      dispersion = mean(lowest_abundant_microbes_disp_wrench),
      disp_leverage = level,
      n_samples = ncol(microbiome_noise)
    )
    microbiome_noise[i, ] <- microbiome_noise[i, ] + random_nb_noise
  }

  # Use Wrench to estimate the normalization factors
  norm_factors <- Wrench::wrench(as.matrix(microbiome_noise), as.factor(metadata$stage)) %>%
    .[["nf"]]
  microbiome_noise <- sweep(microbiome_noise, 2, norm_factors, FUN = '/')


  # log2 transform with pseudocount 1
  microbiome_noise <- log2(microbiome_noise + 1)

  # Retain microbes used in the original MOFA model
  microbiome_noise <- microbiome_noise %>%
    as.matrix() %>%
    .[rownames(MOFAobject@data$Microbiome$group1), ]

  ##################################
  ## Run MOFA with perturbed data ##
  ##################################

  # check if colnames of transcriptome and microbiome are exactly similar
  check <- all(colnames(transcriptome_noise) == colnames(microbiome_noise))
  if (!check) {stop("Colnames of Microbiome and Transcriptome are not similar!")}

  # Set MOFA object with perturbed data
  MOFA_noise <- create_mofa(list(
    Transcriptome = transcriptome_noise,
    Microbiome = microbiome_noise
  ))

  # Prepare MOFA object
  MOFA_noise <- prepare_mofa(
    object = MOFA_noise,
    data_options = data_options,
    model_options = model_options,
    training_options = training_options
  )

  # Run MOFA
  MOFA_noise <- run_mofa(MOFA_noise)

  # Save in list
  MOFA_noise_out[[x]][[y]] <- MOFA_noise
}
```

```
}
```

2.7 Analysis of MOFA models with perturbed data

We now inspect the MOFA models of the 15 different noise levels against the original MOFA model of the manuscript.

2.7.1 Comparison of explained variance

Cumulative explained variance per view

First, extract and inspect the cumulative variance explained per **view**

```
# Extract variance explained per view
var_expl_transcriptome <- list()
var_expl_microbiome <- list()

## Extract all variance explained values for factor
for (level in names(MOFA_noise_out)) {
  for (iteration in names(MOFA_noise_out[[level]])) {
    var_expl_transcriptome[[level]][[iteration]] <-
      MOFA_noise_out[[level]][[iteration]]@cache$variance_explained$r2_total$group1[1]
    var_expl_microbiome[[level]][[iteration]] <-
      MOFA_noise_out[[level]][[iteration]]@cache$variance_explained$r2_total$group1[2]
  }
}
var_expl_transcriptome <- map_dfr(
  var_expl_transcriptome,
  ~enframe(., name = "Iteration", value = "Variance_Explained_Transcriptome"),
  .id = "Noise_Level"
) %>%
  tidyr::unnest(cols = c(Variance_Explained_Transcriptome))

var_expl_microbiome <- map_dfr(
  var_expl_microbiome,
  ~enframe(., name = "Iteration", value = "Variance_Explained_Microbiome"),
  .id = "Noise_Level"
) %>%
  tidyr::unnest(cols = c(Variance_Explained_Microbiome))

## Add the values from the original MOFA model
var_expl_transcriptome <- bind_rows(
  tibble(
    Noise_Level = "model_used_in_manuscript",
    Iteration = "model_used_in_manuscript",
    Variance_Explained_Transcriptome = MOFAobject@cache$variance_explained$r2_total$group1[1]
  ),
  var_expl_transcriptome
)

var_expl_microbiome <-  bind_rows(
  tibble(
```

```
    Noise_Level = "model_used_in_manuscript",
    Iteration = "model_used_in_manuscript",
    Variance_Explained_Microbiome = MOFAobject@cache$variance_explained$r2_total$group1[2]
  ),
  var_expl_microbiome
)

## Combine into single tibble
var_explained_per_view <- inner_join(var_expl_transcriptome, var_expl_microbiome)
```

Now, we test if the variance explained per view is statistically different from the original MOFA model of the manuscript.

```
# Test if the variance explained in the perturbed MOFA models are statistically different
# from the original MOFA model
variance_explained__per_view_comparison <- list()
for (omic in c("Transcriptome", "Microbiome")) {
  cat("Omic:", omic, "\n")
  for (level in unique(var_explained_per_view$Noise_Level)[-1]) {

    # Filter to the data you want to test
    to_test <- var_explained_per_view %>%
      dplyr::filter(Noise_Level == level) %>%
      dplyr::select(Noise_Level, Iteration, contains(omic)) %>%
      rename_with(~ "Variance_Explained", .cols = contains(omic))

    # Check for normal distribution
    is_normal_distributed <- shapiro.test(to_test$Variance_Explained) %>% broom::tidy()
    if (is_normal_distributed$p.value <= 0.05) {
      cat("Normal Distributed, employing one-sample t-test.\n")

      variance_explained__per_view_comparison[[omic]][[level]] <- t.test(
        to_test$Variance_Explained,
        alternative = "two.sided",
        mu = var_explained_per_view %>%
          dplyr::filter(Noise_Level == "model_used_in_manuscript") %>%
          rename_with(~ "Variance_Explained", .cols = contains(omic)) %>%
          pull(Variance_Explained)
      ) %>%
        broom::tidy() %>%
        dplyr::select(p.value, method, alternative)


    } else {
      cat("No normal distribution, employing one-sample  Wilcoxon test.\n")

      variance_explained__per_view_comparison[[omic]][[level]] <- wilcox.test(
        to_test$Variance_Explained,
        alternative = "two.sided",
        mu = var_explained_per_view %>%
          dplyr::filter(Noise_Level == "model_used_in_manuscript") %>%
          rename_with(~ "Variance_Explained", .cols = contains(omic)) %>%
          pull(Variance_Explained)
```

```r
      ) %>%
        broom::tidy() %>%
        dplyr::select(p.value, method, alternative)
    }

  }
}

# Combine into one tibble
variance_explained__per_view_comparison <- map_dfr(
  variance_explained__per_view_comparison,
  ~ map_dfr(.x, ~ .x, .id = "Noise Level"),
  .id = "View"
)
```

We now plot violin-plots of the variance explained per view and add significance asterisks based on our prior calculations.

```r
## Add asterics annotation
variance_explained__per_view_comparison <- variance_explained__per_view_comparison %>%
  mutate(significance = case_when(
    p.value < 0.001 ~ "***",
    p.value < 0.01 ~ "**",
    p.value < 0.05 ~ "*",
    TRUE ~ "ns"  # ns for non-significant
  ))

# Make violin plots
df <- var_explained_per_view %>%
  mutate(
    x_label = factor(
      case_when(
        Noise_Level == "model_used_in_manuscript" ~ "Original Model (no noise)",
        TRUE ~ str_remove(Noise_Level, ".*lvl_")
      ),
      levels = c("Original Model (no noise)", Noise_Level %>% unique() %>% str_remove(".*lvl_"))
    )
  )

var_expl_violin_transcriptome <- df %>%
  ggplot(aes(x = x_label, y = Variance_Explained_Transcriptome)) +
  geom_violin(drop = FALSE) +
  geom_point() +
  labs(x = element_blank(), y = "Variance Explained in Transcriptome") +
  ggsignif::geom_signif(
      comparisons = list(
        c("Original Model (no noise)", "0.01"),
        c("Original Model (no noise)", "0.1"),
        c("Original Model (no noise)", "0.5"),
        c("Original Model (no noise)", "1"),
        c("Original Model (no noise)", "1.33"),
        c("Original Model (no noise)", "1.66"),
        c("Original Model (no noise)", "2"),
```

```
        c("Original Model (no noise)", "3"),
        c("Original Model (no noise)", "4"),
        c("Original Model (no noise)", "5"),
        c("Original Model (no noise)", "6"),
        c("Original Model (no noise)", "7"),
        c("Original Model (no noise)", "8"),
        c("Original Model (no noise)", "9"),
        c("Original Model (no noise)", "10")
    ),
    map_signif_level = TRUE,
    annotations = variance_explained__per_view_comparison %>%
      dplyr::filter(View == "Transcriptome") %>%
      pull(significance),
    y_position = seq(72, 79, by = 0.5)
  ) +
  theme_minimal() +
  theme(axis.text.x = element_text(angle = 90, hjust = 1, vjust = 0.2))

var_expl_violin_microbiome <- df %>%
  ggplot(aes(x = x_label, y = Variance_Explained_Microbiome)) +
  geom_violin(drop = FALSE) +
  geom_point() +
  labs(x = element_blank(), y = "Variance Explained in Microbiome") +
  ggsignif::geom_signif(
    comparisons = list(
      c("Original Model (no noise)", "0.01"),
      c("Original Model (no noise)", "0.1"),
      c("Original Model (no noise)", "0.5"),
      c("Original Model (no noise)", "1"),
      c("Original Model (no noise)", "1.33"),
      c("Original Model (no noise)", "1.66"),
      c("Original Model (no noise)", "2"),
      c("Original Model (no noise)", "3"),
      c("Original Model (no noise)", "4"),
      c("Original Model (no noise)", "5"),
      c("Original Model (no noise)", "6"),
      c("Original Model (no noise)", "7"),
      c("Original Model (no noise)", "8"),
      c("Original Model (no noise)", "9"),
      c("Original Model (no noise)", "10")
    ),
    map_signif_level = TRUE,
    annotations = variance_explained__per_view_comparison %>%
      dplyr::filter(View == "Microbiome") %>%
      pull(significance),
    y_position = seq(38, 45, by = 0.5)
  ) +
  theme_minimal() +
  theme(axis.text.x = element_text(angle = 90, hjust = 1, vjust = 0.2))

ggpubr::ggarrange(var_expl_violin_transcriptome, var_expl_violin_microbiome)
```

```
## Warning: Cannot compute density for groups with fewer than two datapoints.
```

```
## Cannot compute density for groups with fewer than two datapoints.
```

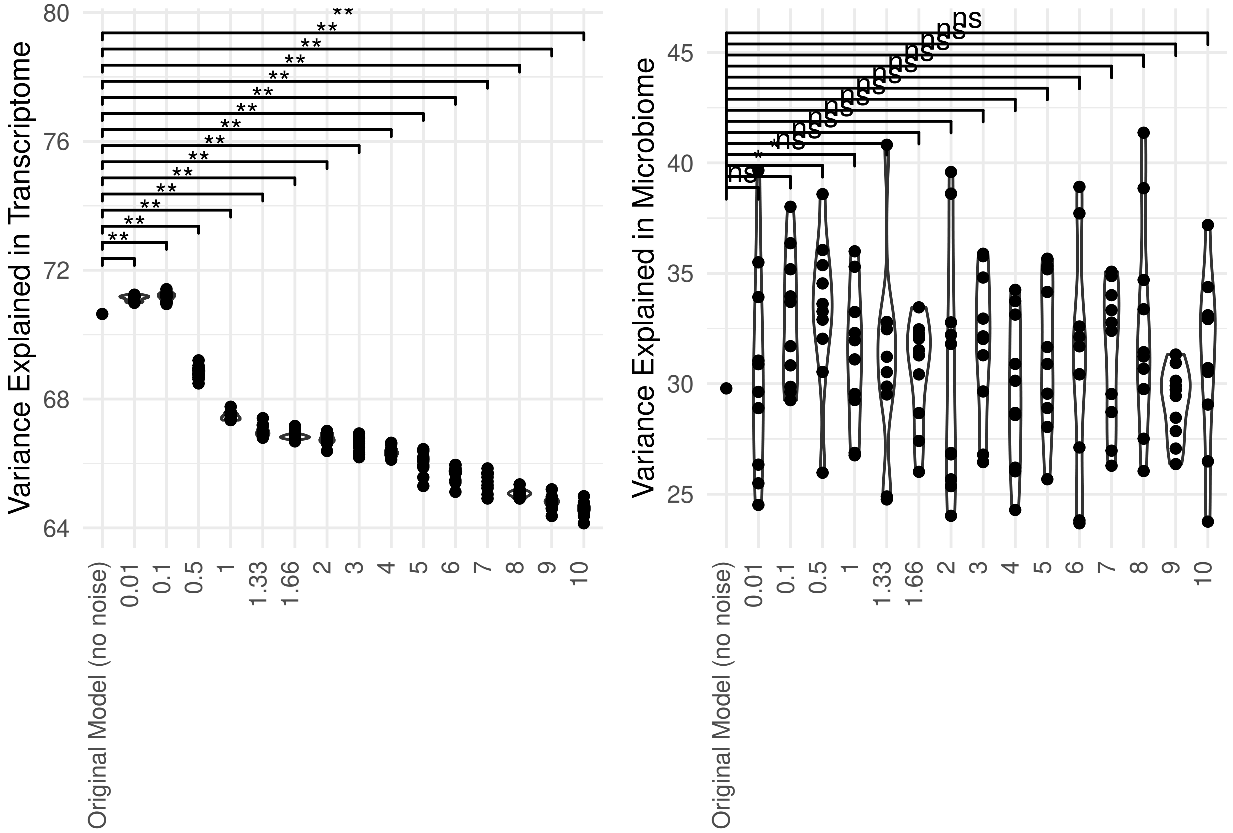

NOTE: p-values were not adjusted for multiple comparisons as this evaluation is of a rather hypothetical and artificial nature. If p-values would have been adjusted, all adjusted p-values would be > 0.05.

Interpretation

In the transcriptome, all tested noise levels are significantly different from the original MOFA model in the manuscript. Further, the iterations of the individual noise levels yielded comparable results. Interestingly, noise levels 0.01 and 0.1 are on a similar level like the original MOFA model, dropping at noise level 0.5 and hiher. In the microbiome, none of the noise levels is different from the original MOFA model in the manuscript, while the spread of the explained variance per iteration of individual noise levels is relatively high. The high spread is to be expected as it is considerably more complicated to generate meaningful negative bionomial noise for such a heterogenous and small dataset like the microbiome. In summary, it seems like that at least noise levels 0.01 and 0.05 explained variance akin to the original MOFA model.

Explained variance per factor and view

We next extract and inspect the variance explained broken down into **factor and view**

```r
# Extract variance explained per factor and view
var_expl_transcriptome <- list()
var_expl_microbiome <- list()

## Extract all variance explained values for factor
```

```r
for (level in names(MOFA_noise_out)) {
  for (iteration in names(MOFA_noise_out[[level]])) {
    var_expl_transcriptome[[level]][[iteration]] <-
      MOFA_noise_out[[level]][[iteration]]@cache$variance_explained$r2_per_factor$group1[, 1]
    var_expl_microbiome[[level]][[iteration]] <-
      MOFA_noise_out[[level]][[iteration]]@cache$variance_explained$r2_per_factor$group1[, 2]
  }
}
var_expl_transcriptome <- map_dfr(
  var_expl_transcriptome,
  ~map_dfr(., enframe, name = "Factor", value = "Variance_Explained_Transcriptome", .id = "Iteration"),
  .id = "Noise_Level"
)
var_expl_microbiome <- map_dfr(
  var_expl_microbiome,
  ~map_dfr(., enframe, name = "Factor", value = "Variance_Explained_Microbiome", .id = "Iteration"),
  .id = "Noise_Level"
)

## Add the values from the original MOFA model
var_expl_transcriptome <- bind_rows(
  tibble(
    Noise_Level = "model_used_in_manuscript",
    Iteration = "model_used_in_manuscript",
    enframe(
      MOFAobject@cache$variance_explained$r2_per_factor$group1[, 1],
      name = "Factor",
      value = "Variance_Explained_Transcriptome"
    )
  ),
  var_expl_transcriptome
)

var_expl_microbiome <-  bind_rows(
  tibble(
    Noise_Level = "model_used_in_manuscript",
    Iteration = "model_used_in_manuscript",
    enframe(
      MOFAobject@cache$variance_explained$r2_per_factor$group1[, 2],
      name = "Factor",
      value = "Variance_Explained_Microbiome"
    )
  ),
  var_expl_microbiome
)

## Combine into single tibble
var_explained_per_factor <- inner_join(var_expl_transcriptome, var_expl_microbiome)
```

We now plot heatmaps of the explained variance per factor, one heatmap for each view.

```r
annot_col <- var_explained_per_factor %>%
  mutate(Model = paste(Noise_Level, Iteration, sep = "_")) %>%
```

```r
  dplyr::select(Model, Noise_Level, Iteration) %>%
  distinct() %>%
  column_to_rownames("Model")

ann_colors <- list(
  Noise_Level = c( "black", "#33FF57", "#3357FF", "#F1C40F", RColorBrewer::brewer.pal(12, "Paired"))
)
names(ann_colors$Noise_Level) <- annot_col$Noise_Level %>% unique

# Transcriptome
var_explained_per_factor %>%
  mutate(Model = paste(Noise_Level, Iteration, sep = "_")) %>%
  dplyr::select(Model, Variance_Explained_Transcriptome, Factor) %>%
  pivot_wider(
    names_from = Model,
    values_from = Variance_Explained_Transcriptome
  ) %>%
  column_to_rownames("Factor") %>%
  pheatmap::pheatmap(
    color = colorRampPalette(RColorBrewer::brewer.pal(n = 7, name = "YlOrRd"))(100),
    cluster_rows = FALSE,
    annotation_col = annot_col[1],
    show_colnames = FALSE,
    clustering_method = "ward.D",
    annotation_colors = ann_colors,
    cellwidth = 10,
    cellheight = 10,
    main = "Transcriptome: Explained Variance per Factor across Perturbations"
  )

# Microbiome
var_explained_per_factor %>%
  mutate(Model = paste(Noise_Level, Iteration, sep = "_")) %>%
  dplyr::select(Model, Variance_Explained_Microbiome, Factor) %>%
  pivot_wider(
    names_from = Model,
    values_from = Variance_Explained_Microbiome
  ) %>%
  column_to_rownames("Factor") %>%
  pheatmap::pheatmap(
    color = colorRampPalette(RColorBrewer::brewer.pal(n = 7, name = "YlOrRd"))(100),
    cluster_rows = FALSE,
    annotation_col = annot_col[1],
    show_colnames = FALSE,
    clustering_method = "ward.D",
    annotation_colors = ann_colors,
    cellwidth = 10,
    cellheight = 10,
    main = "Microbiome: Explained Variance per Factor across Perturbations"
  )
```

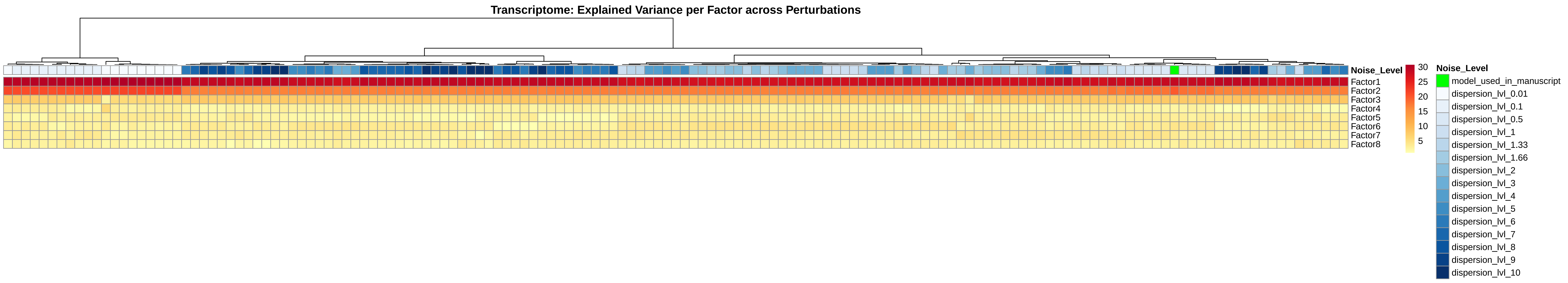

Transcriptome: Explained Variance per Factor across Perturbations
Noise_Level
Factor1
Factor2
Factor3
Factor4
Factor5
Factor6
Factor7
Factor8
30
25
20
15
10
5
Noise_Level
model_used_in_manuscript
dispersion_lvl_0.01
dispersion_lvl_0.1
dispersion_lvl_0.5
dispersion_lvl_1
dispersion_lvl_1.33
dispersion_lvl_1.66
dispersion_lvl_2
dispersion_lvl_3
dispersion_lvl_4
dispersion_lvl_5
dispersion_lvl_6
dispersion_lvl_7
dispersion_lvl_8
dispersion_lvl_9
dispersion_lvl_10

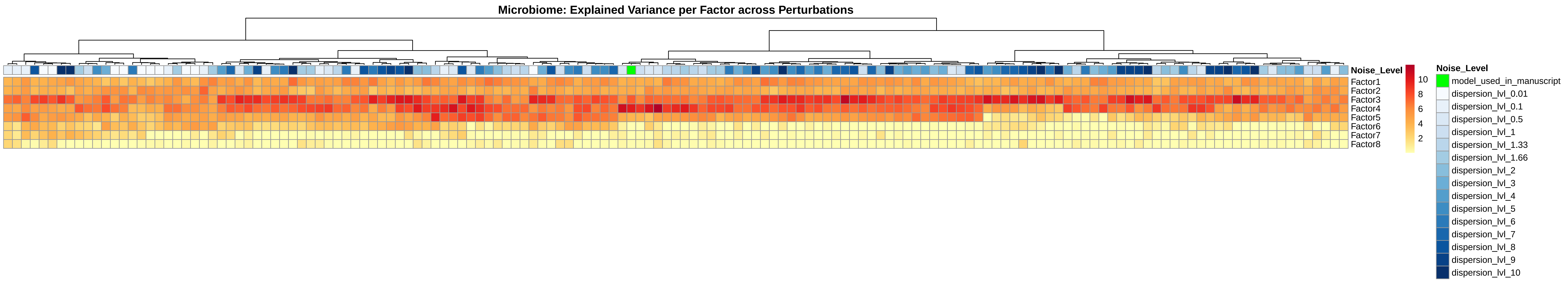

Microbiome: Explained Variance per Factor across Perturbations
Noise_Level
Factor1
Factor2
Factor3
Factor4
Factor5
Factor6
Factor7
Factor8
10
8
6
4
2
Noise_Level
model_used_in_manuscript
dispersion_lvl_0.01
dispersion_lvl_0.1
dispersion_lvl_0.5
dispersion_lvl_1
dispersion_lvl_1.33
dispersion_lvl_1.66
dispersion_lvl_2
dispersion_lvl_3
dispersion_lvl_4
dispersion_lvl_5
dispersion_lvl_6
dispersion_lvl_7
dispersion_lvl_8
dispersion_lvl_9
dispersion_lvl_10

Interpretation

The transcriptome heatmap reflects results from the violin plot of cumulated variance: The original MOFA model of the manuscript clusters togteher with the lower noise levels of 0.01 and 0.1, which was to be expected. However, the variance explained across factors and noise levels are highly similar.

Likewise, the microbiome heatmap shows clustering of the original MOFA model with lower noise levels. The pattern of variance explained is not as uniformous as in the transcriptome, which could have several reasons: Approximating appropriate technical noise is hampered by the highly zero-inflated raw data, and the significantly fewer features compared to the transcriptome dataset. Further, the microbiome could be noisier than the transcriptome. However, the heatmap shows that the majority of explained variance was captured by factors 3 and 4, similarly to the manuscript's MOFA model.

In summary, albeit the more heterogeneous pattern in the microbiome, these results suggest that the explained variance per factor was consistently captured across noise levels.

2.7.2 Comparison of latent Factors

Comparison of latent Factors aggregated across samples to a single value per latent factor

We now inspect the latent factor values of the validation models and the mansucript's original MOFA model. Therefore, latent factor value per sample is aggregated into a single value per latent factor of each model and then correlated using pearson correlation. The pearson coefficent is then plotted in a heatmap with unsupervised clustering. If the models generate similar latent factors, the heatmap should look like a block diagonal matrix, suggesting that all factors are robustly detected in all model instances.

```r
# Make annotation data.frames
LFs <- lapply(seq_along(unlist(MOFA_noise_out)), function(i) {
  do.call(rbind, get_factors( unlist(MOFA_noise_out)[[i]]))
})
samples_names <- Reduce(intersect, lapply(LFs, rownames))
LFs <- lapply(LFs, function(z) {
  z[samples_names, , drop = FALSE]
})
for (i in seq_along(LFs)) {
  colnames(LFs[[i]]) <- paste(
    names(unlist(MOFA_noise_out))[i],
    colnames(LFs[[i]]),
    sep = "_"
  )
}
corLFs <- cor(Reduce(cbind, LFs), use = "complete.obs")
corLFs[is.na(corLFs)] <- 0
corLFs <- abs(corLFs)

annot_row <- corLFs %>%
  as.data.frame() %>%
  rownames_to_column("Model") %>%
  dplyr::select(Model) %>%
  mutate(
    `Noise Level` = str_extract(Model, "^.*(?=\\.Iteration)"),
    Factor = str_extract(Model, "(?<=_)[^_]+$")
  ) %>%
  column_to_rownames("Model")
```

```r
annot_col <- corLFs %>%
  t() %>%
  as.data.frame() %>%
  rownames_to_column("Model") %>%
  dplyr::select(Model) %>%
  mutate(
    `Noise Level` = str_extract(Model, "^.*(?=\\.Iteration)"),
    Factor = str_extract(Model, "(?<=_)[^_]+$")
  ) %>%
  column_to_rownames("Model")

ann_colors <- list(
  `Noise Level` =
    colorRampPalette(RColorBrewer::brewer.pal(n = 9, name = "Blues"))(annot_col$`Noise Level` %>%
                                                                        unique %>%
                                                                        length
                                                                      ),
  Factor = RColorBrewer::brewer.pal(8, "Set1")
)
names(ann_colors$`Noise Level`) <- annot_col$`Noise Level` %>% unique
names(ann_colors$Factor) <- annot_col$Factor %>% unique

# Plot
compare_factors(
  unlist(MOFA_noise_out),
  show_rownames = FALSE,
  show_colnames = FALSE,
  annotation_row = annot_row,
  annotation_col = annot_col,
  annotation_colors = ann_colors
)
```

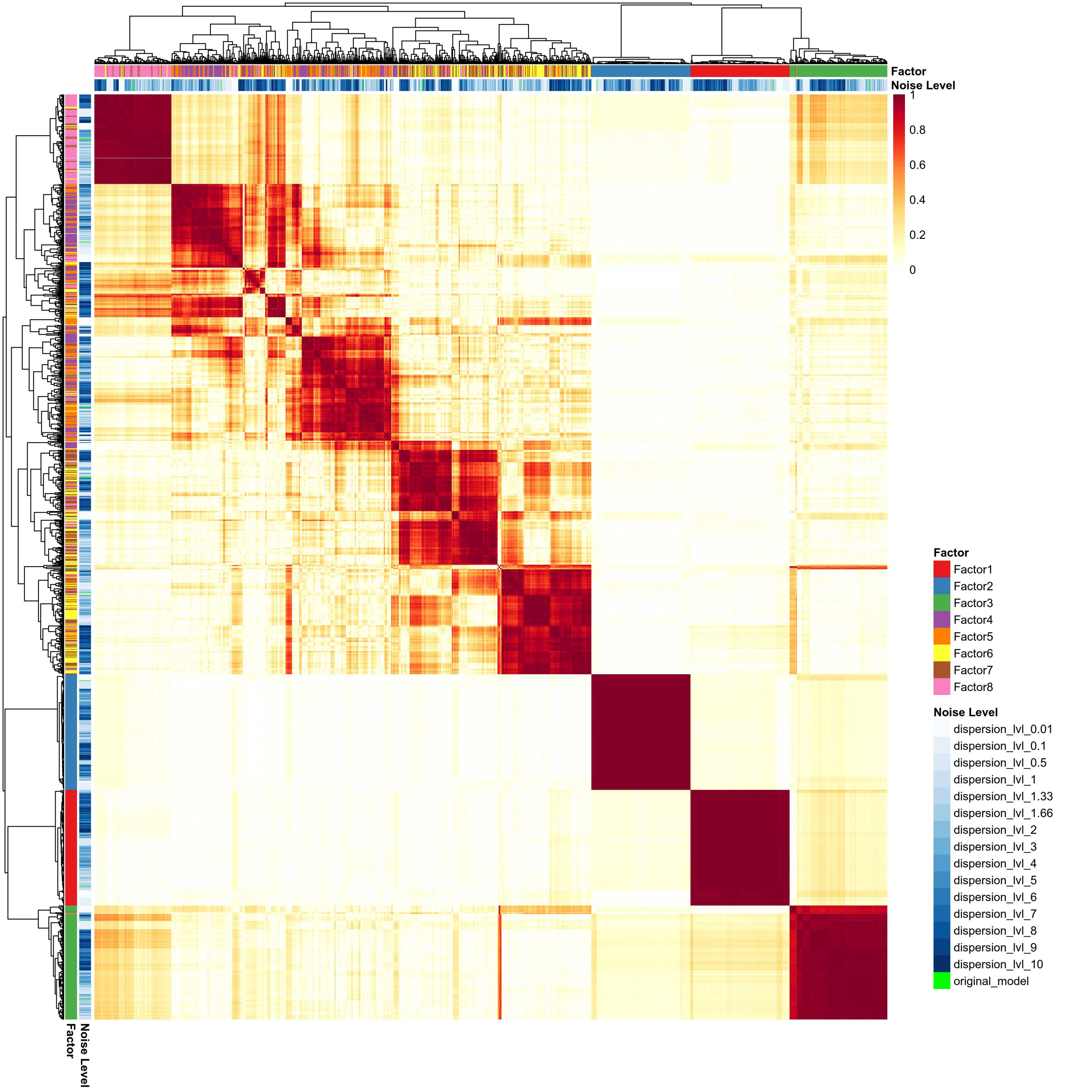
Factor
Noise Level
1
0.8
0.6
0.4
0.2
0
Factor
Factor1
Factor2
Factor3
Factor4
Factor5
Factor6
Factor7
Factor8
Noise Level
dispersion_lvl_0.01
dispersion_lvl_0.1
dispersion_lvl_0.5
dispersion_lvl_1
dispersion_lvl_1.33
dispersion_lvl_1.66
dispersion_lvl_2
dispersion_lvl_3
dispersion_lvl_4
dispersion_lvl_5
dispersion_lvl_6
dispersion_lvl_7
dispersion_lvl_8
dispersion_lvl_9
dispersion_lvl_10
original_model
Factor
Noise Level

Interpretation

Factor 1, factor 2, factor 3, and factor 8 seem to very stable across all noise levels. The first three factors explain the majority of the variance in both views In contrast, factors 6, 7, and 8 explain only little overall variance (and in fact can be neglected). Interestingly, it seems that all noise models similarly converge to assign comparable (small) values to factor 8. Factors 6 and 7, which also explain little overall variance, do not form clear individual clusters. This suggests that the introduced artificial noise is largely assigned to factors 6 and 7. Factor 4, the main factor under investigation in our manuscript, captures strong signal from the microbiome and some signal from the transcriptome. As seen in the variance explained heatmaps, this correlation plot shows that factor 4 clusters together in the lower noise levels of 0.01, 0.1, and 0.5.

This outcome suggests that the MOFA model is stable across noise levels and robustly detects the sources of variation in the two views transcriptome and microbiome.

Latent Factors per sample across noise levels

We next brake the latent factors down into a value per sample and latent factor. I.e., one value for each latent factor-sample-pair for each MOFA model (with noise and without noise). We therefore extract the latent factors per sample of each MOFA model and compare the z-scaled factors. Unsupervised clustering can reveal how stable each of the sample is to (artificial) technical noise.

```
latent_factors <- list()
scale_factors <- TRUE

for (level in names(MOFA_noise_out)) {
  for (iteration in names(MOFA_noise_out[[level]])) {
    latent_factors[[level]][[iteration]] <- get_factors(
      MOFA_noise_out[[level]][[iteration]],
      factors = "all",
      scale = scale_factors
      ) %>%
      .[[1]] %>%
      as.data.frame() %>%
      rownames_to_column("Sample") %>%
      as_tibble()
  }
}
# Add Info from original MOFA Model
latent_factors[["model_used_in_manuscript"]][["model_used_in_manuscript"]] <- get_factors(
  MOFAobject, factors = "all",
  scale = scale_factors
  )  %>%
  .[[1]] %>%
  as.data.frame() %>%
  rownames_to_column("Sample") %>%
  mutate(Sample = str_remove_all(Sample, "^[^_]*_")) %>%
  as_tibble()

# Combine into one df
latent_factors <- map_dfr(
  latent_factors,
  ~ map_dfr(.x, ~ .x, .id = "Iteration"),
  .id = "Noise Level"
)
```

```r
# Heatmap
annot_col <- latent_factors %>%
  mutate(Rowname = paste(`Noise Level`, Iteration, Sample, sep = "_") %>% str_replace_all(":", "_")) %>
  dplyr::select(Rowname, `Noise Level`, Sample) %>%
  distinct() %>%
  column_to_rownames("Rowname")

ann_colors <- list(
  `Noise Level` = colorRampPalette(RColorBrewer::brewer.pal(n = 9, name = "Blues"))(
    annot_col$`Noise Level` %>% unique %>% length -1) %>%
    c("green"),
  Sample = colorRampPalette(RColorBrewer::brewer.pal(n = 12, name = "Paired"))(
    annot_col$Sample %>% unique %>% length)
)
names(ann_colors$`Noise Level`) <- annot_col$`Noise Level` %>% unique
names(ann_colors$Sample) <- annot_col$Sample %>% unique

latent_factors %>%
  mutate(Rowname = paste(`Noise Level`, Iteration, Sample, sep = "_") %>% str_replace_all(":", "_")) %>
  column_to_rownames("Rowname") %>%
  .[, -c(1:3)] %>%
   t %>%
  pheatmap::pheatmap(
    show_colnames = FALSE,
    clustering_method = "ward.D",
    annotation_col = annot_col,
    annotation_colors = ann_colors,
    cellwidth = 0.5,
    cellheight = 8,
    main = "Latent Factors across Perturbations"
  )
```

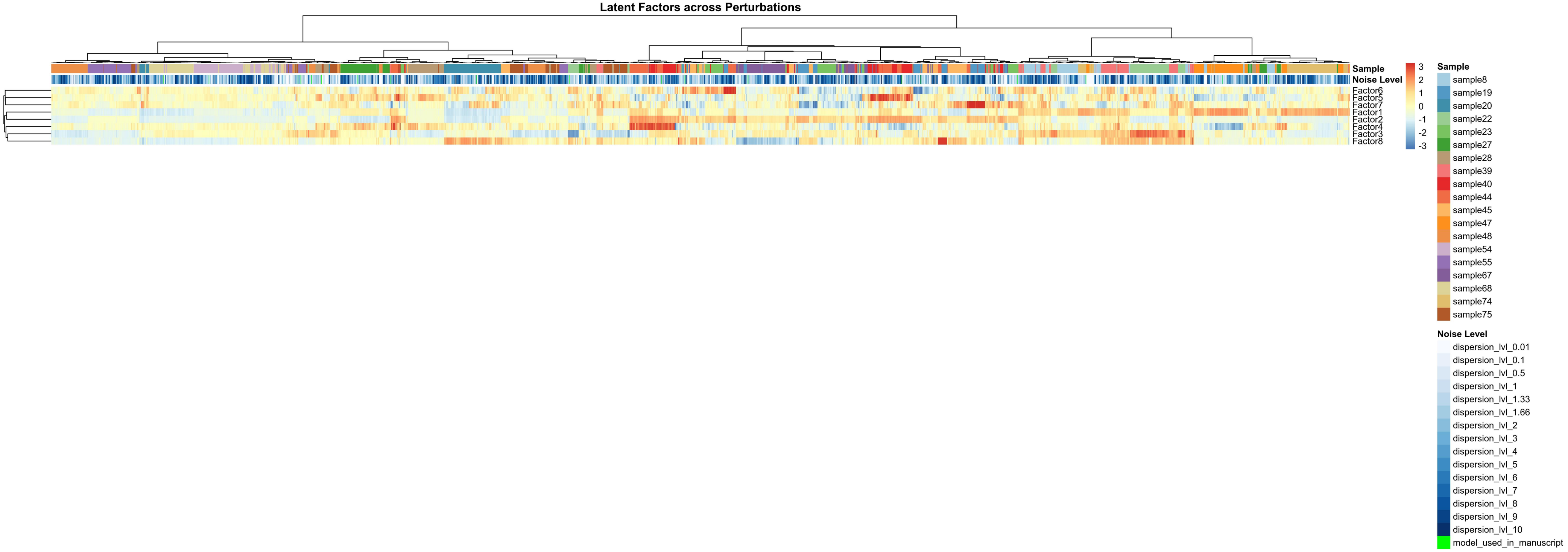

Latent Factors across Perturbations
Sample
Noise Level
Factor6
Factor5
Factor7
Factor1
Factor2
Factor4
Factor3
Factor8
3
2
1
0
-1
-2
-3
Sample
sample8
sample19
sample20
sample22
sample23
sample27
sample28
sample39
sample40
sample44
sample45
sample47
sample48
sample54
sample55
sample67
sample68
sample74
sample75
Noise Level
dispersion_lvl_0.01
dispersion_lvl_0.1
dispersion_lvl_0.5
dispersion_lvl_1
dispersion_lvl_1.33
dispersion_lvl_1.66
dispersion_lvl_2
dispersion_lvl_3
dispersion_lvl_4
dispersion_lvl_5
dispersion_lvl_6
dispersion_lvl_7
dispersion_lvl_8
dispersion_lvl_9
dispersion_lvl_10
model_used_in_manuscript

Interpretation

Most of the samples cluster together across the noise levels, suggesting that MOFA robustly assigns factor values despite introduced noise.

A similar outcome can be observed when plotting the latent factors across all samples as violion plots grouped by noise level. Non-parametric wilcoxon test shows no difference between the MOFA models with noise and the MOFA model without noise.

```
# Pivot longer
latent_factors <- latent_factors %>%
  pivot_longer(
    cols = starts_with("Factor"),
    names_to = "Factor",
    values_to = "Value"
  )

# Make violinplot
df <- latent_factors %>%
  mutate(
    x_label = factor(
      case_when(
        `Noise Level` == "model_used_in_manuscript" ~ "Original Model (no noise)",
        TRUE ~ str_remove(`Noise Level`, ".*lvl_")
      ),
      levels = c("Original Model (no noise)", unique(latent_factors$`Noise Level`) %>% str_remove(".*lvl[illegible]
    )
  )

df %>%
  ggplot(aes(x = x_label, y = Value)) +
  geom_violin(drop = FALSE) +
  geom_point() +
  labs(x = element_blank(), y = "Latent Factor Value") +
  ggsignif::geom_signif(
    comparisons = list(
      c("Original Model (no noise)", "0.01"),
      c("Original Model (no noise)", "0.1"),
      c("Original Model (no noise)", "0.5"),
      c("Original Model (no noise)", "1"),
      c("Original Model (no noise)", "1.33"),
      c("Original Model (no noise)", "1.66"),
      c("Original Model (no noise)", "2"),
      c("Original Model (no noise)", "3"),
      c("Original Model (no noise)", "4"),
      c("Original Model (no noise)", "5"),
      c("Original Model (no noise)", "6"),
      c("Original Model (no noise)", "7"),
      c("Original Model (no noise)", "8"),
      c("Original Model (no noise)", "9"),
      c("Original Model (no noise)", "10")
    ),
    map_signif_level = TRUE,
    y_position = seq(2.5, 9.5, by = 0.5)
  ) +
```

```
  theme_minimal() +
  theme(axis.text.x = element_text(angle = 90, hjust = 1, vjust = 0.2)) +
  facet_wrap("Factor")
```

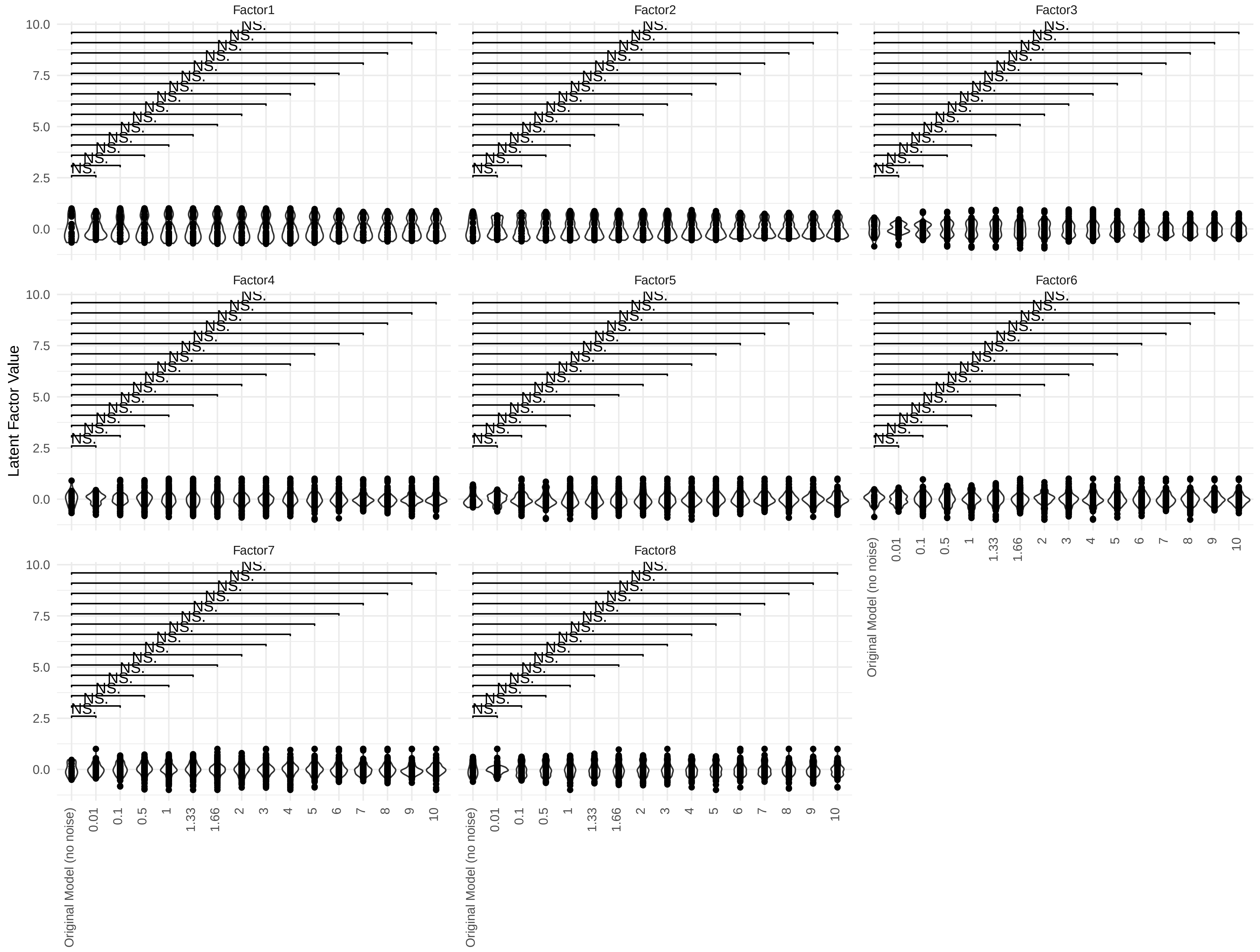
Factor1
Factor2
Factor3
Factor4
Factor5
Factor6
Factor7
Factor8
Latent Factor Value
10.0
7.5
5.0
2.5
0.0
NS.
Original Model (no noise)
0.01
0.1
0.5
1
1.33
1.66
2
3
4
5
6
7
8
9
10

We can also aggregate the latent factors of the 10 iterations per noise level into 1 median latent factor value per noise level. We then plot a heatmap of these median latent factor values per sample across the noise levels and the orignal noise level.

```
# Calculate the median

# Iterate over unique Noise Levels
latent_factors_median <- list()

for (level in unique(latent_factors$`Noise Level`)) {
  # Initialize a sub-list for each Noise Level
  latent_factors_median[[level]] <- list()

  # Filter data for the current Noise Level
  filtered_level <- latent_factors %>% dplyr::filter(`Noise Level` == level)

  # Iterate over unique Factors
  for (Fact in unique(filtered_level$Factor)) {
    # Initialize a sub-list for each Factor
    latent_factors_median[[level]][[Fact]] <- list()

    # Filter data for the current Factor
    filtered_fact <- filtered_level %>% dplyr::filter(Factor == Fact)

    # Iterate over unique Samples
    for (sam in unique(filtered_fact$Sample)) {
      # Filter data for the current Sample
      filtered_sample <- filtered_fact %>% dplyr::filter(Sample == sam)

      # Store the filtered sample data directly
      latent_factors_median[[level]][[Fact]][[sam]] <- filtered_sample %>%
        pull(Value) %>%
        median()
    }
  }
}

# Combine in 1 tibble
latent_factors_median <- map_dfr(names(latent_factors_median), function(level) {
  map_dfr(names(latent_factors_median[[level]]), function(Fact) {
    map_dfr(names(latent_factors_median[[level]][[Fact]]), function(sam) {
      tibble(
        `Noise Level` = level,
        Factor = Fact,
        Sample = sam,
        Median_Value = latent_factors_median[[level]][[Fact]][[sam]]
      )
    })
  })
})


# Heatmap
annot_col <- latent_factors_median %>%
```

```r
  mutate(Model = paste(`Noise Level`, Factor, sep = "_")) %>%
  dplyr::select(Model, `Noise Level`, Factor) %>%
  distinct() %>%
  column_to_rownames("Model")

ann_colors <- list(
  `Noise Level` = colorRampPalette(RColorBrewer::brewer.pal(n = 9, name = "Blues"))(
    annot_col$`Noise Level` %>% unique %>% length -1) %>%
    c("green"),
    Factor = RColorBrewer::brewer.pal(8, "Set1")
)
names(ann_colors$`Noise Level`) <- annot_col$`Noise Level` %>% unique
names(ann_colors$Factor) <- annot_col$Factor %>% unique

# Make wider format for plotting
latent_factors_median %>%
  pivot_wider(
    names_from = Sample,
    values_from = Median_Value
  ) %>%
  mutate(Combi = paste(`Noise Level`, Factor, sep = "_")) %>%
  dplyr::select(!c(`Noise Level`, Factor)) %>%
  column_to_rownames("Combi") %>%
  t() %>%
  # Make the heatpmap
  pheatmap::pheatmap(
    cluster_rows = TRUE,
    annotation_col = annot_col,
    show_colnames = FALSE,
    show_rownames = TRUE,
    clustering_method = "ward.D",
    annotation_colors = ann_colors,
    cellwidth = 10,
    cellheight = 10,
    main = "Latent Factors (Median Values) across Perturbations",
    scale = "column"
  )
```

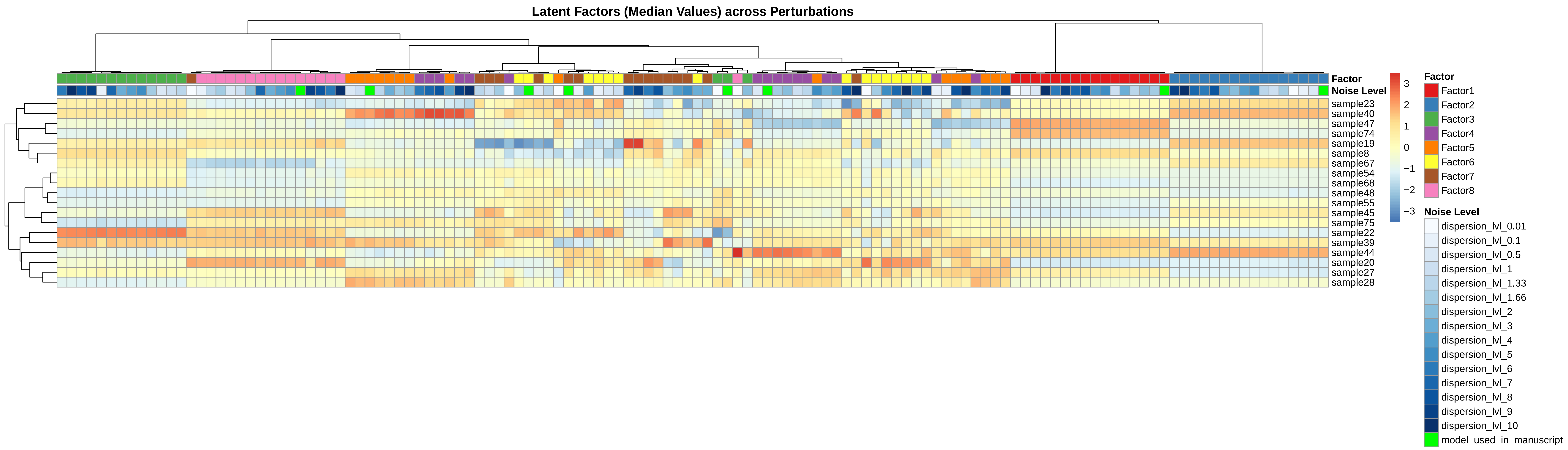

Latent Factors (Median Values) across Perturbations
Factor
Noise Level
sample23
sample40
sample47
sample74
sample19
sample8
sample67
sample54
sample68
sample48
sample55
sample45
sample75
sample22
sample39
sample44
sample20
sample27
sample28
3
2
1
0
−1
−2
−3
Factor
Factor1
Factor2
Factor3
Factor4
Factor5
Factor6
Factor7
Factor8
Noise Level
dispersion_lvl_0.01
dispersion_lvl_0.1
dispersion_lvl_0.5
dispersion_lvl_1
dispersion_lvl_1.33
dispersion_lvl_1.66
dispersion_lvl_2
dispersion_lvl_3
dispersion_lvl_4
dispersion_lvl_5
dispersion_lvl_6
dispersion_lvl_7
dispersion_lvl_8
dispersion_lvl_9
dispersion_lvl_10
model_used_in_manuscript

Interpretation

We observe a similar result as for the non-agregated heatmap of latent factor values per sample across noise levels. Factor 1, factor 2, factor 3, and factor 8 seem to very stable across all noise levels. The first three factors explain the majority of the variance in both views In contrast, factors 6, 7, and 8 explain only little overall variance (and in fact can be neglected). Interestingly, it seems that MOFA stably assigned small values to latent factor 8. Conversely, the majority of variation introduced by the artificial noise is captured in factors 6 and 7, which cluster together in various higher noise levels. Factor 4, the main factor under investigation in our manuscript, captures strong signal from the microbiome and some signal from the transcriptome. As seen in the variance explained heatmaps, this correlation plot shows that factor 4 clusters together in the lower noise levels of from 0.01 to 2.

2.7.3 Comparison of ELBO values

We next compare the Evidene of Lower Bound (ELBO) across the MOFA models. The ELBO is a metric used in variational inference to approximate the log likelihood of a model. In statistical modeling, the ELBO is used to assess how well the model explains the data while avoiding overfitting. A higher ELBO indicates a better fit between the model and the data.

```
elbo_compare <- compare_elbo(
  unlist(MOFA_noise_out) %>% append(list(original_model_used_in_manuscript = MOFAobject)),
  return_data = TRUE
) %>%
  mutate(
    x_label = case_when(
      model == "original_model_used_in_manuscript" ~ "Original Model (no noise)",
      TRUE ~ str_extract(model,  "(?<=lvl_)[0-9.]+(?=\\.Iteration)")
    )
  ) %>%
  dplyr::slice(n(), 1:(n() - 1))

elbo_compare %>%
  ggplot(aes(model, ELBO)) +
  geom_bar(stat = "identity", color = "black", fill = "grey70") +
  labs(x = element_blank(), y = "Evidence Lower Bound (ELBO)") +
  scale_x_discrete(labels = elbo_compare$x_label) +
  theme_classic() +
  theme(axis.text.x = element_text(angle = 90, hjust = 1, vjust = 0.2)) +
  ggtitle(
    "Absolute ELBO value for each model. The higher the value, the better the fit.",
    subtitle = "All models have a relatively similar ELBO value, indicating that MOFA is robust to nois
  )
```

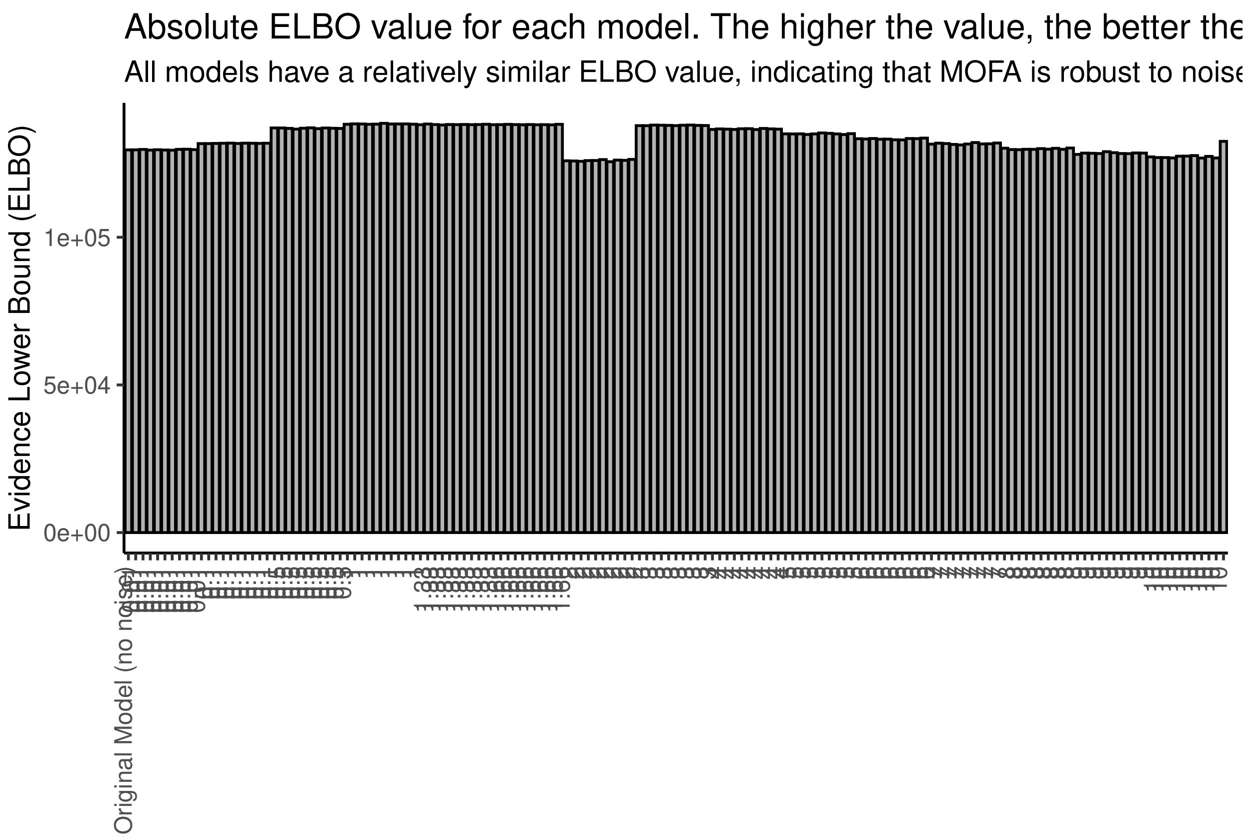

Interpretation

All Models have relatively similar ELBO values, with noise levels 0.01 and 0.1 again being among the most akin. This suggests that MOFA fits the data similarly well across noise levels.

References

Argelaguet, Ricard, Britta Velten, Damien Arnol, Sascha Dietrich, Thorsten Zenz, John C Marioni, Florian Buettner, Wolfgang Huber, and Oliver Stegle. 2018. "Multi-Omics Factor Analysis—a Framework for Unsupervised Integration of Multi-Omics Data Sets." *Molecular Systems Biology* 14 (6): e8124.

Kong, Heidi H, Björn Andersson, Thomas Clavel, John E Common, Scott A Jackson, Nathan D Olson, Julia A Segre, and Claudia Traidl-Hoffmann. 2017. "Performing Skin Microbiome Research: A Method to the Madness." *Journal of Investigative Dermatology* 137 (3): 561–68.

Kumar, M Senthil, Eric V Slud, Kwame Okrah, Stephanie C Hicks, Sridhar Hannenhalli, and Héctor Corrada Bravo. 2018. "Analysis and Correction of Compositional Bias in Sparse Sequencing Count Data." *BMC Genomics* 19: 1–23.

Licht, Philipp, Nazzareno Dominelli, Johannes Kleemann, Stefan Pastore, Elena-Sophia Müller, Maximilian Haist, Kim Sophie Hartmann, et al. 2024. "The Skin Microbiome Stratifies Patients with Cutaneous t Cell Lymphoma and Determines Event-Free Survival." *Npj Biofilms and Microbiomes* 10 (1): 74.

Mallick, Himel, Ali Rahnavard, Lauren J McIver, Siyuan Ma, Yancong Zhang, Long H Nguyen, Timothy L Tickle, et al. 2021. "Multivariable Association Discovery in Population-Scale Meta-Omics Studies." *PLoS Computational Biology* 17 (11): e1009442.

Oh, Julia, Allyson L Byrd, Clay Deming, Sean Conlan, Heidi H Kong, and Julia A Segre. 2014. "Biogeography and Individuality Shape Function in the Human Skin Metagenome." *Nature* 514 (7520): 59–64.

Tett, Adrian, Edoardo Pasolli, Stefania Farina, Duy Tin Truong, Francesco Asnicar, Moreno Zolfo, Francesco Beghini, et al. 2017. "Unexplored Diversity and Strain-Level Structure of the Skin Microbiome Associated with Psoriasis." *NPJ Biofilms and Microbiomes* 3 (1): 14.
