# Supplementary material for: Multi-Omic Data Integration Suggests Putative Microbial Drivers of Aetiopathogenesis in Mycosis Fungoides
Source: Cancers (Basel). 2024 Nov 25;16(23):3947. doi: 10.3390/cancers16233947 (PMC11640326; doi:10.3390/cancers16233947)
Supplement: Supplementary file 1 [file cancers-16-03947-s001.zip › Suppl_Material3.zip!/Patch_GSEA_EnrichmentComparison.pdf]

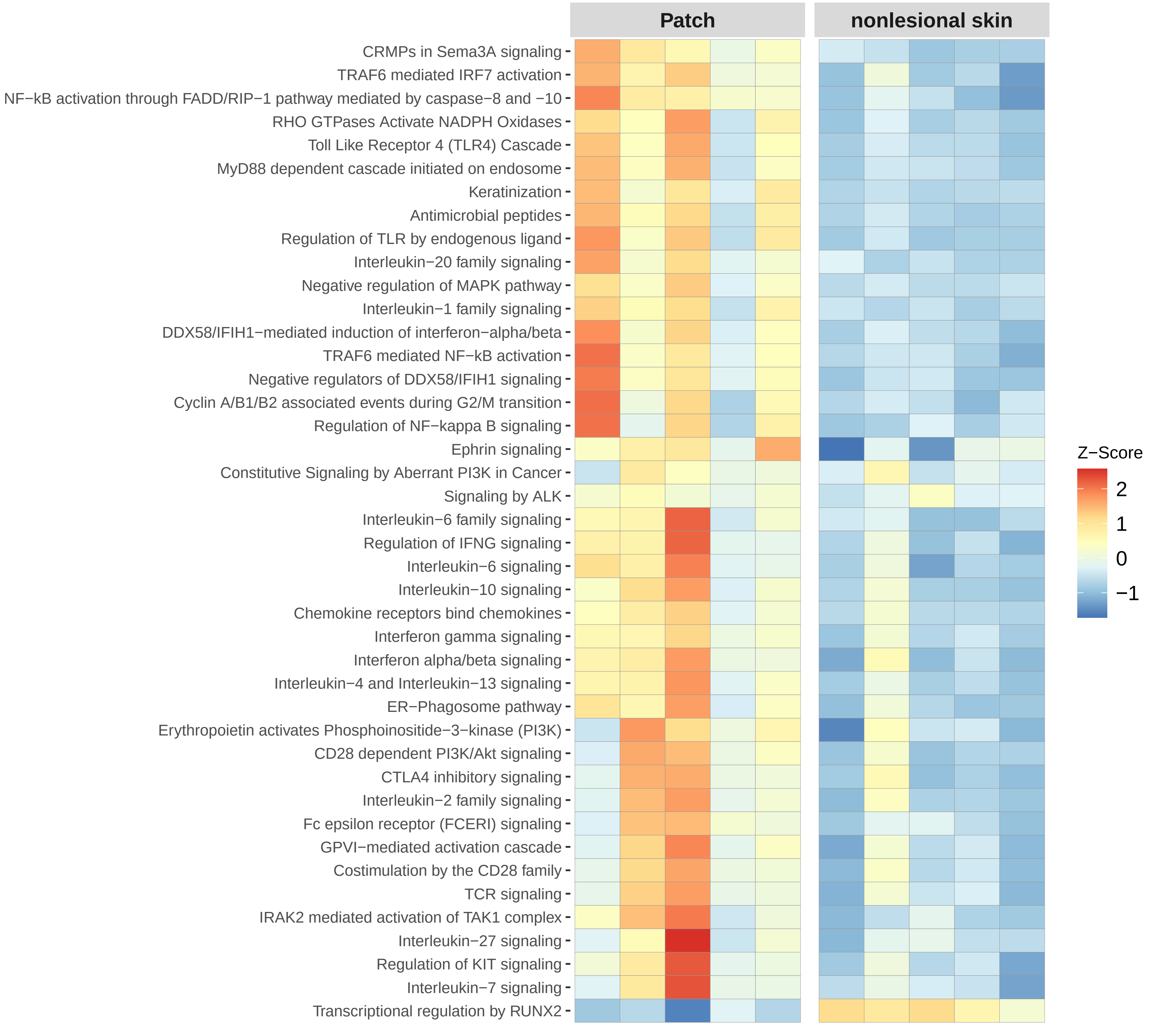

Patch
nonlesional skin
CRMPs in Sema3A signaling
TRAF6 mediated IRF7 activation
NF−kB activation through FADD/RIP−1 pathway mediated by caspase−8 and −10
RHO GTPases Activate NADPH Oxidases
Toll Like Receptor 4 (TLR4) Cascade
MyD88 dependent cascade initiated on endosome
Keratinization
Antimicrobial peptides
Regulation of TLR by endogenous ligand
Interleukin−20 family signaling
Negative regulation of MAPK pathway
Interleukin−1 family signaling
DDX58/IFIH1−mediated induction of interferon−alpha/beta
TRAF6 mediated NF−kB activation
Negative regulators of DDX58/IFIH1 signaling
Cyclin A/B1/B2 associated events during G2/M transition
Regulation of NF−kappa B signaling
Ephrin signaling
Constitutive Signaling by Aberrant PI3K in Cancer
Signaling by ALK
Interleukin−6 family signaling
Regulation of IFNG signaling
Interleukin−6 signaling
Interleukin−10 signaling
Chemokine receptors bind chemokines
Interferon gamma signaling
Interferon alpha/beta signaling
Interleukin−4 and Interleukin−13 signaling
ER−Phagosome pathway
Erythropoietin activates Phosphoinositide−3−kinase (PI3K)
CD28 dependent PI3K/Akt signaling
CTLA4 inhibitory signaling
Interleukin−2 family signaling
Fc epsilon receptor (FCERI) signaling
GPVI−mediated activation cascade
Costimulation by the CD28 family
TCR signaling
IRAK2 mediated activation of TAK1 complex
Interleukin−27 signaling
Regulation of KIT signaling
Interleukin−7 signaling
Transcriptional regulation by RUNX2
Z−Score
2
1
0
−1
