# Supplementary material for: Multi-Omic Data Integration Suggests Putative Microbial Drivers of Aetiopathogenesis in Mycosis Fungoides
Source: Cancers (Basel). 2024 Nov 25;16(23):3947. doi: 10.3390/cancers16233947 (PMC11640326; doi:10.3390/cancers16233947)
Supplement: Supplementary file 1 [file cancers-16-03947-s001.zip › Suppl_Material3.zip!/Plaque_GSEA_EnrichmentComparison.pdf.pdf]

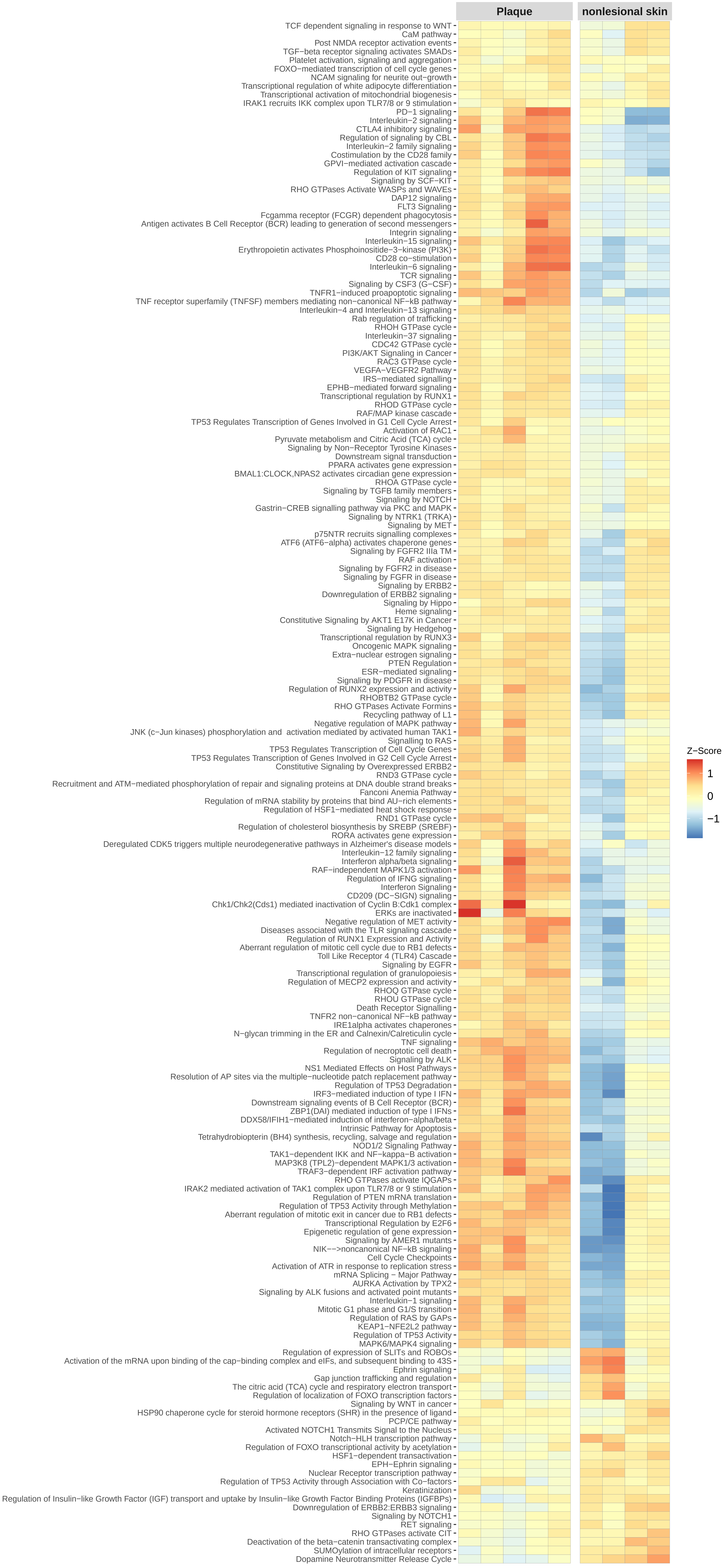
Plaque
nonlesional skin
TCF dependent signaling in response to WNT
CaM pathway
Post NMDA receptor activation events
TGF−beta receptor signaling activates SMADs
Platelet activation, signaling and aggregation
FOXO−mediated transcription of cell cycle genes
NCAM signaling for neurite out−growth
Transcriptional regulation of white adipocyte differentiation
Transcriptional activation of mitochondrial biogenesis
IRAK1 recruits IKK complex upon TLR7/8 or 9 stimulation
PD−1 signaling
Interleukin−2 signaling
CTLA4 inhibitory signaling
Regulation of signaling by CBL
Interleukin−2 family signaling
Costimulation by the CD28 family
GPVI−mediated activation cascade
Regulation of KIT signaling
Signaling by SCF−KIT
RHO GTPases Activate WASPs and WAVEs
DAP12 signaling
FLT3 Signaling
Fcgamma receptor (FCGR) dependent phagocytosis
Antigen activates B Cell Receptor (BCR) leading to generation of second messengers
Integrin signaling
Interleukin−15 signaling
Erythropoietin activates Phosphoinositide−3−kinase (PI3K)
CD28 co−stimulation
Interleukin−6 signaling
TCR signaling
Signaling by CSF3 (G−CSF)
TNFR1−induced proapoptotic signaling
TNF receptor superfamily (TNFSF) members mediating non−canonical NF−kB pathway
Interleukin−4 and Interleukin−13 signaling
Rab regulation of trafficking
RHOH GTPase cycle
Interleukin−37 signaling
CDC42 GTPase cycle
PI3K/AKT Signaling in Cancer
RAC3 GTPase cycle
VEGFA−VEGFR2 Pathway
IRS−mediated signalling
EPHB−mediated forward signaling
Transcriptional regulation by RUNX1
RHOD GTPase cycle
RAF/MAP kinase cascade
TP53 Regulates Transcription of Genes Involved in G1 Cell Cycle Arrest
Activation of RAC1
Pyruvate metabolism and Citric Acid (TCA) cycle
Signaling by Non−Receptor Tyrosine Kinases
Downstream signal transduction
PPARA activates gene expression
BMAL1:CLOCK,NPAS2 activates circadian gene expression
RHOA GTPase cycle
Signaling by TGFB family members
Signaling by NOTCH
Gastrin−CREB signalling pathway via PKC and MAPK
Signaling by NTRK1 (TRKA)
Signaling by MET
p75NTR recruits signalling complexes
ATF6 (ATF6−alpha) activates chaperone genes
Signaling by FGFR2 IIIa TM
RAF activation
Signaling by FGFR2 in disease
Signaling by FGFR in disease
Signaling by ERBB2
Downregulation of ERBB2 signaling
Signaling by Hippo
Heme signaling
Constitutive Signaling by AKT1 E17K in Cancer
Signaling by Hedgehog
Transcriptional regulation by RUNX3
Oncogenic MAPK signaling
Extra−nuclear estrogen signaling
PTEN Regulation
ESR−mediated signaling
Signaling by PDGFR in disease
Regulation of RUNX2 expression and activity
RHOBTB2 GTPase cycle
RHO GTPases Activate Formins
Recycling pathway of L1
Negative regulation of MAPK pathway
JNK (c−Jun kinases) phosphorylation and activation mediated by activated human TAK1
Signalling to RAS
TP53 Regulates Transcription of Cell Cycle Genes
TP53 Regulates Transcription of Genes Involved in G2 Cell Cycle Arrest
Constitutive Signaling by Overexpressed ERBB2
RND3 GTPase cycle
Recruitment and ATM−mediated phosphorylation of repair and signaling proteins at DNA double strand breaks
Fanconi Anemia Pathway
Regulation of mRNA stability by proteins that bind AU−rich elements
Regulation of HSF1−mediated heat shock response
RND1 GTPase cycle
Regulation of cholesterol biosynthesis by SREBP (SREBF)
RORA activates gene expression
Deregulated CDK5 triggers multiple neurodegenerative pathways in Alzheimer's disease models
Interleukin−12 family signaling
Interferon alpha/beta signaling
RAF−independent MAPK1/3 activation
Regulation of IFNG signaling
Interferon Signaling
CD209 (DC−SIGN) signaling
Chk1/Chk2(Cds1) mediated inactivation of Cyclin B:Cdk1 complex
ERKs are inactivated
Negative regulation of MET activity
Diseases associated with the TLR signaling cascade
Regulation of RUNX1 Expression and Activity
Aberrant regulation of mitotic cell cycle due to RB1 defects
Toll Like Receptor 4 (TLR4) Cascade
Signaling by EGFR
Transcriptional regulation of granulopoiesis
Regulation of MECP2 expression and activity
RHOQ GTPase cycle
RHOU GTPase cycle
Death Receptor Signalling
TNFR2 non−canonical NF−kB pathway
IRE1alpha activates chaperones
N−glycan trimming in the ER and Calnexin/Calreticulin cycle
TNF signaling
Regulation of necroptotic cell death
Signaling by ALK
NS1 Mediated Effects on Host Pathways
Resolution of AP sites via the multiple−nucleotide patch replacement pathway
Regulation of TP53 Degradation
IRF3−mediated induction of type I IFN
Downstream signaling events of B Cell Receptor (BCR)
ZBP1(DAI) mediated induction of type I IFNs
DDX58/IFIH1−mediated induction of interferon−alpha/beta
Intrinsic Pathway for Apoptosis
Tetrahydrobiopterin (BH4) synthesis, recycling, salvage and regulation
NOD1/2 Signaling Pathway
TAK1−dependent IKK and NF−kappa−B activation
MAP3K8 (TPL2)−dependent MAPK1/3 activation
TRAF3−dependent IRF activation pathway
RHO GTPases activate IQGAPs
IRAK2 mediated activation of TAK1 complex upon TLR7/8 or 9 stimulation
Regulation of PTEN mRNA translation
Regulation of TP53 Activity through Methylation
Aberrant regulation of mitotic exit in cancer due to RB1 defects
Transcriptional Regulation by E2F6
Epigenetic regulation of gene expression
Signaling by AMER1 mutants
NIK−−>noncanonical NF−kB signaling
Cell Cycle Checkpoints
Activation of ATR in response to replication stress
mRNA Splicing − Major Pathway
AURKA Activation by TPX2
Signaling by ALK fusions and activated point mutants
Interleukin−1 signaling
Mitotic G1 phase and G1/S transition
Regulation of RAS by GAPs
KEAP1−NFE2L2 pathway
Regulation of TP53 Activity
MAPK6/MAPK4 signaling
Regulation of expression of SLITs and ROBOs
Activation of the mRNA upon binding of the cap−binding complex and eIFs, and subsequent binding to 43S
Ephrin signaling
Gap junction trafficking and regulation
The citric acid (TCA) cycle and respiratory electron transport
Regulation of localization of FOXO transcription factors
Signaling by WNT in cancer
HSP90 chaperone cycle for steroid hormone receptors (SHR) in the presence of ligand
PCP/CE pathway
Activated NOTCH1 Transmits Signal to the Nucleus
Notch−HLH transcription pathway
Regulation of FOXO transcriptional activity by acetylation
HSF1−dependent transactivation
EPH−Ephrin signaling
Nuclear Receptor transcription pathway
Regulation of TP53 Activity through Association with Co−factors
Keratinization
Regulation of Insulin−like Growth Factor (IGF) transport and uptake by Insulin−like Growth Factor Binding Proteins (IGFBPs)
Downregulation of ERBB2:ERBB3 signaling
Signaling by NOTCH1
RET signaling
RHO GTPases activate CIT
Deactivation of the beta−catenin transactivating complex
SUMOylation of intracellular receptors
Dopamine Neurotransmitter Release Cycle
Z−Score
1
0
−1
