# Supplementary material for: Multi-Omic Data Integration Suggests Putative Microbial Drivers of Aetiopathogenesis in Mycosis Fungoides
Source: Cancers (Basel). 2024 Nov 25;16(23):3947. doi: 10.3390/cancers16233947 (PMC11640326; doi:10.3390/cancers16233947)

Enriched Pathways and genes with the highest weight in each pathway of Factor 1

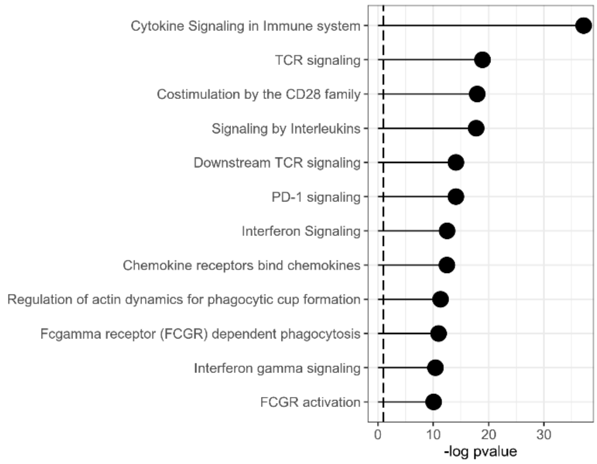

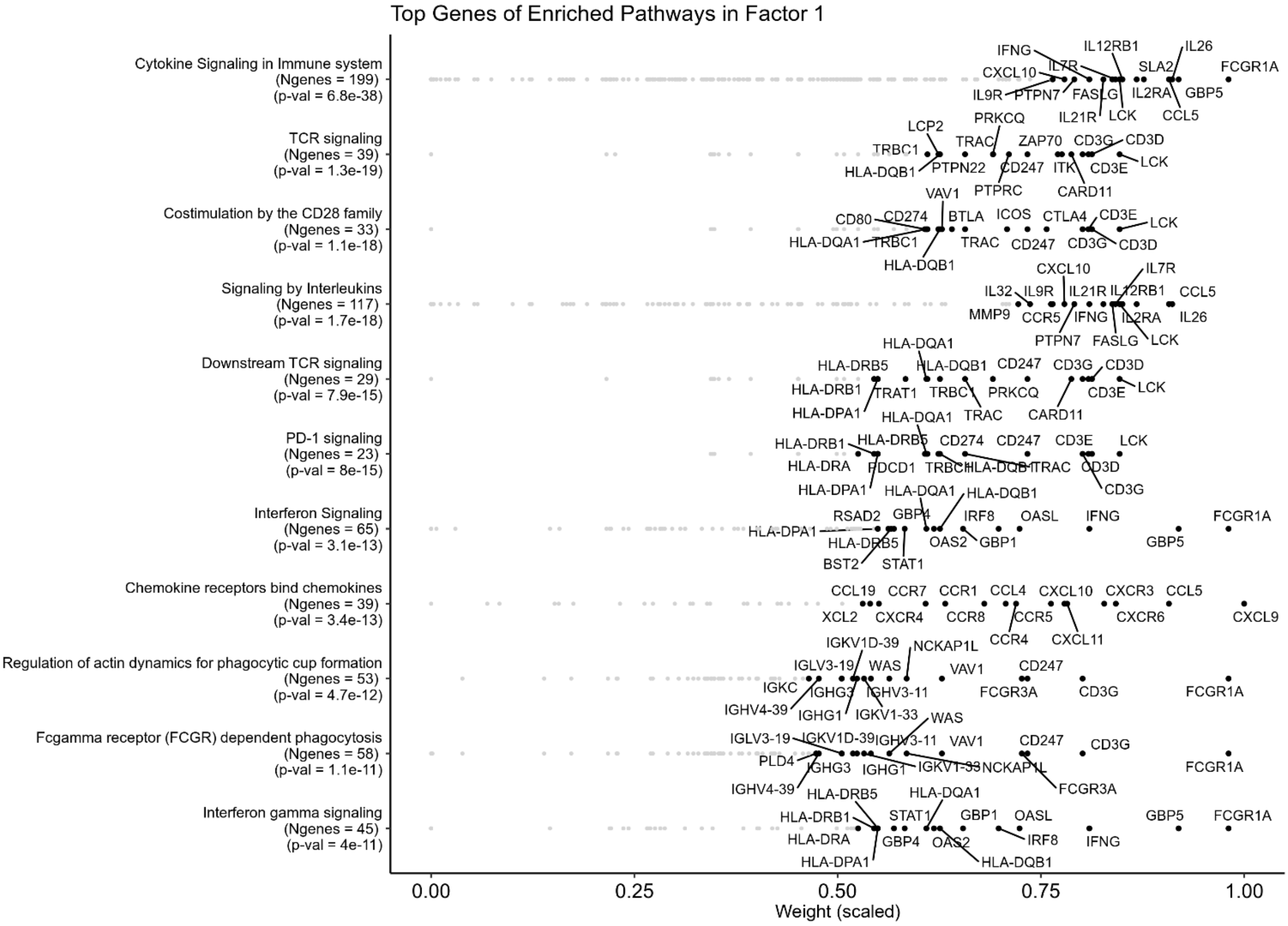

Enriched Pathways and genes with the highest weight in each pathway of Factor 2

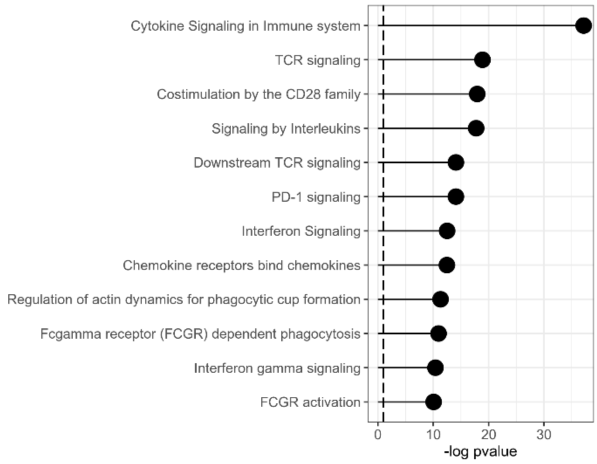

Top Genes of Enriched Pathways in Factor 2

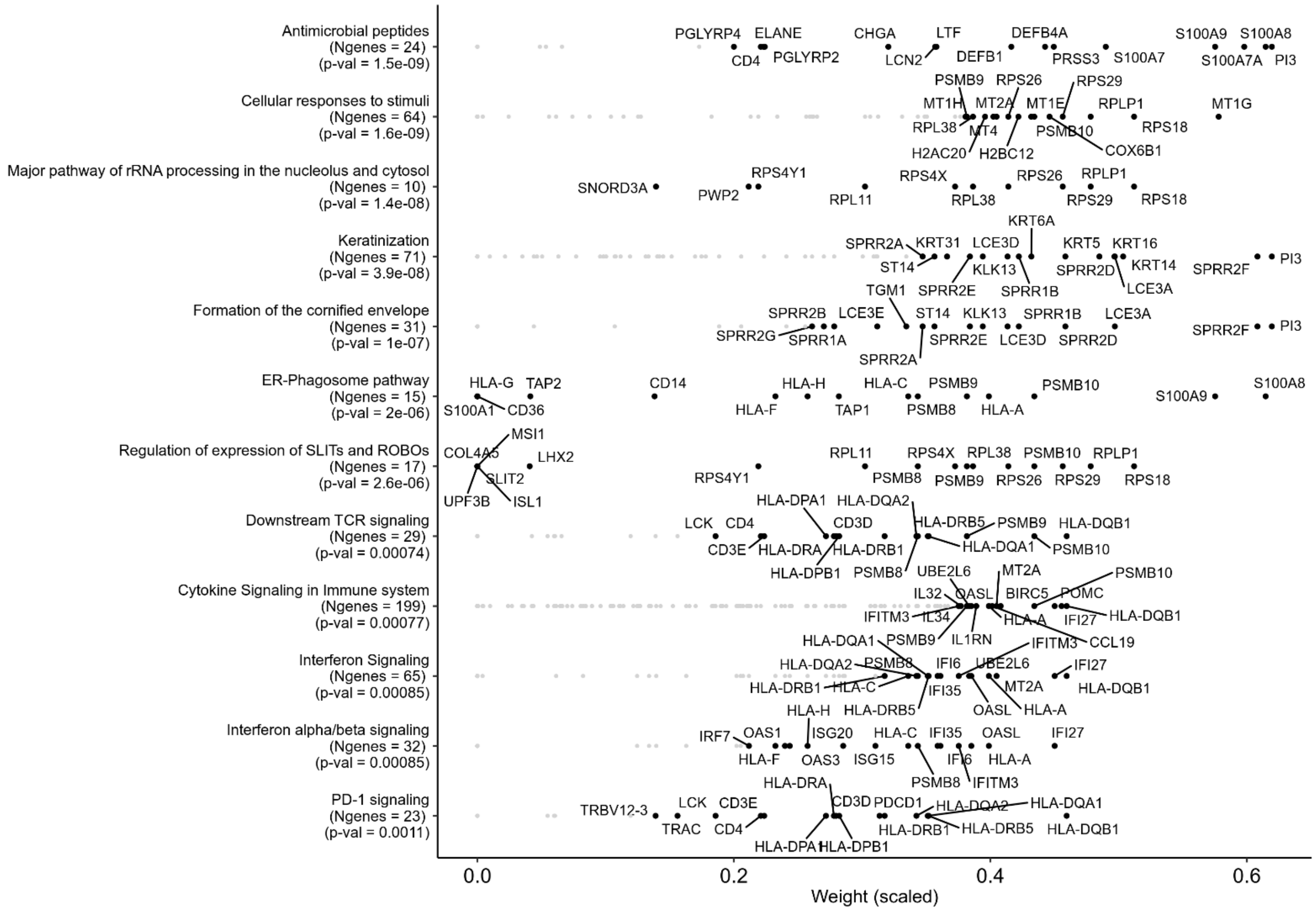

Enriched Pathways and genes with the highest weight in each pathway of Factor 3

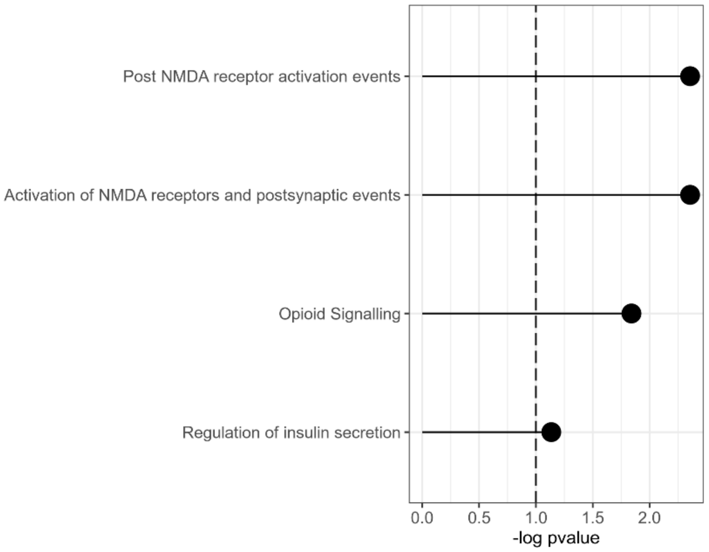

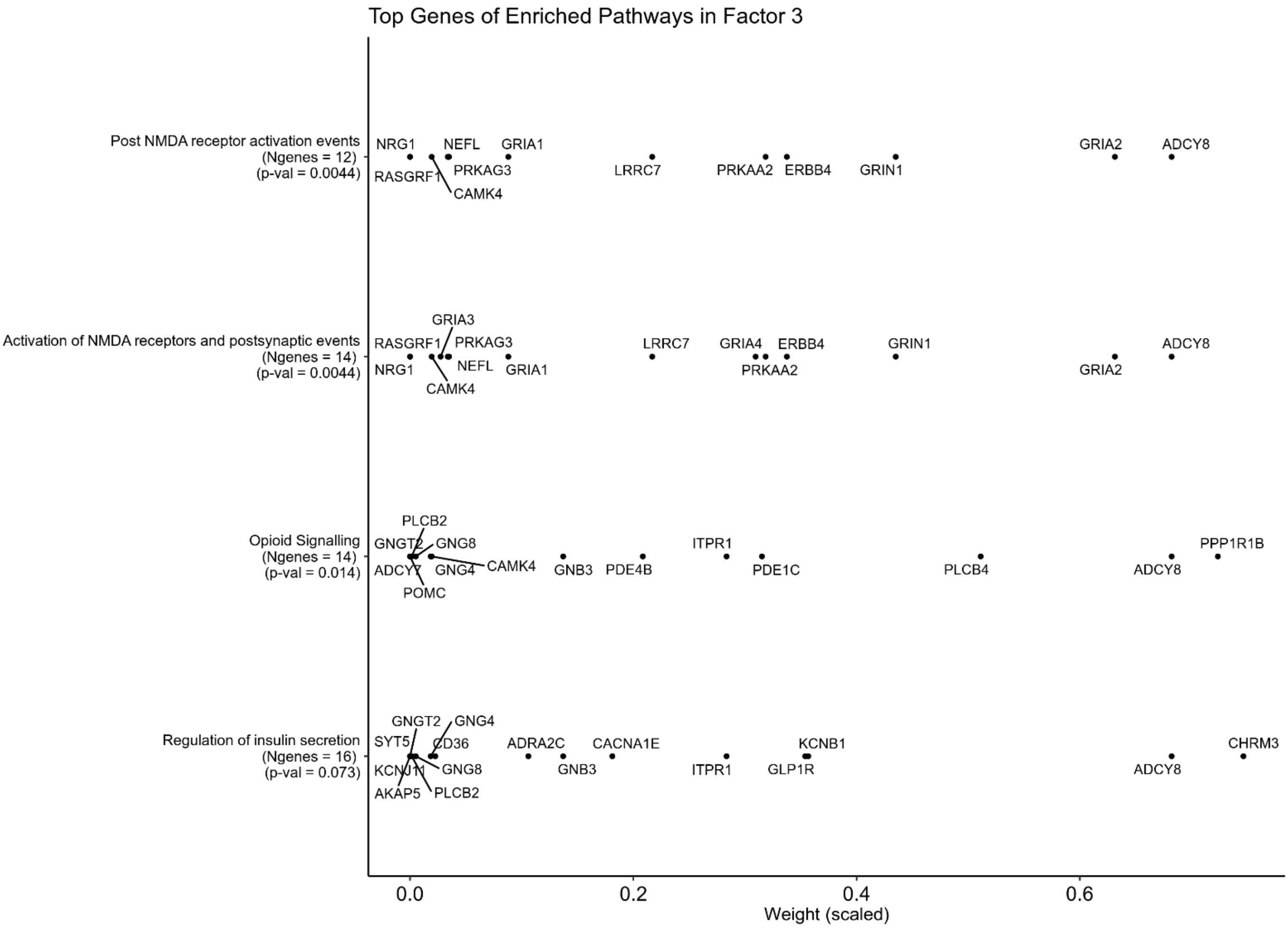

Enriched Pathways and genes with the highest weight in each pathway of Factor 4

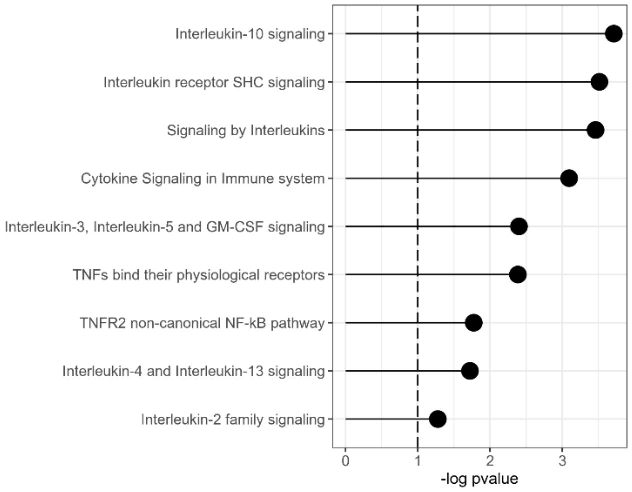

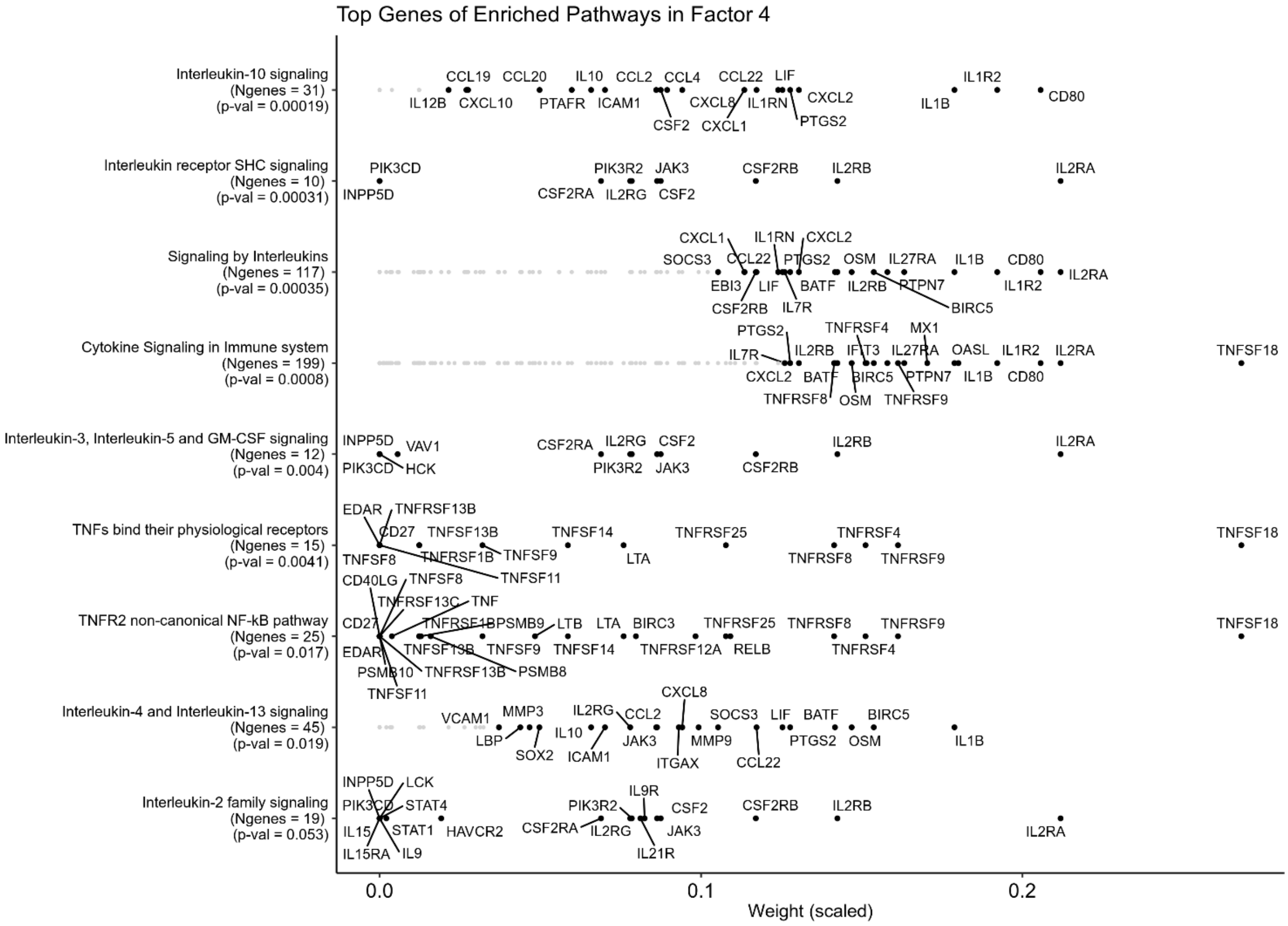

Enriched Pathways and genes with the highest weight in each pathway of Factor 5

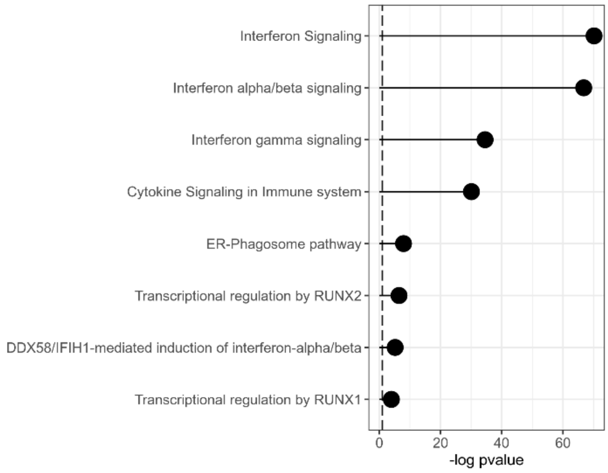

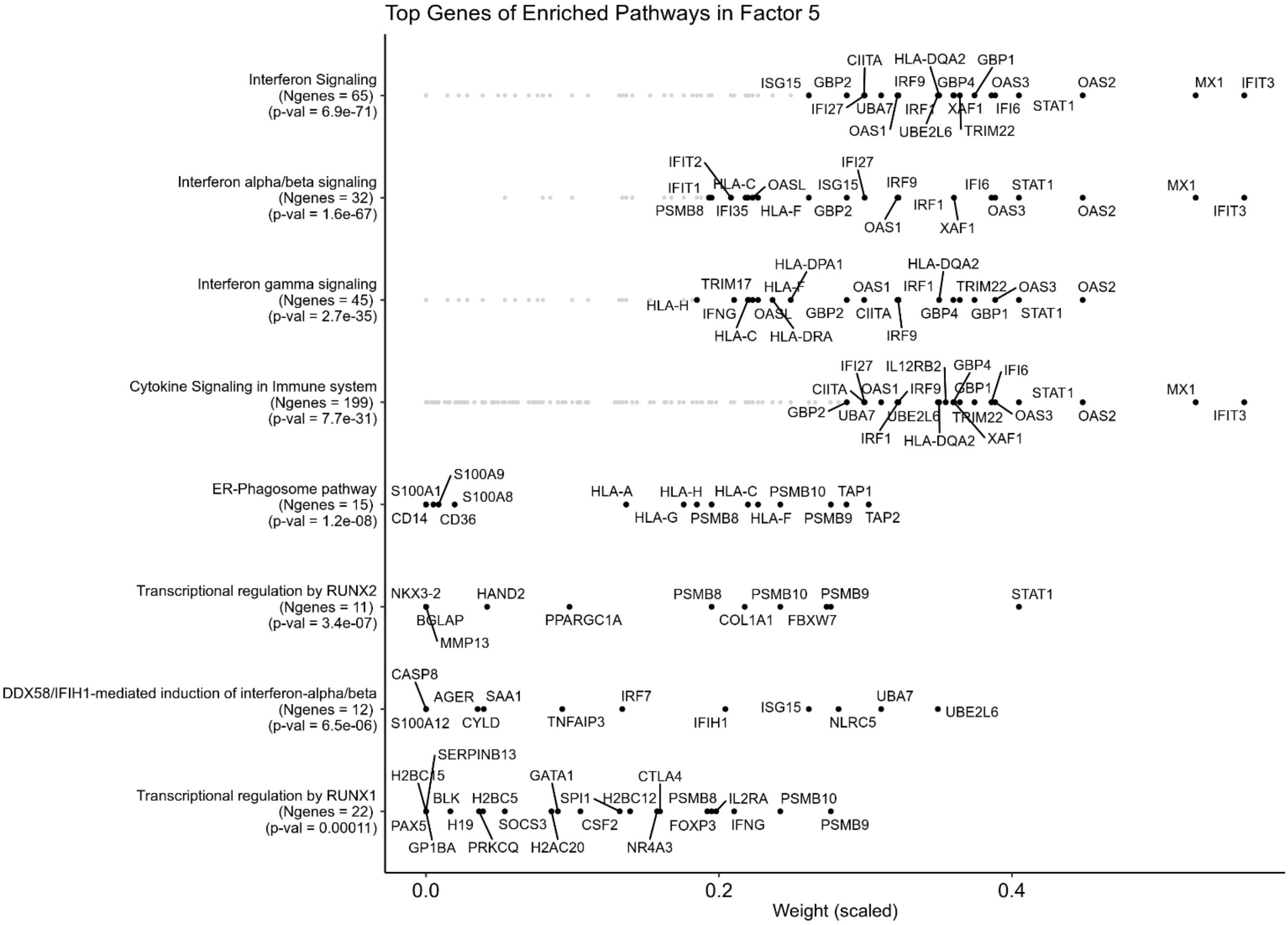

Supplement: Supplementary file 1 [file cancers-16-03947-s001.zip › Suppl_Material5.zip!/Suppl_Material5_Plots.pdf]
